# Synthesis of Multisubstituted Benzimidazolones via Copper-Catalyzed Oxidative Tandem C–H Aminations and Alkyl Deconstructive Carbofunctionalization

**DOI:** 10.1016/j.isci.2019.04.019

**Published:** 2019-04-19

**Authors:** Taoyuan Liang, He Zhao, Lingzhen Gong, Huanfeng Jiang, Min Zhang

**Affiliations:** 1 Key Lab of Functional Molecular Engineering of Guangdong Province, School of Chemistry and Chemical Engineering, South China University of Technology, Guangzhou, China

**Keywords:** Chemistry, Catalysis, Organic Chemistry

## Abstract

Benzimidazolone constitutes the core structure of numerous pharmaceuticals, agrochemicals, inhibitors, pigments, herbicides, and fine chemicals. Amination of hydrocarbons is an attractive tool for the creation of nitrogen-containing products. However, the multiple steps, harsh conditions, and low atom efficiencies often present in these reactions remain challenging. We present a multicomponent synthesis of functional benzimidazolones from arylamines, dialkylamines, and alcohols, acting via the sequence of copper-catalyzed oxidative tandem C–H aminations and alkyl deconstructive carbofunctionalization. The catalytic transformation forms multiple bonds in one single operation, uses readily available feedstocks and a naturally abundant Cu/O_2_ catalyst system, has broad substrate scope, avoids pre-installation of aminating agents and directing groups, and provides high chemo- and regioselectivity, resulting in direct functionalization of inert C–H and C–C bonds via single-electron oxidation-induced activation mode. This platform can be expected to provide structurally diverse products with interesting biological, chemical, and physical properties.

## Highlights

• Tandem C–H aminations
• C(sp^3^)–C(sp^3^) bond deconstructive carbofunctionalization
• Multicomponent synthesis
• High chemo- and regioselectivity

## Graphical Abstract

**Figure:**
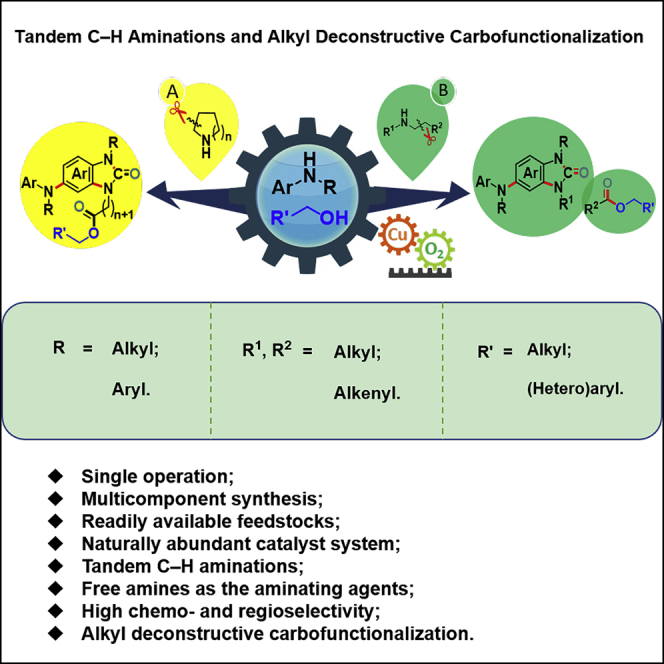

## Introduction

Conventionally, the construction of functional organic products mainly relies on pre-preparation of active reactants followed by noble metal-catalyzed coupling steps, which can easily result in environmental pollution and low utilization efficiency of resources. In this context, there is a high demand for the development of novel catalytic transformations that, via direct functionalization of ubiquitous but poorly reactive C–H and C–C bonds in readily available feedstocks, generate the desired products in the presence of naturally abundant catalyst systems, as such transformations featuring high step and atom efficiency as well as sustainability would pave the ways to address the existing issues.

Among the various functionalizations of hydrocarbons, C–H amination constitutes a particularly attractive tool for the creation of nitrogen-containing products (Park et al., 2017, Kim and Chang, 2017, Beccalli et al., 2017, Boursalian et al., 2016). To date, a number of approaches have been elegantly explored for this purpose (Breslow and Gellman, 1983, Paudyal et al., 2016, Liang et al., 2018; Wertz et al., 2011, Yin et al., 2010, Kim et al., 2010, Gao et al., 2018, Wang et al., 2017a, Wang et al., 2017b, Tang et al., 2018, Wu et al., 2011, Wu et al., 2017, Wang et al., 2016, Wang et al., 2017a, Wang et al., 2017b, Margrey et al., 2017, Romero et al., 2015, Ouyang et al., 2017, Liu et al., 2017, Yang et al., 2017, Zhang et al., 2017). However, some key issues remain to be addressed in this field, such as the need for the pre-installation of specific aminating agents (e.g., nitrenes, N-atom with a leaving substituent, azoles) and directing groups, the use of waste-generating oxidants/additives, and harsh conditions. As such, the search for new C–H amination strategies involving free amines as the aminating agents in the absence of directing groups still remains a highly demanding goal. In terms of carbofunctionalization, much effort has been directed during the past decade toward the difunctionalization of alkenes (Qin et al., 2016, Shen et al., 2013, Shen et al., 2016, Li et al., 2016) and alkynes (Li et al., 2017, Urgoitia et al., 2017, Rubinstein et al., 2014, Rao et al., 2017). Moreover, carbofunctionalization via the cleavage of unsaturated C–C bonds (Sagadevan et al., 2017, Shen et al., 2013, Shen et al., 2016, Qin et al., 2016) has also been nicely established. In comparison, owing to a surrounding environment composed of four bonding atoms, regioselective alkyl deconstructive carbofunctionalization has at present remained a challenging but highly valuable topic in synthetic chemistry, as this process would offer the potential to develop novel transformations producing functional molecules that are difficult to prepare or inaccessible by conventional approaches (Liu et al., 2018). For instance in this regard, the Zhu group has reported a number of transformations on the cleavage of strained alkyl chains such as cyclobutanols (Ren et al., 2015, Ren et al., 2016, Yu et al., 2016, Zhao et al., 2015). Very recently, Roque et al. have demonstrated interesting examples on deconstructive functionalization of cyclic tertiary amines (Roque et al., 2018a, Roque et al., 2018b). However, to the best of our knowledge, the elaboration of functional molecules, via the strategy combining direct C–H amination with deconstructive carbofunctionalization of unstrained alkyl chain, remains a new subject to be explored.

**Scheme 1:**
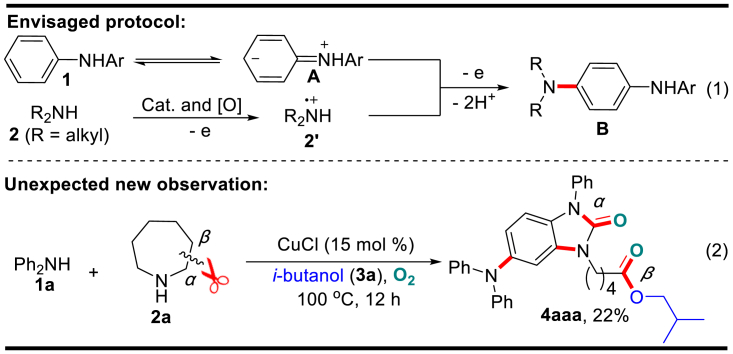
Previous Work and the New Observation

As our sustained effort has been directed toward the functionalization of N-heterocycles (Zhao et al., 2019, Xie et al., 2017, Xie et al., 2018, Xie et al., 2019, Chen et al., 2017), we have recently reported a site-specific fluoroalkylation of aniline derivatives with *in situ*-formed electrophilic radicals (Zhao et al., 2019). This work motivated us to conceive a protocol to aminate the *para*-site of relatively electron-poor diarylamine **1** with electron-rich dialkylamine **2**. As illustrated in Scheme 1, the presence of a suitable catalyst and oxidant is expected to lead to single electron oxidation (SEO) of **2** and generate radical cation **2′**, which then interacts with the zwitterionic form **A** of diarylamine **1** at the sterically less-hindered *para*-site, and generates the amination product **B** via further SEO and deprotonations. However, when we tested the reaction of diphenylamine **1a** and azepane **2a** in *i*-butanol by using CuCl/O_2_ as a catalyst system, we observed that, instead of the anticipated aryl *para*-C–H amination product **B**, a novel functional benzimidazolone **4aaa** was isolated in 22% yield by combining two molecules of **1a**, one molecule of **2a,** and *i*-butanol **3a** (solvent). In such a reaction, three C–N and three C–O bonds are formed in one single operation. Especially, the aryl C–H aminations take place at positions 2 and 4 of diphenylamine **1a**, and the alkyl cleavage in amine **2a** occurs selectively between the *α-* and *β*-sties, which leads to *α*-carboamidation and *β*-carboesterification, respectively.

**Scheme 2:**
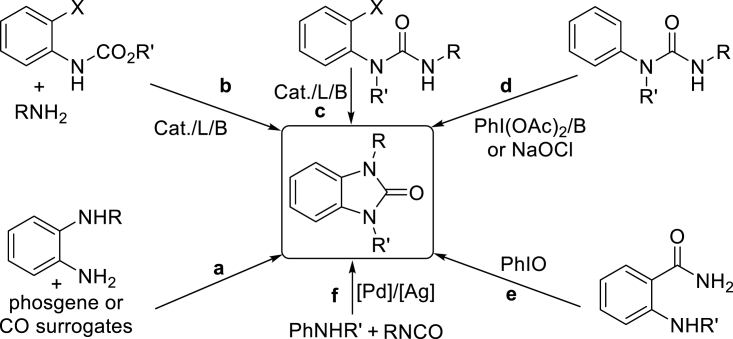
Existing Main Approaches for the Synthesis of Benzimidazolones

It is important to note that benzimidazolone constitutes the core structure of numerous pharmaceuticals, agrochemicals, inhibitors, pigments, herbicides, and fine chemicals (Monforte et al., 2010, Palin et al., 2008, Mastalerz and Oppel, 2012, Mir et al., 2012, Nale and Bhanage, 2015). To date, although there are a number of approaches reported for the synthesis of such compounds, including the cyclization of *o*-phenylenediamine with phosgene or CO surrogates (Scheme 2, path a), (Monforte et al., 2009, Kuethe et al., 2004, Diao et al., 2009) the cyclization of *o*-haloanilines involving C–N bond formation (paths b and c) (Zou et al., 2007, An et al., 2016), the oxidative aryl C–H amidation of N-disubstituted ureas (path d) (Beyer et al., 2001, Li et al., 2008, Yu et al., 2015), PhIO-induced Hofmann rearrangement of amides followed by intramolecular nucleophilic attack by an *ortho*-amino group (path e) (Łukasik and Wróbel, 2016), and the addition of anilines to isocyanates followed by intramolecular oxidative C–H amidation (path f) (Youn and Kim, 2016, Allen and Tidwell, 2013), to the best of our knowledge, the direct construction of benzimidazolones incorporated with additional functionalities from easily available feedstocks is still lacking. On the basis of our new observation, we herein present, for the first time, a multicomponent synthesis of functional benzimidazolones via tandem C–H aminations and alkyl deconstructive carbofunctionalization.

## Results and Discussion

Initially, we focused on screening an efficient catalyst system by choosing the coupling of **1a** and **2a** in *i*-butanol (**3a**) as a model reaction. After evaluation of a series of reaction parameters (Table S1, Supplemental Information), an optimal isolated yield for product **4aaa** was obtained when the reaction charged with an O_2_ balloon was performed at 100°C for 12 h with 20 mol % of CuCl_2_, 2 equiv. of pyridine, and Na_2_CO_3_ (standard conditions), in which Na_2_CO_3_ was used to neutralize the combined HCl in the cyclic amine salts.

**Scheme 3:**
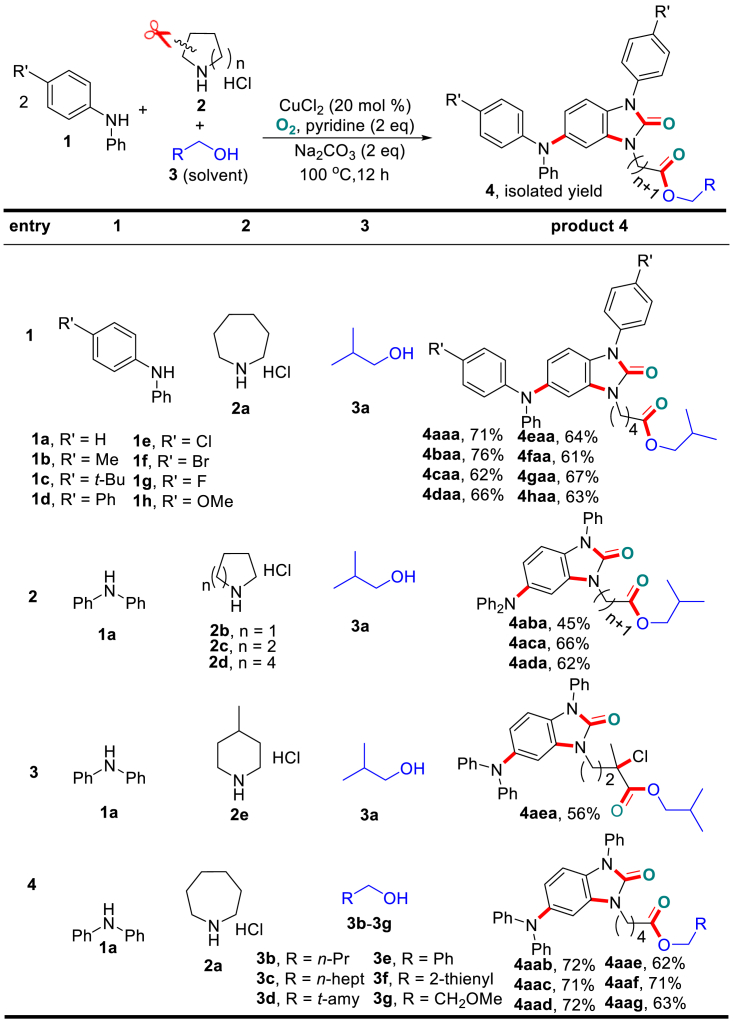
Variation of the Three Coupling Partners Also see Figures S1–S37.

With the optimal conditions established, we then examined the generality of the synthetic protocol. First, various unsymmetrical diarylamines (**1b**-**1h**) in combination with cyclic amines **2a** in *i*-butanol **3a** were explored. As shown in Scheme 3, all the reactions proceeded smoothly and furnished the desired products (**4aaa**-**4haa**) in good isolated yields. The substituents with different electronic properties on the aryl ring of the diarylamines slightly influenced the product yields. Then, we tested the transformation of secondary cyclic amines with different ring sizes (**2b**-**2e**). Similarly all the substrates smoothly coupled with diphenylamine **1a** and *i*-butanol **3a** and provided the N-alkyl products with tunable chain lengths (**4aba**-**4aea**) in moderate to good yields. Interestingly, the use of 4-methylpiperidyl salt **2e** led to the generation of product **4aea,** which involves an additional chlorination at the tertiary *α*-site of the ester group, and the combined HCl in **2e** is believed to serve as the chlorine source. Furthermore, the variation of alcohols had no significant influence on the product formation. Thus different types of alcohols, including linear, branched, (hetero)aryl, and heteroatom-containing alcohols, efficiently reacted with **1a** and **2a** to give the desired products (**4aab**-**4aag**) in good yields. Owing to the excellent compatibility of the different coupling partners, the developed chemistry offers a versatile way for the synthesis of benzimidazolones with structural diversity.

**Scheme 4:**
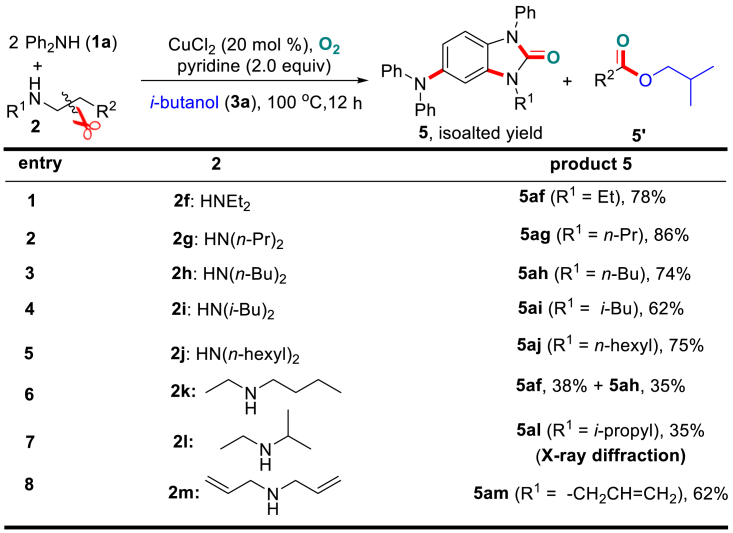
Variation of Open-Chain Dialkylamines Standard condition deviation: without addition of Na_2_CO_3_. Also see Figures S38–S51 and S88 and Tables S2–S6.

The successful transformation of secondary cyclic amines (Scheme 3) subsequently encouraged us to apply the synthetic protocol to the open-chain dialkylamines. As shown in Scheme 4, a series of such substrates (**2f**-**2m**) in combination with diphenylamine **1a** and alcohol **3a** were tested. Gratifyingly, both linear and branched dialkylamines **2** underwent efficient alkyl cleavage between the α- and *β*-carbons, and the *α*-carboamidation generated the N-alkylated benzimidazolone products **5** (i.e., **5af**-**5aj**), whereas the *β*-carboesterofication led to the liberation of the ester by-products **5’**. It is noteworthy that unsymmetrical N-ethylbutan-1-amine (**2k**) generated two products (**5af** and **5ah**) with similar yields, whereas the reaction of N-ethylpropan-2-amine (**2l**) exclusively generated the N-propyl product **5al** with a 35% yield (as confirmed by single-crystal X-ray diffraction, CCDC: 1508570, for details, see Figure S88 and Tables S2–S6), and the C–C bond cleaved at the sterically less-hindered ethyl group. It is also of interest that diallylamine **2m** generated the product **5am** in 62% yield with the retention of the allylic functionality.

**Scheme 5:**
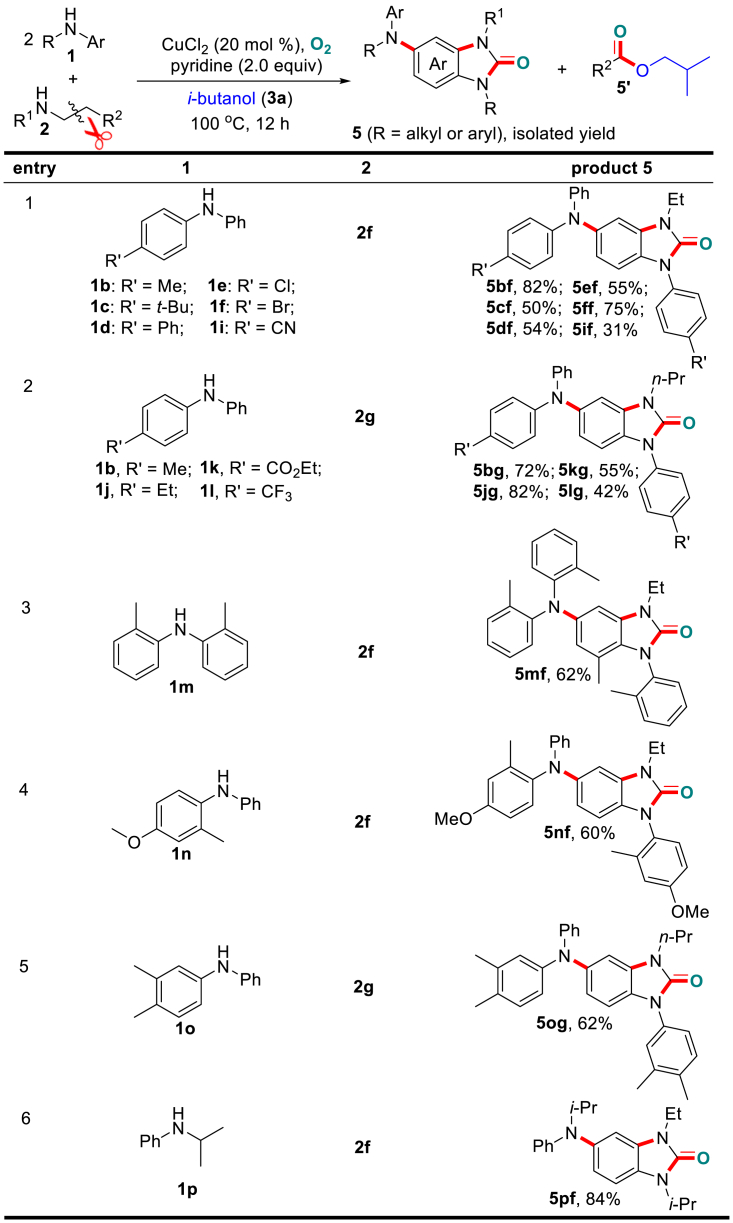
Variation of Both Dialkyl- and Arylamines Standard condition deviation: without addition of Na_2_CO_3_. Also see Figures S52–S83.

Subsequently, we focused on the variation of both diarylamines and open-chain dialkylamines (Scheme 5). Here, substrates **1** with different functionalities on the aryl ring, including –Me, –Et, –*t*-Bu, –Ph, –F, –Cl, –Br, –CN, –CO_2_Et, –NO_2_, and –CF_3_, were well tolerated and afforded the desired products (**5bf**–**5ff**, **5if**, **5bg**, **5jg**, **5kg**, **5lg**, **5mf**–**5nf**, and **5og**) in moderate to excellent yields. The electronic properties of these substituents significantly influenced the product formation. In particular, the electron-rich diarylamines provided much higher yields (**5bf**, **5bg**, **5jg**, **5mf**, **5og**, and **5pf**) than those of strong electron-withdrawing diarylamines (**5if**, **5kg**, and **5lg**). This phenomenon is explained as the result of electron-rich diarylamines favoring the oxidation process to form active intermediates. In addition to diarylamines, N-alkyl aniline **1p** was also favorable for the transformation and produced the desired product **5pf** in high yield.

**Scheme 6:**
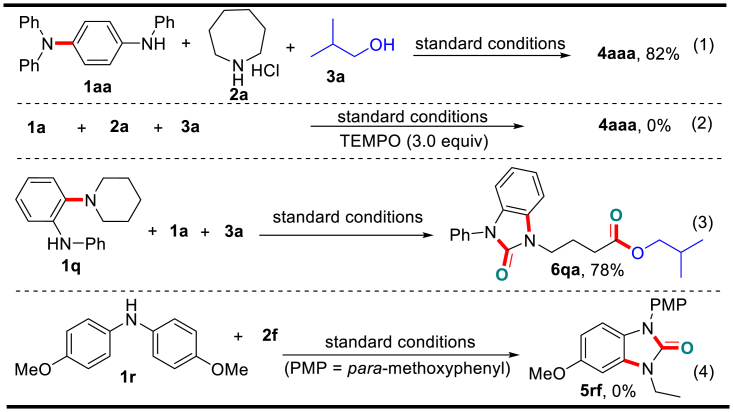
Control Experiments Also see Figures S84–S87.

In an effort to obtain some mechanistic insights into the reaction route, we conducted several control experiments as illustrated in Scheme 6, Equations 1–4 (also see Figures S84–S87). Interruption of the model reaction conducted under standard conditions after 1 h led to the generation of a small concentration of the homo-coupling product **1aa**, which arose from the *para*-C–H amination of diphenylamine **1a**. Thus we employed **1aa** to react with amine **2a** and alcohol **3a** under standard conditions, and benzimidazolone **4aaa** was generated in high yield (Equation 1). This result indicates that **1aa** is a key reaction intermediate. Then the addition of excess 2,2,6,6-tetramethyl-1-piperidinyloxy (TEMPO) to the model reaction completely suppressed the formation of product (Equation 2), indicating that the reaction involves radical intermediates. Furthermore, the reaction of N-phenyl-2-(piperidin-1-yl)aniline **1q** with **1a** and **3a** produced benzimidazolone **6qa** in a 78% yield (Equation 3), where diphenylamine **1a** was not incorporated in the terminal product. However, the *para*-site-blocked diarylamine (**1o**) was unable to couple with amine **2a** to yield product **5rf** (Equation 4). These results indicate that the first aryl *para*-C–H amination of **1a** occurs before the second aryl *ortho*-C–H amination with **2a** and product **4aaa** derives from tandem dual aryl C–H aminations followed by alkyl deconstructive intramolecular *α*-carboamidation and intermolecular *β*-carboesterification.

**Figure 1:**
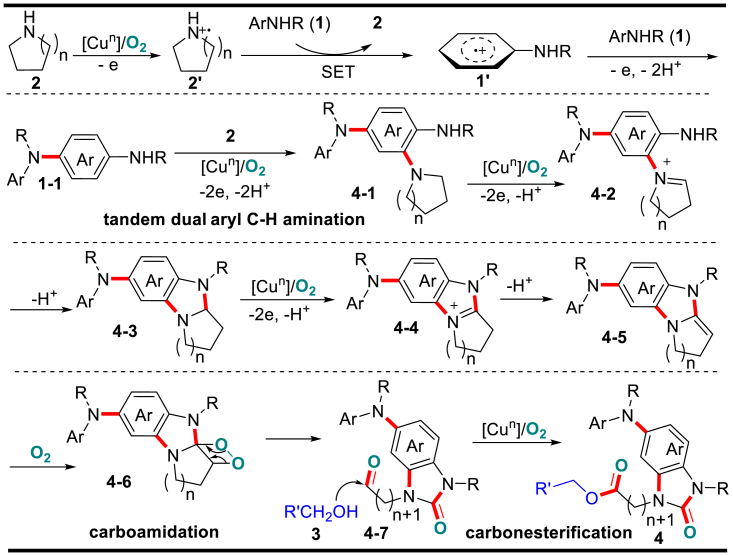
Plausible Reaction Pathway

Based on the above findings, the possible reaction pathway is depicted in Figure 1. Owing to the lower oxidation potential, preferential SEO of dialkylamine **2** (from **2** to **2′**) followed by single-electron transfer from arylamine **1** to the resulting radical cation **2′** would form more stable diarylamino radical cation **1’**. Then, **1′** interacts with another molecule of diarylamine **1** and generates the first aryl *para*-C–H amination product **1-1** via further SEO and deprotonations. Similarly, the aryl radical cation arising from **1-1** interacts with the sterically less hindered dialkylamine **2** at the less congested *ortho*-site and gives rise to the 2,4-diamino intermediate **4-1**. Then, the oxidation of relatively electron-rich alkylamino motif of **4-1** followed by intramolecular nucleophilic addition gives the cyclization adduct **4-3**. Noteworthy, the preferential transformation from dimer **1-1** to **4-1** and **4-3** suppresses the formation of trimeric adducts of diarylamine **1**. Furthermore, the second round oxidation of the alkylamino unit generates iminium ion **4-4** and diamino alkene **4-5,** successively. The O_2_-mediated oxidation of electron-rich C–C double bond in **4-5** would lead to selective C–C bond cleavage (from **4-5** to **4-6**) (Ando et al., 1975) and intramolecular *α*-carboamidation in conjunction with the formation of an aldehyde functionality at the *β*-site. Finally, the oxidative carboesterification of the aldehyde with alcohol **3** would produce product **4**. For the reaction applying open-chain dialkylamine **2**, the C(*α*)–C(*β*) cleavage leads to liberation of the ester by-product.

### Limitations of Study

Anilines and specific cyclic amines such as tetrahydroquinolines and indolines were not applicable in the present reactions and no benzimidazolones products were generated.

### Conclusion

In summary, we have demonstrated, for the first time, a multicomponent synthesis of functional benzimidazolones via tandem C–H aminations and deconstructive carbofunctionalization of unstrained alkyl chain. The catalytic transformation proceeds with the striking features of the formation of three C–N and three C–O bonds in one single operation, the use of readily available feedstocks and a naturally abundant Cu/O_2_ catalyst system, broad substrate scope, no need for pre-installation of specific aminating agents and directing groups, and high chemo- and regioselectivities, which offers an important basis in direct functionalization of inert C–H and C–C bonds via SEO-induced activation mode. The significant utility of benzimidazolones in combination with this novel platform that can be expected to provide structurally diverse products possessing original biological, chemical, and physical properties will incite extensive interest in the scientific community.

## Methods

All methods can be found in the accompanying Transparent Methods supplemental file.

## Data and Software Availability

The crystallography data have been deposited at the Cambridge Crystallographic Data Center (CCDC) under accession number CCDC: 1508570 (**5al**) and can be obtained free of charge from www.ccdc.cam.ac.uk/getstructures.

## Supplemental Information

Document S1. Transparent Methods, Figures S1–S88, and Tables S1–S6

Data S1. The Crystallography Data of 5al
