# Supplementary material for: Synthesis of Multisubstituted Benzimidazolones via Copper-Catalyzed Oxidative Tandem C–H Aminations and Alkyl Deconstructive Carbofunctionalization
Source: iScience. 2019 Apr 19;15:127–35. doi: 10.1016/j.isci.2019.04.019 (PMC6496510; doi:10.1016/j.isci.2019.04.019)
Supplement: Document S1. Transparent Methods, Figures S1–S88, and Tables S1–S6 [file mmc1.pdf]

ISCI, Volume *15*

Supplemental Information

Synthesis of Multisubstituted Benzimidazolones via Copper-Catalyzed Oxidative Tandem C–H Aminations and Alkyl Deconstructive Carbofunctionalization

Taoyuan Liang, He Zhao, Lingzhen Gong, Huanfeng Jiang, and Min Zhang

Copies of product NMR spectra

Figure S1. ^{1}H-NMR (400 MHz, $CDCl_3$) spectrum of **4aaa**, related to **Scheme 3**.

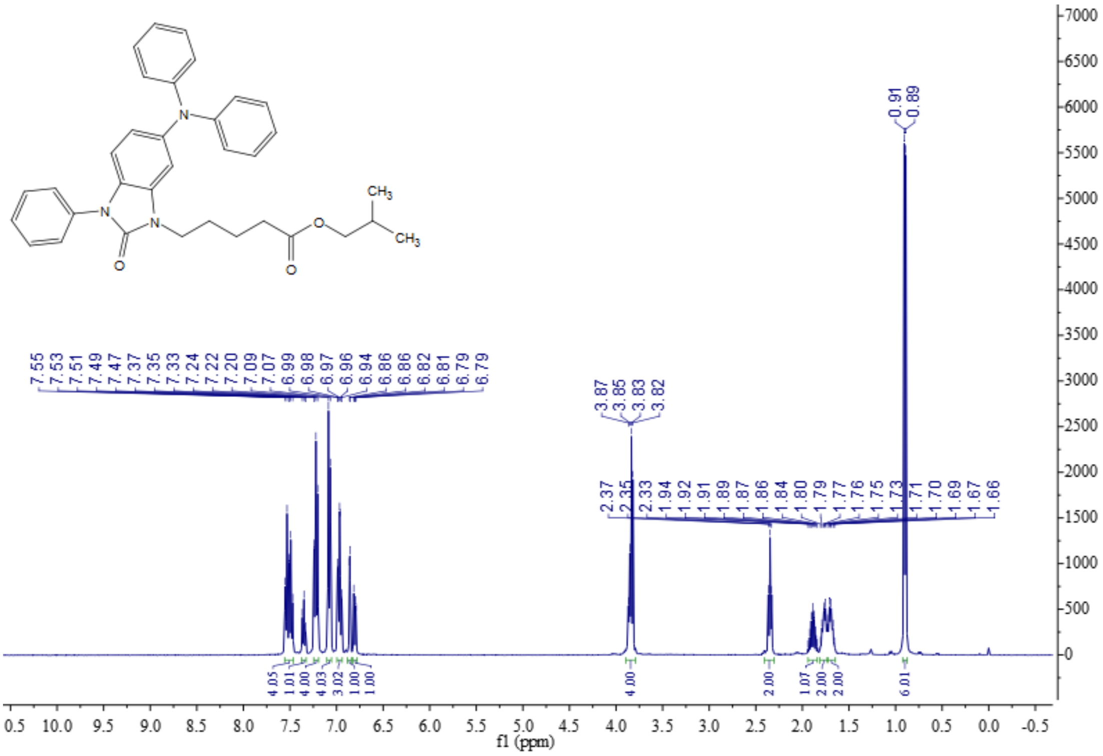

Figure S2. ^{13}C-NMR (100 MHz, $CDCl_3$) spectrum of **4aaa**, related to **Scheme 3**.

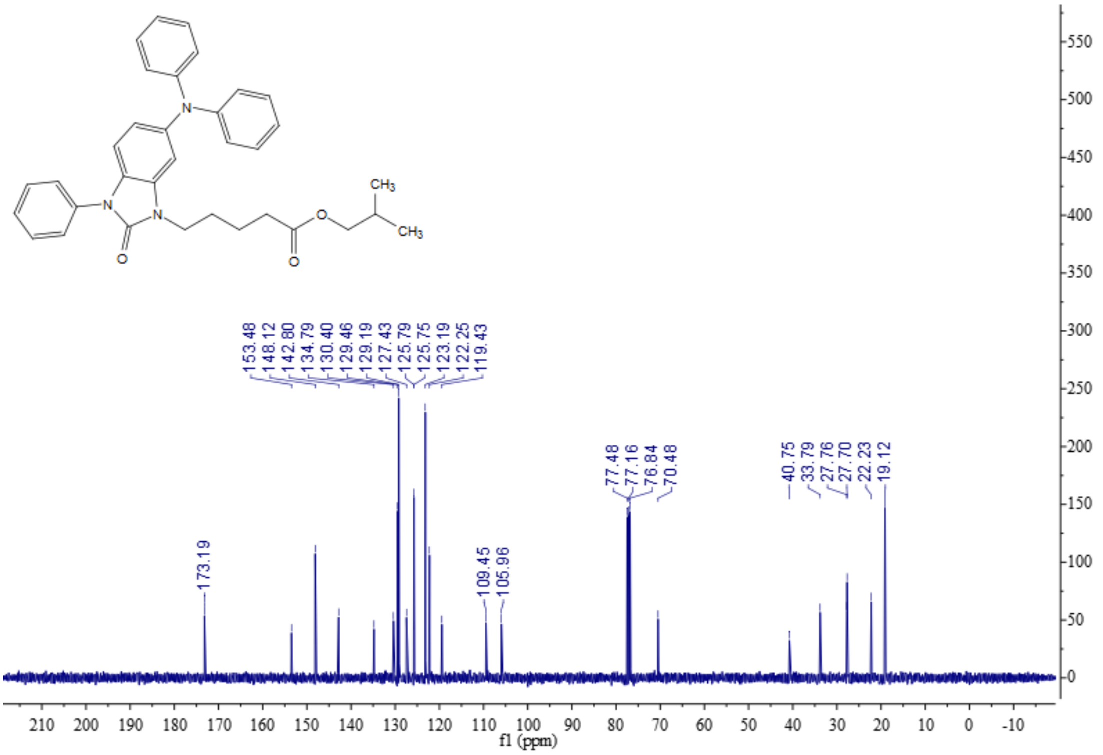

Figure S3. ^{1}H-NMR (400 MHz, $CDCl_3$) spectrum of **4baa**, related to **Scheme 3**.

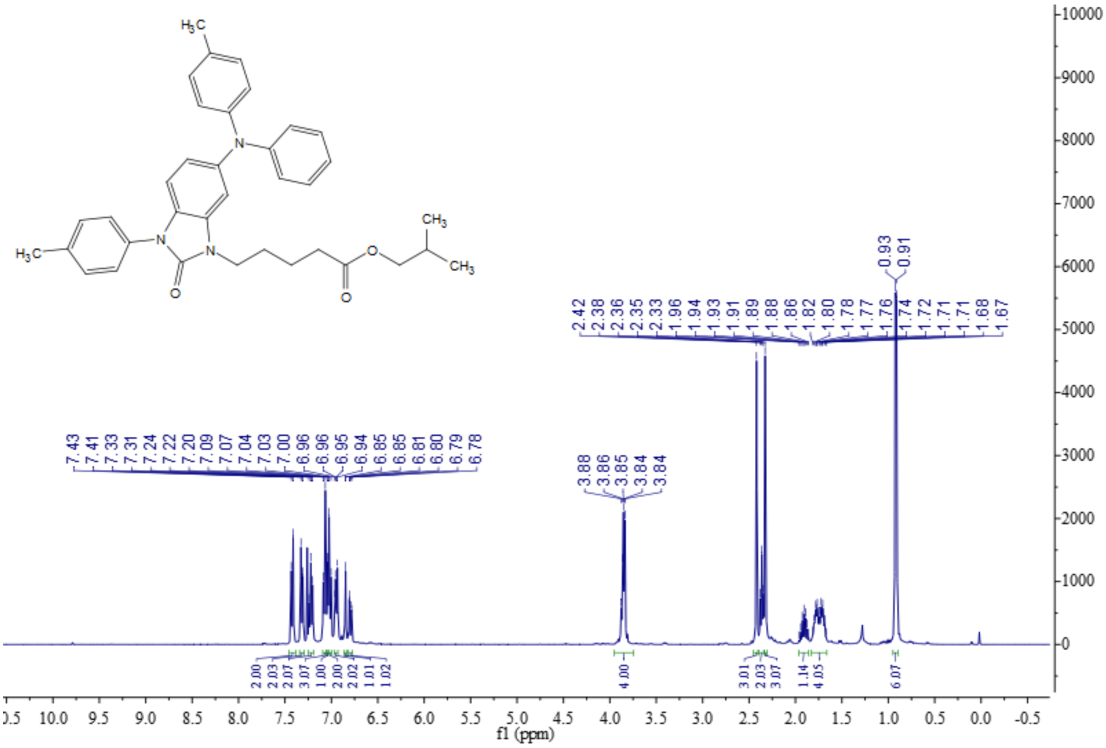

Figure S4. ^{13}C-NMR (100 MHz, $CDCl_3$) spectrum of **4baa**, related to **Scheme 3**.

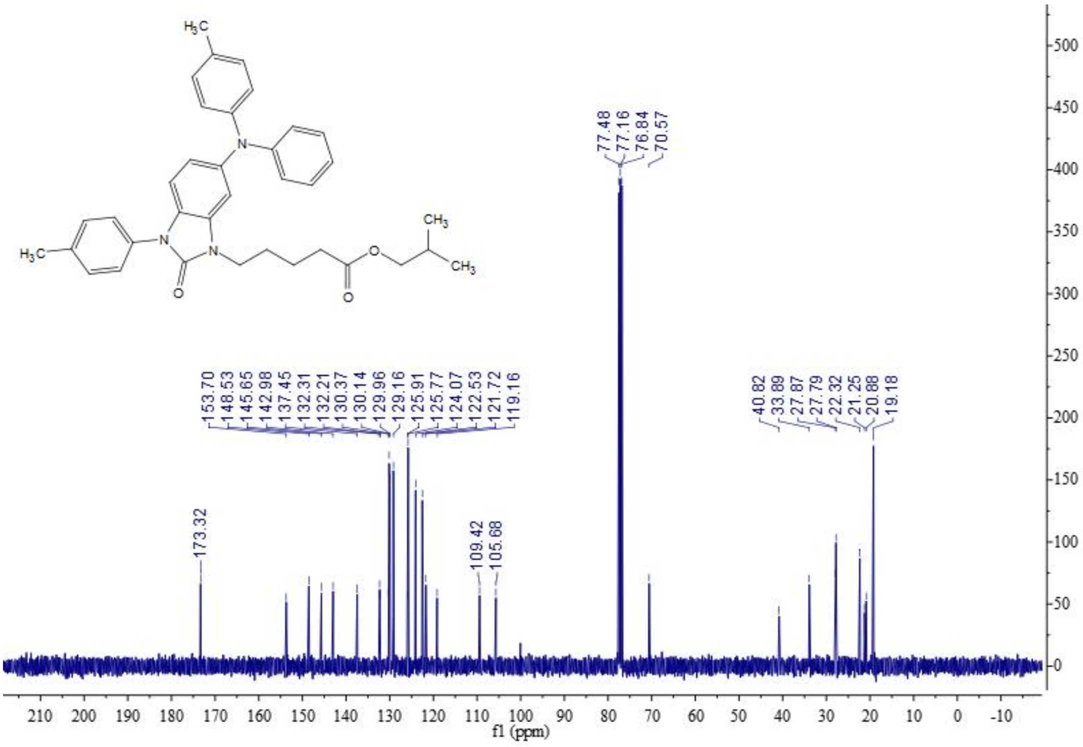

Figure S5. ^{1}H-NMR (400 MHz, $CDCl_3$) spectrum of **4caa**, related to **Scheme 3**.

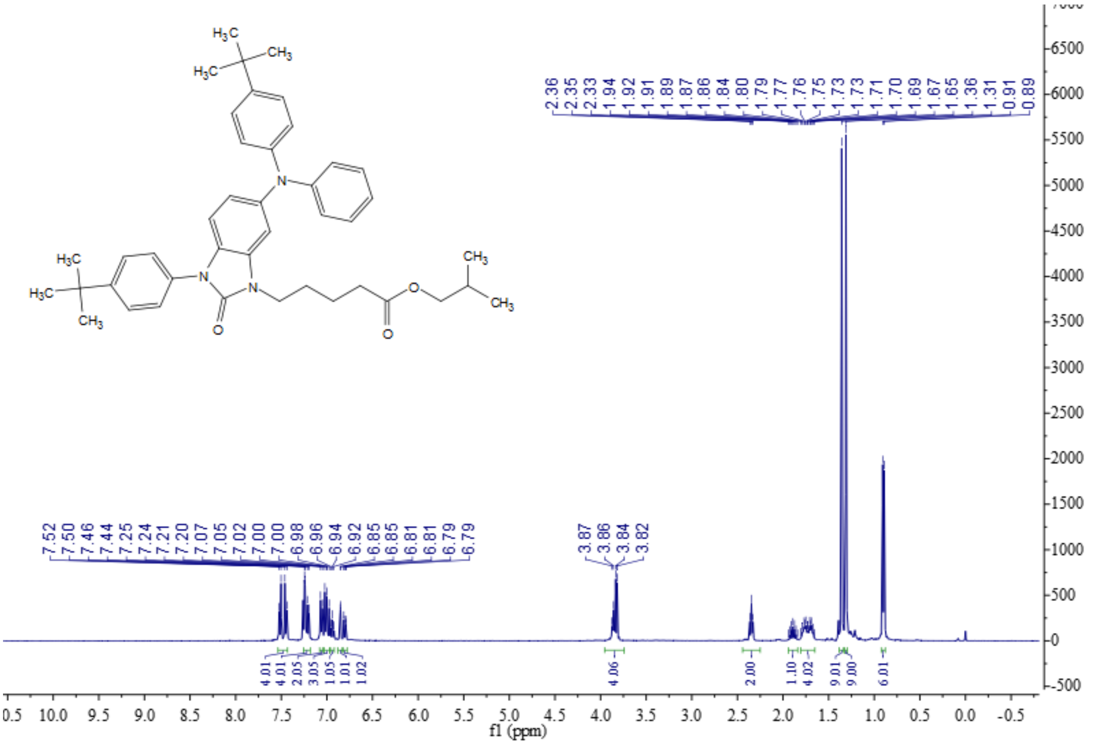

Figure S6. ^{13}C-NMR (100 MHz, $CDCl_3$) spectrum of **4caa**, related to **Scheme 3**.

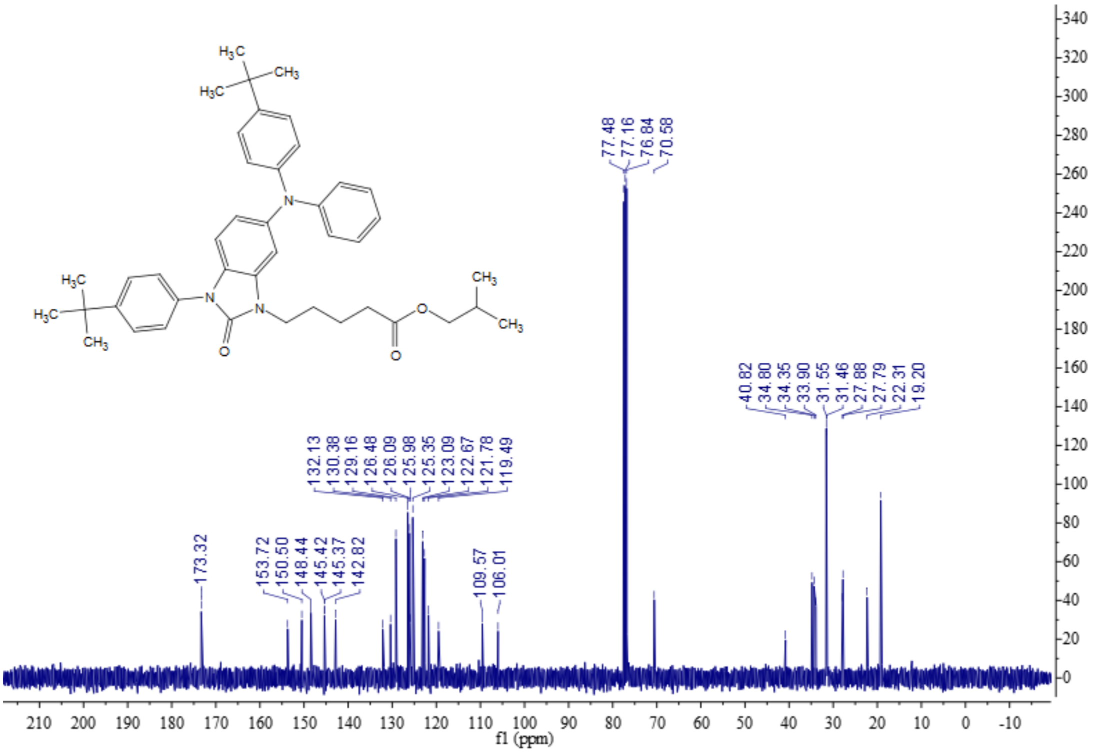

Figure S7. ^{1}H-NMR (400 MHz, $CDCl_3$) spectrum of **4daa**, related to **Scheme 3**.

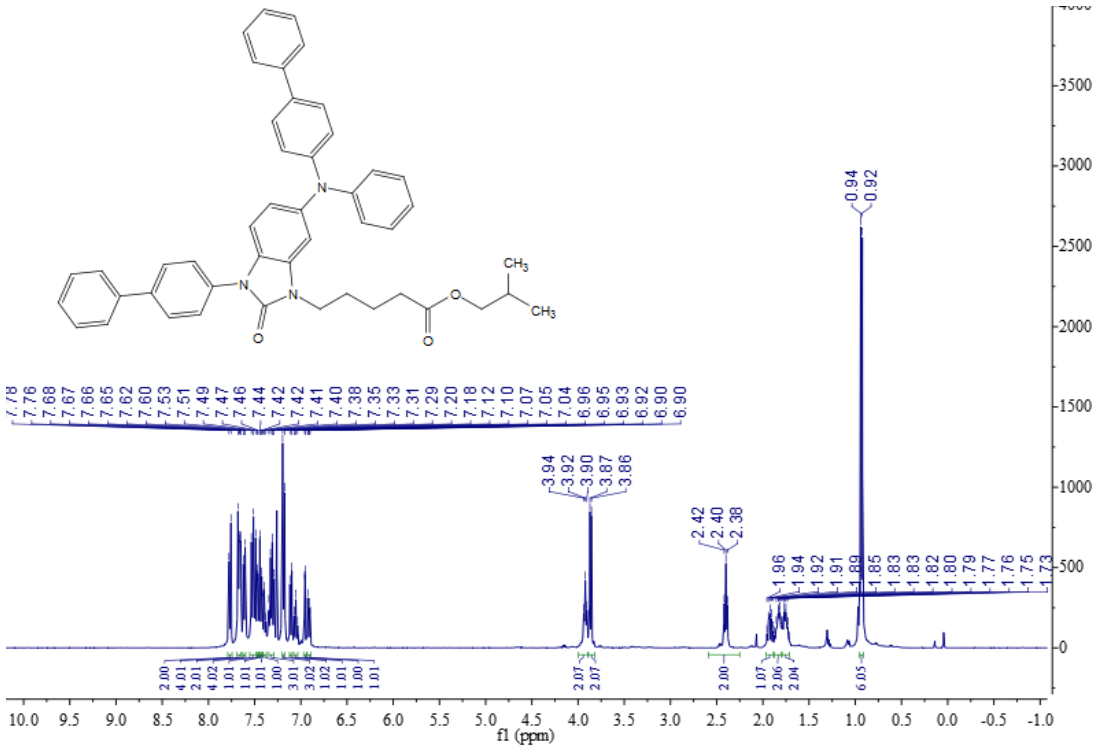

Figure S8. ^{13}C-NMR (100 MHz, $CDCl_3$) spectrum of **4daa**, related to **Scheme 3**.

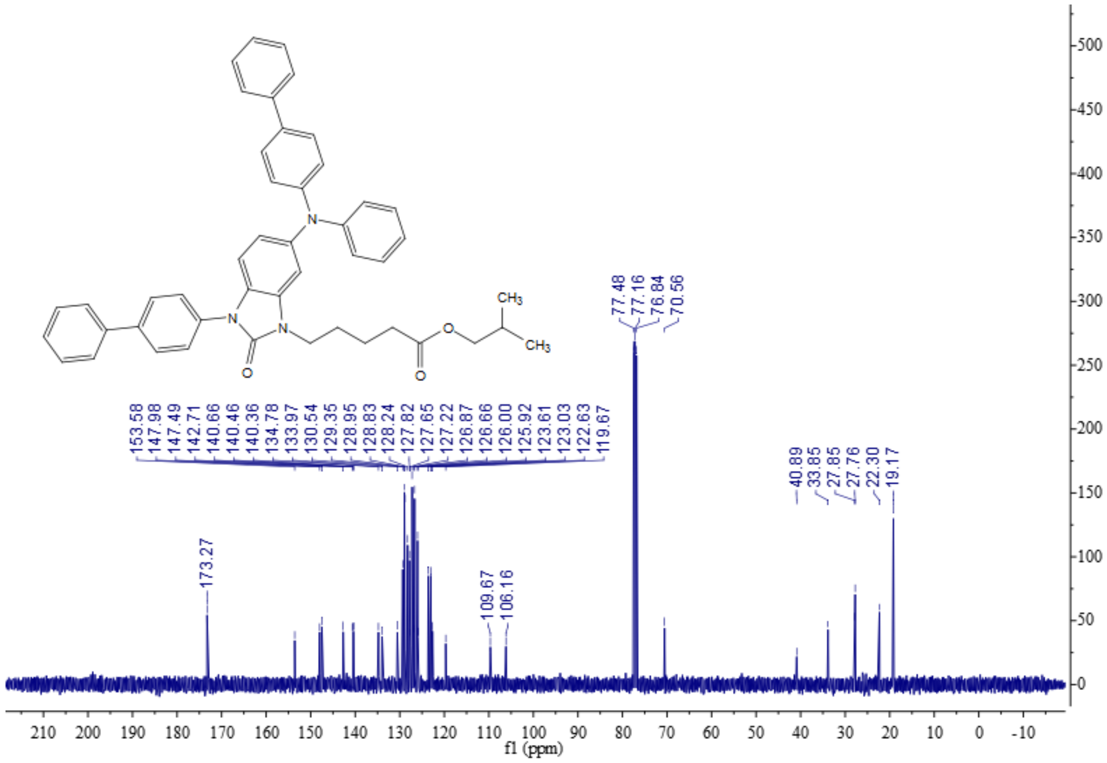

Figure S9. ^{1}H-NMR (400 MHz, $CDCl_3$) spectrum of **4eaa**, related to **Scheme 3**.

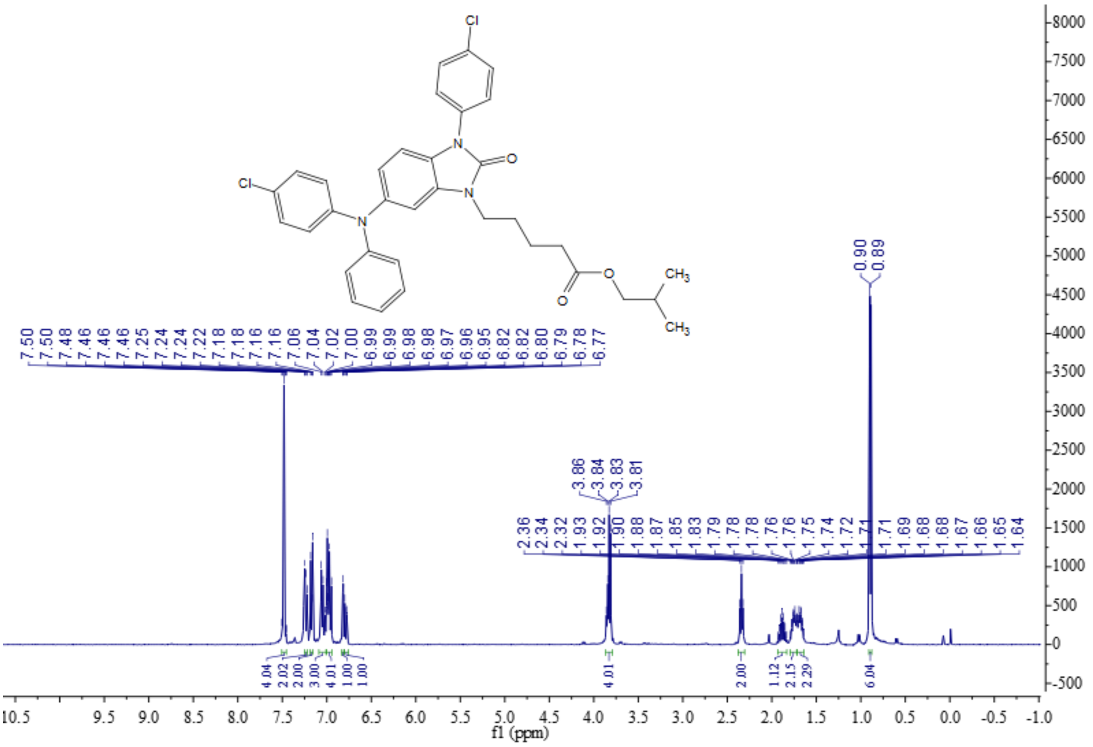

Figure S10. ^{13}C-NMR (100 MHz, $CDCl_3$) spectrum of **4eaa**, related to **Scheme 3**.

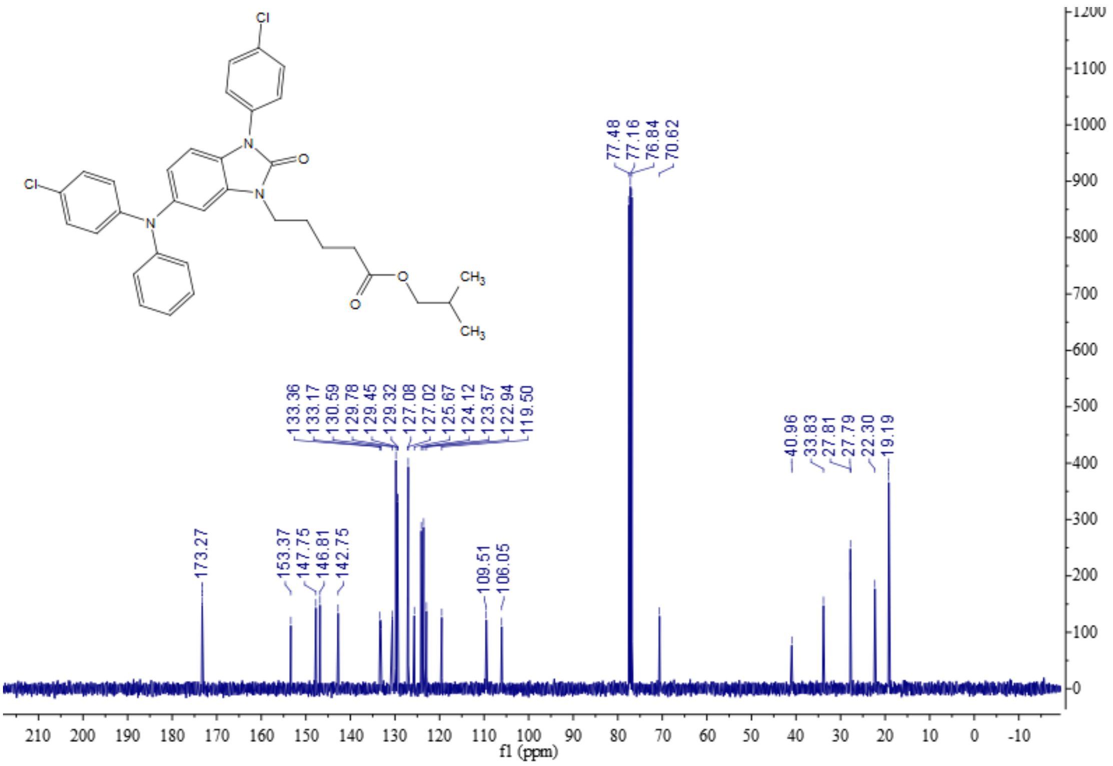

Figure S11. ^{1}H-NMR (400 MHz, $CDCl_3$) spectrum of **4faa**, related to **Scheme 3**.

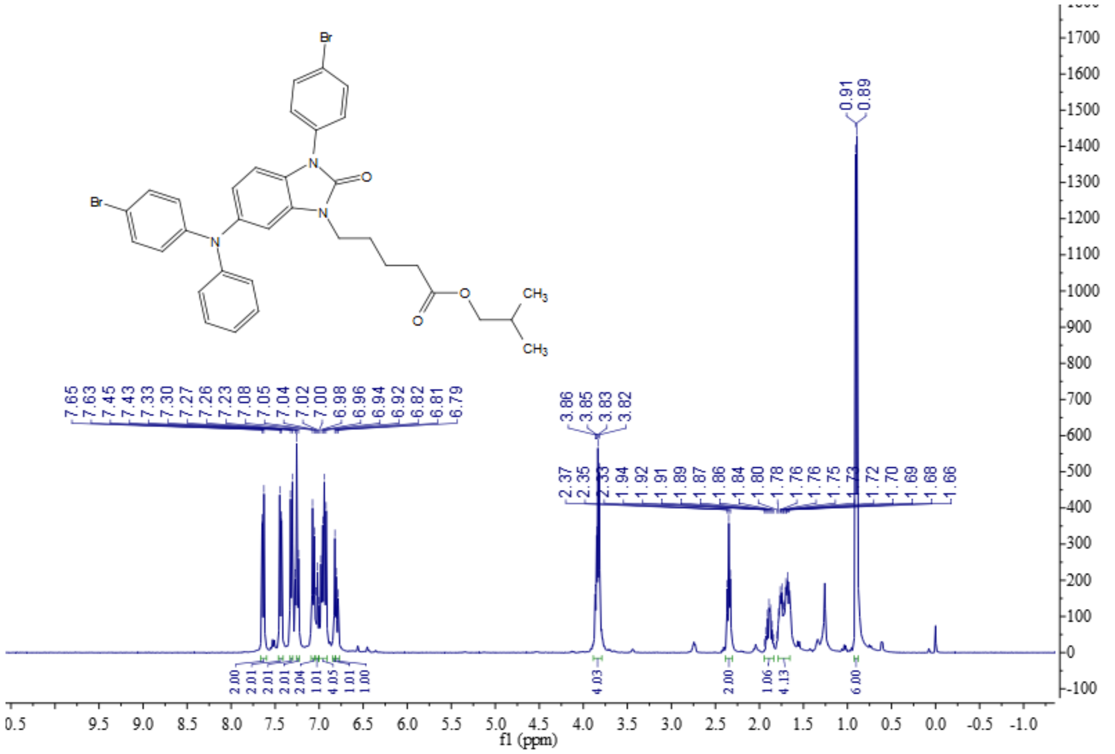

Figure S12. ^{13}C-NMR (100 MHz, $CDCl_3$) spectrum of **4faa**, related to **Scheme 3**.

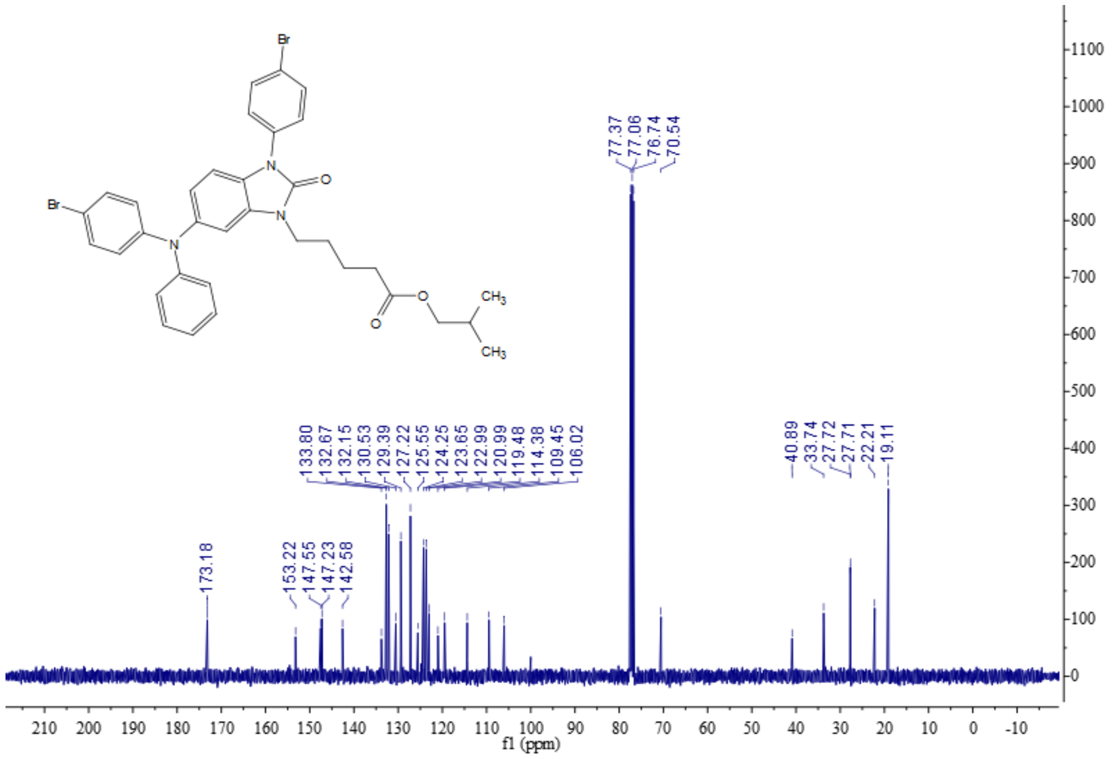

Figure S13. ^{1}H-NMR (400 MHz, $CDCl_3$) spectrum of **4gaa**, related to **Scheme 3**.

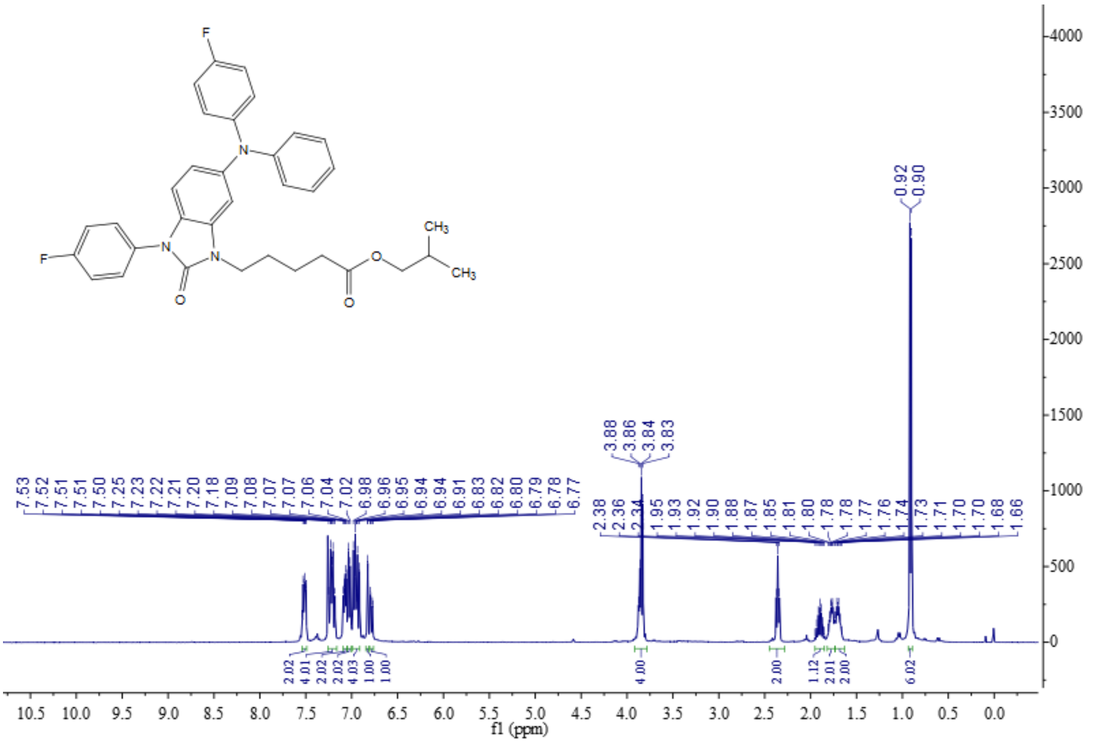

Figure S14. ^{13}C-NMR (100 MHz, $CDCl_3$) spectrum of **4gaa**, related to **Scheme 3**.

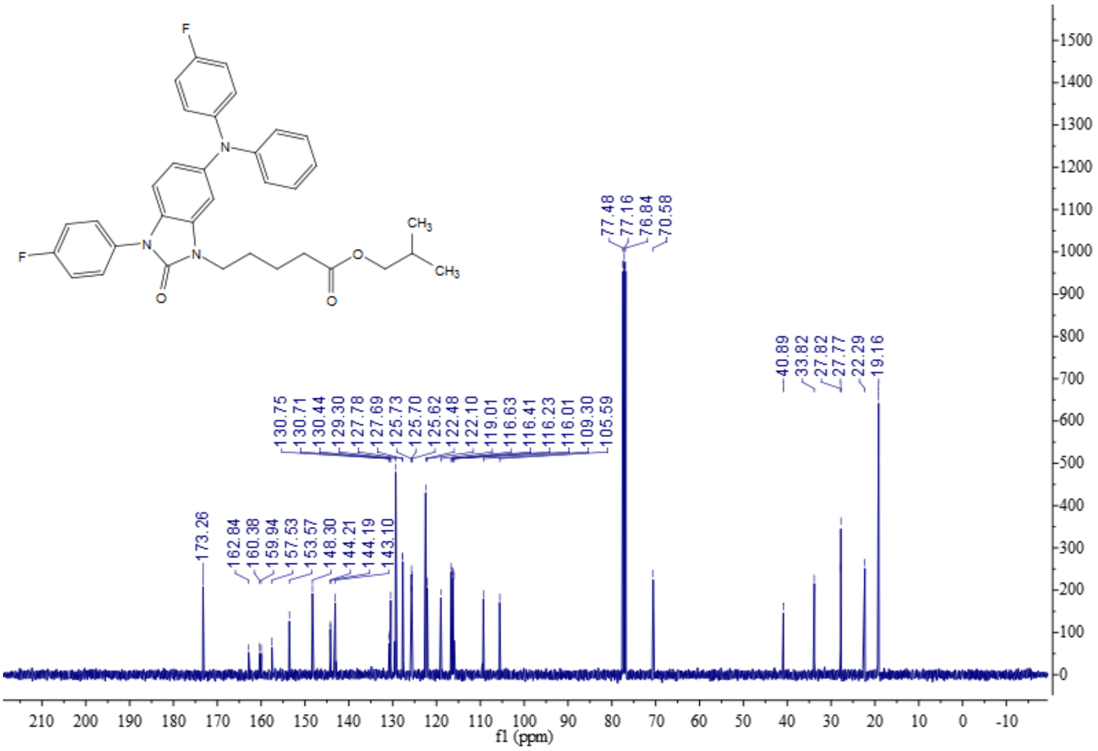

Figure S15. ^{19}F-NMR (376 MHz, $CDCl_3$) spectrum of **4gaa**, related to **Scheme 3**.

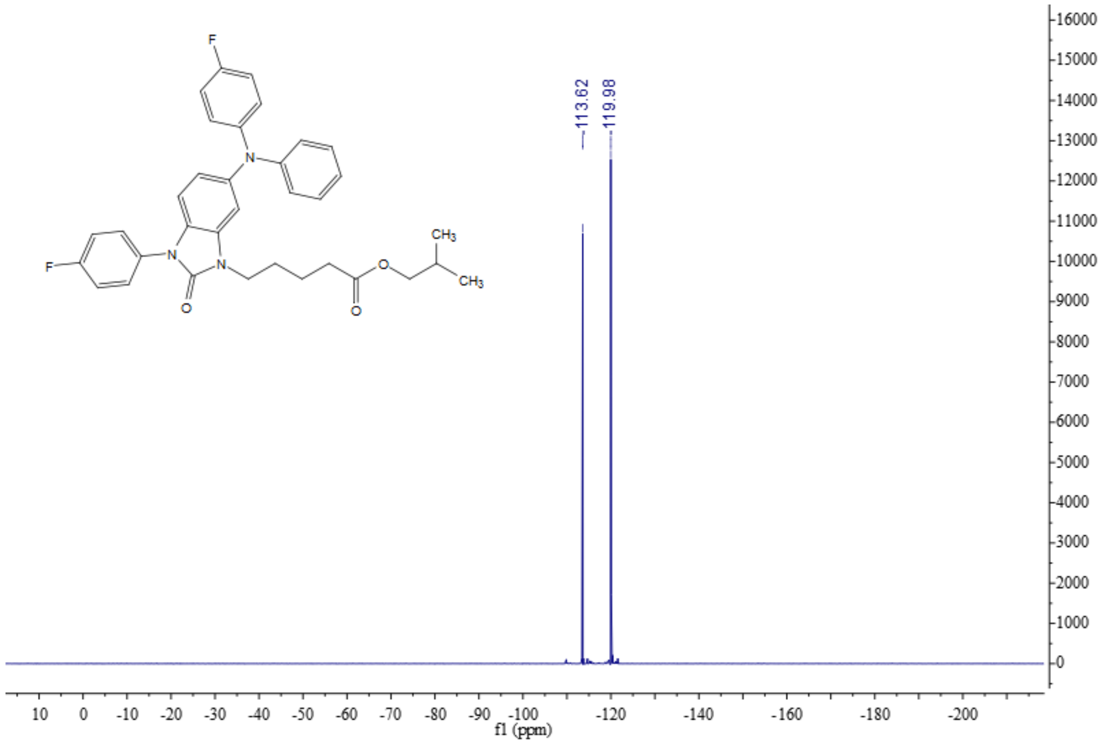

Figure S16. ^{1}H-NMR (400 MHz, $CDCl_3$) spectrum of **4haa**, related to **Scheme 3**.

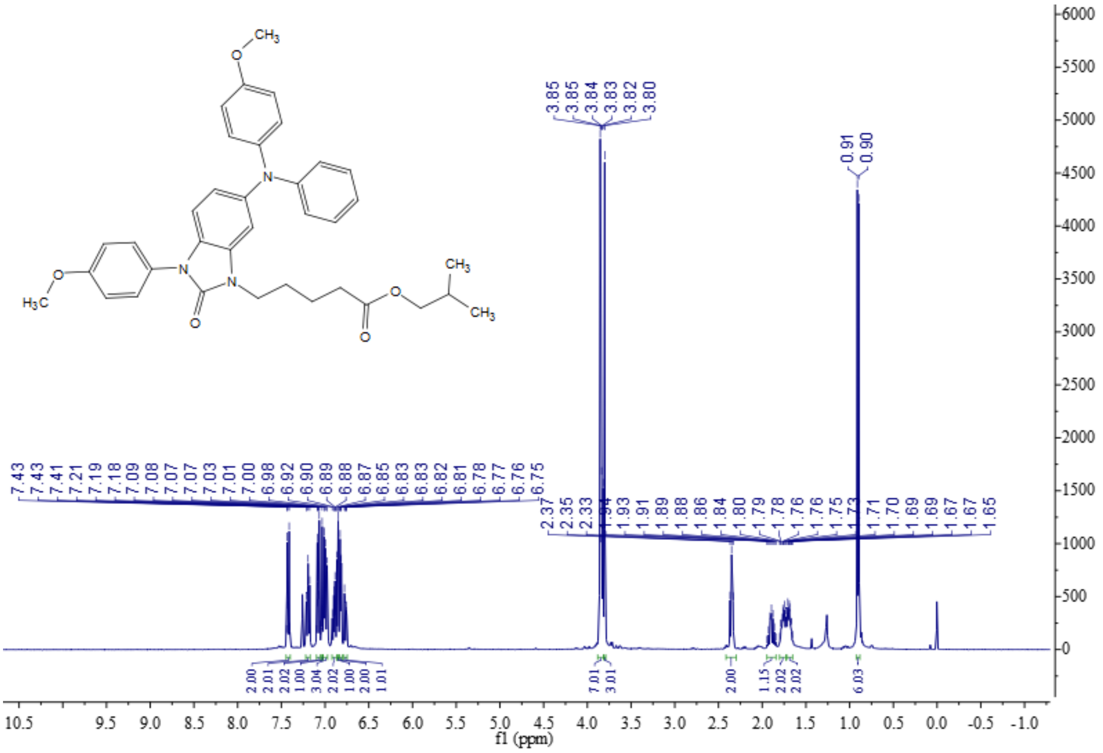

Figure S17. ^{13}C-NMR (100 MHz, $CDCl_3$) spectrum of **4haa**, related to **Scheme 3**.

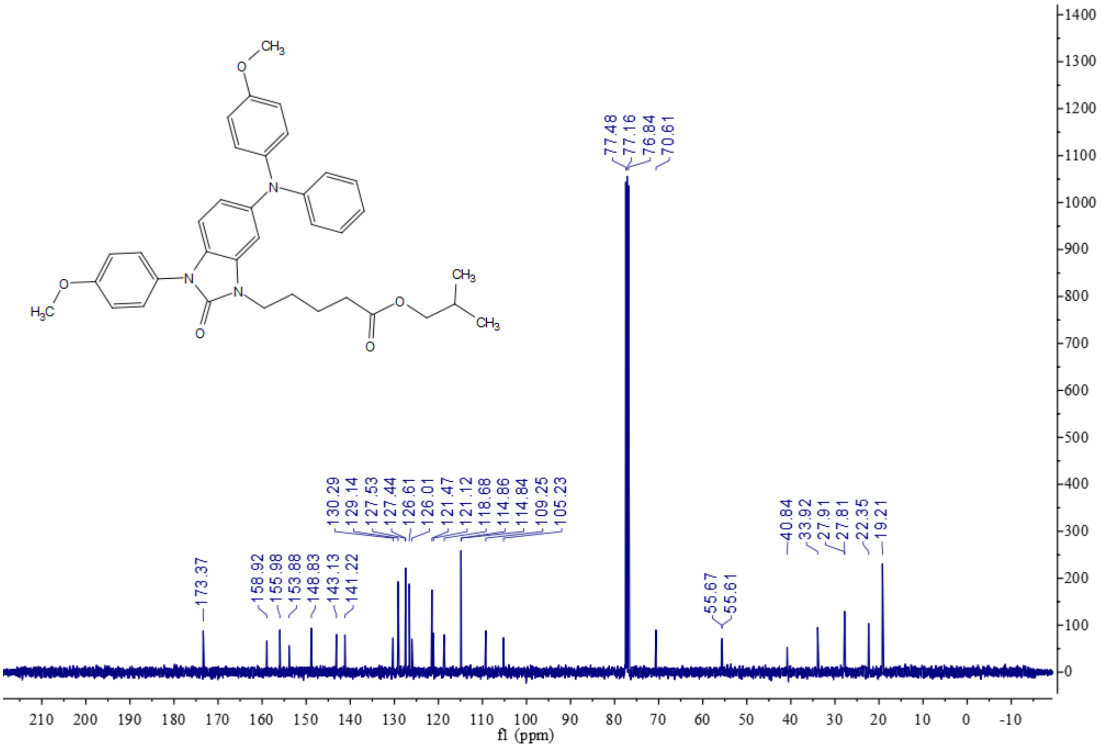

Figure S18. ^{1}H-NMR (400 MHz, $CDCl_3$) spectrum of **4aba**, related to **Scheme 3**.

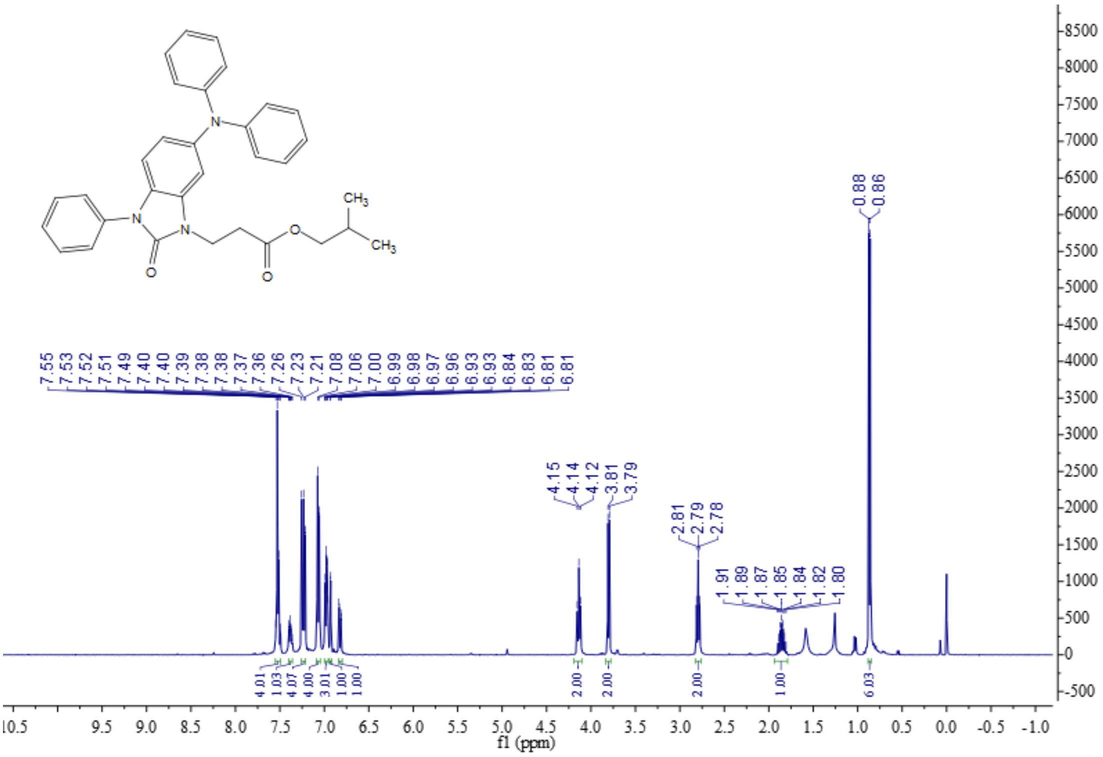

Figure S19. ^{13}C-NMR (100 MHz, $CDCl_3$) spectrum of **4aba**, related to **Scheme 3**.

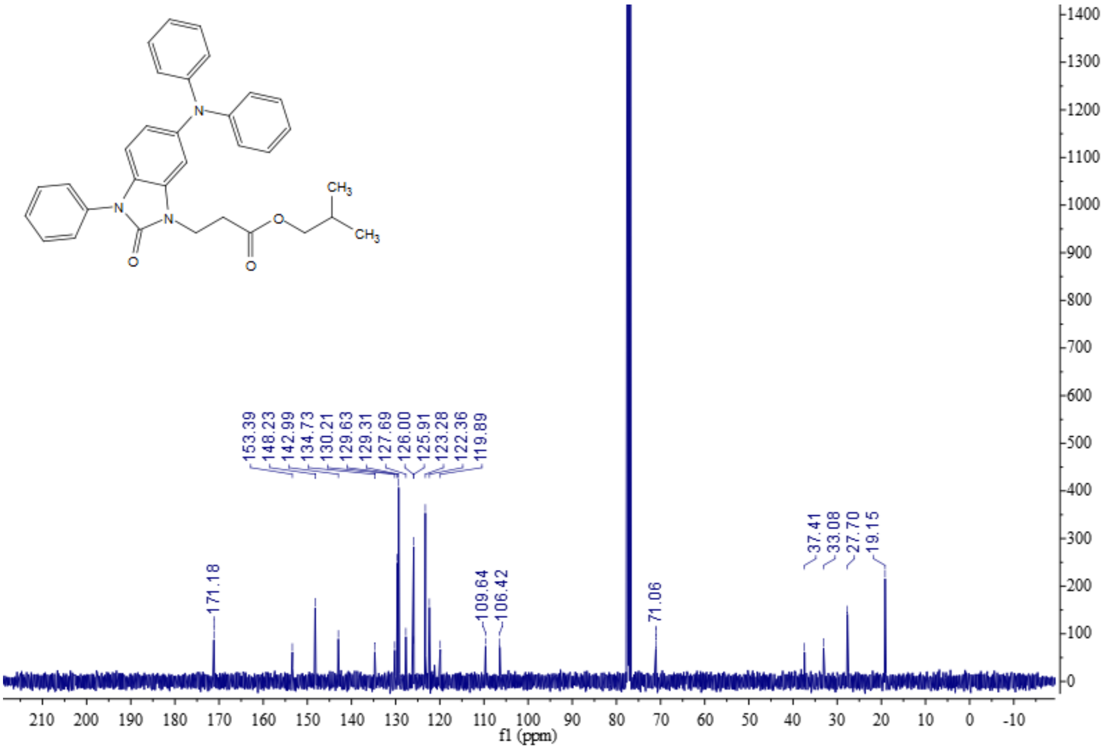

Figure S20. ^{1}H-NMR (400 MHz, $CDCl_3$) spectrum of **4aca**, related to **Scheme 3**.

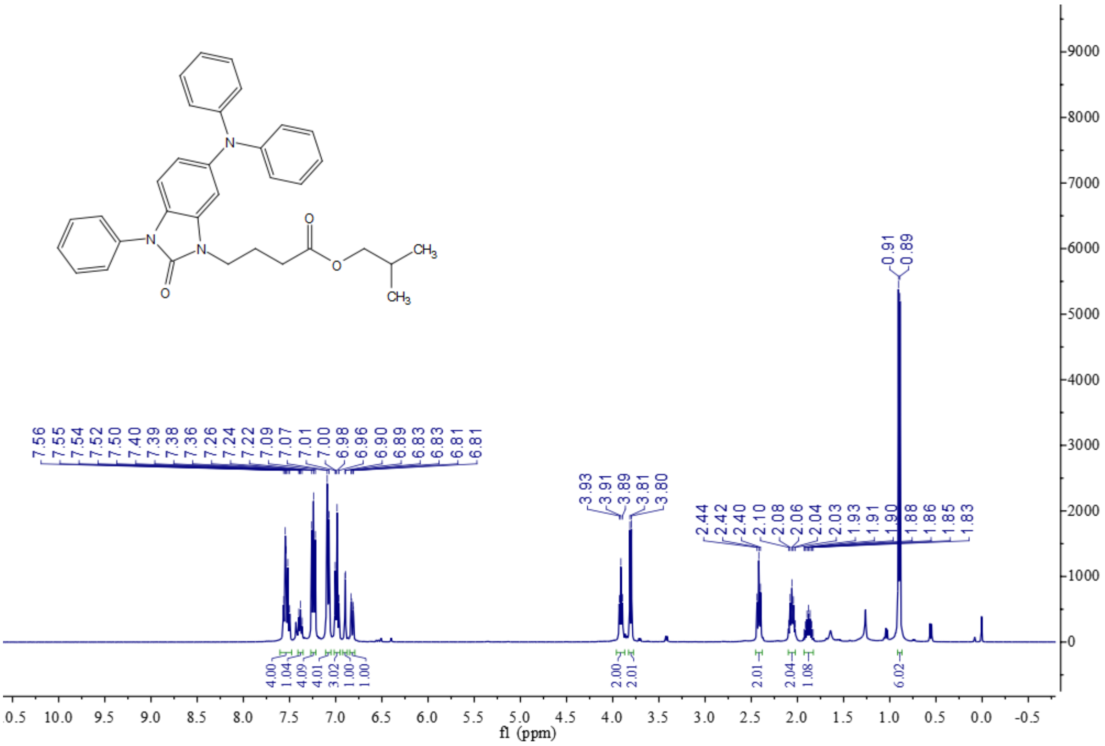

Figure S21. ^{13}C-NMR (100 MHz, $CDCl_3$) spectrum of **4aca**, related to **Scheme 3**.

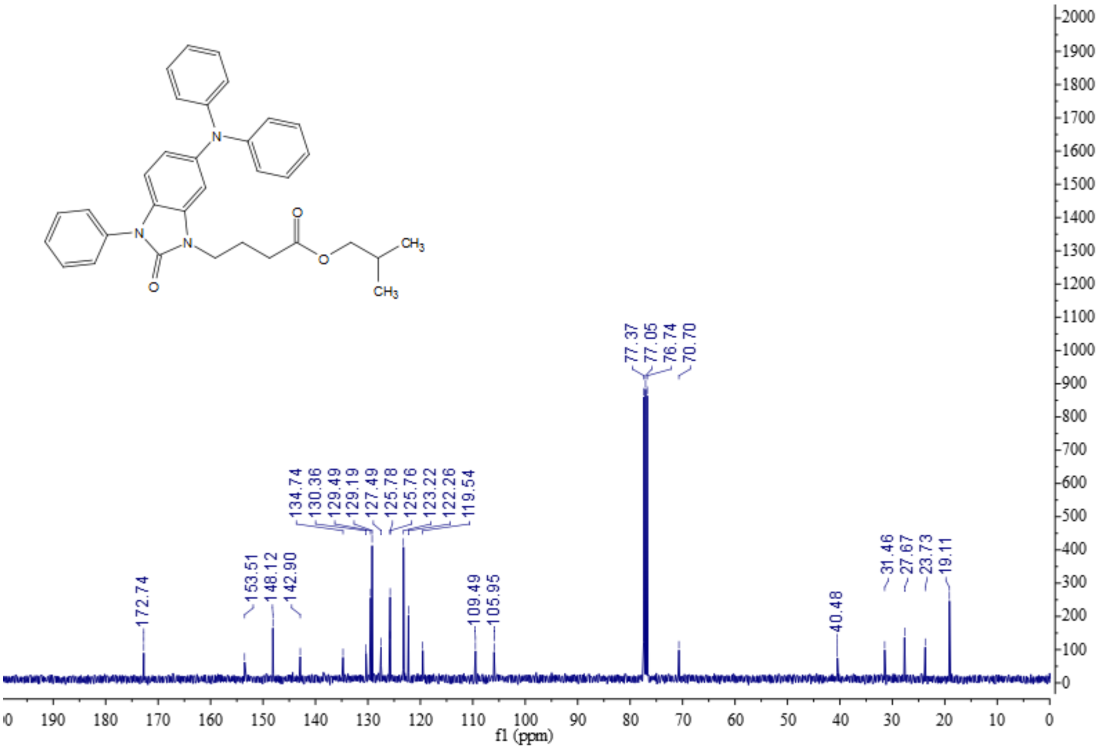

Figure S22. ^{1}H-NMR (400 MHz, $CDCl_3$) spectrum of **4ada**, related to **Scheme 3**.

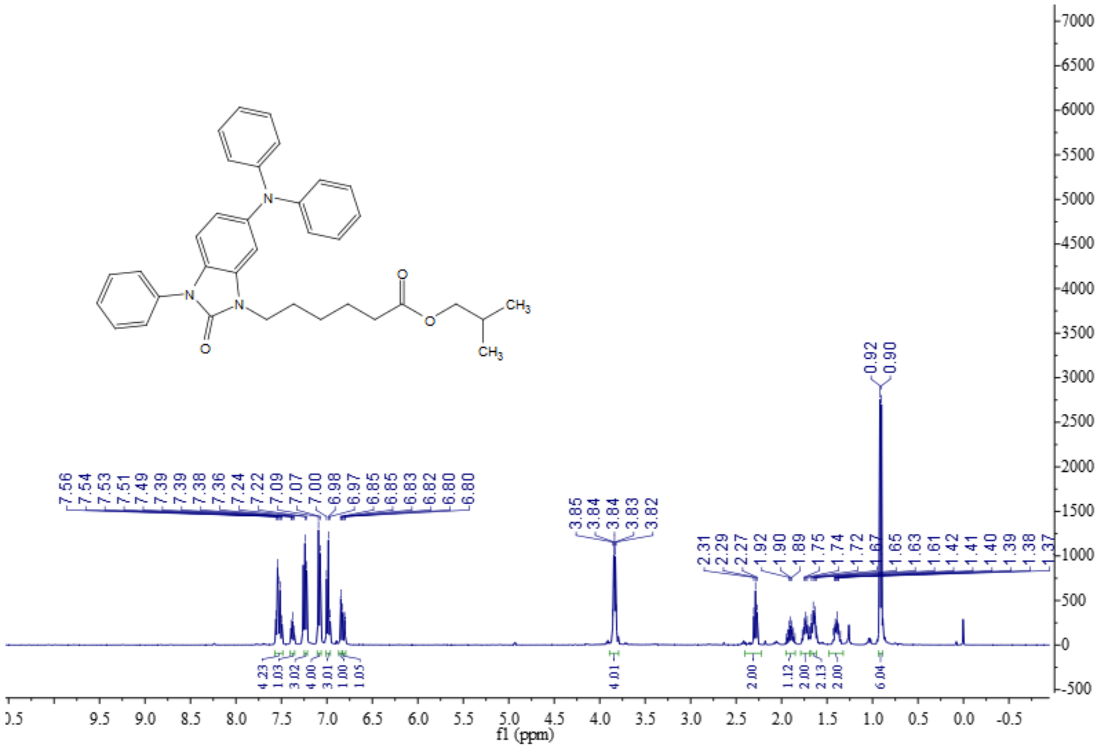

Figure S23. ^{13}C-NMR (100 MHz, $CDCl_3$) spectrum of **4ada**, related to **Scheme 3**.

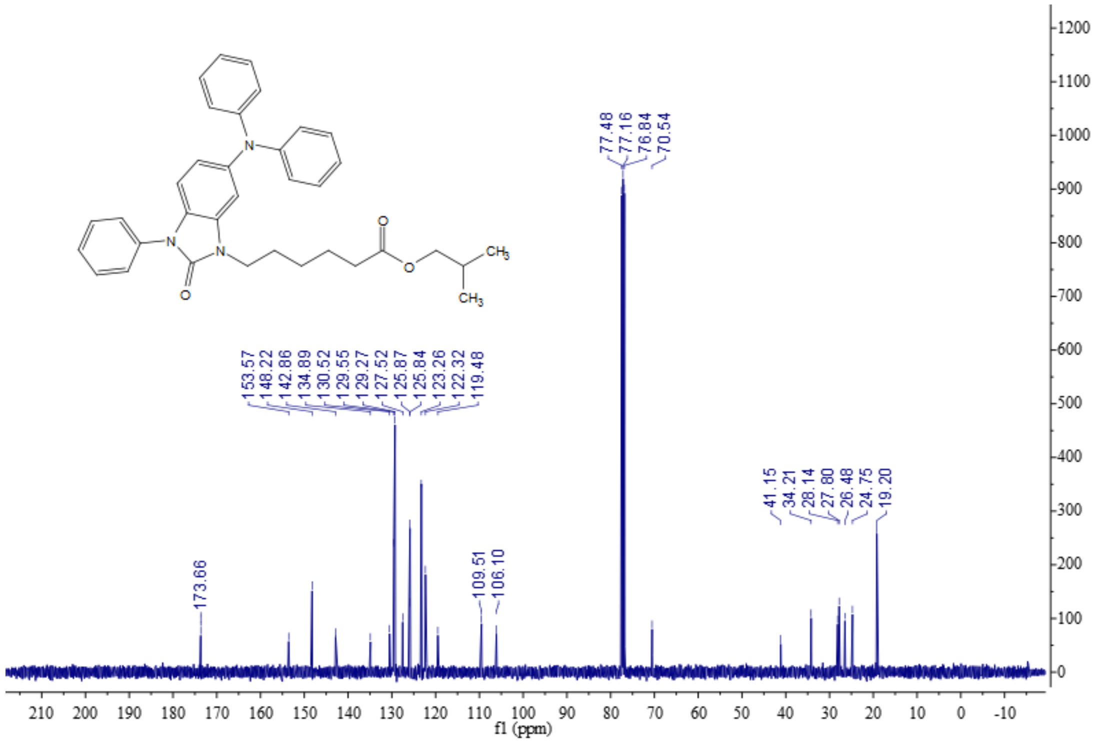

Figure S24. ^{1}H-NMR (400 MHz, $CDCl_3$) spectrum of **4aea**, related to **Scheme 3**.

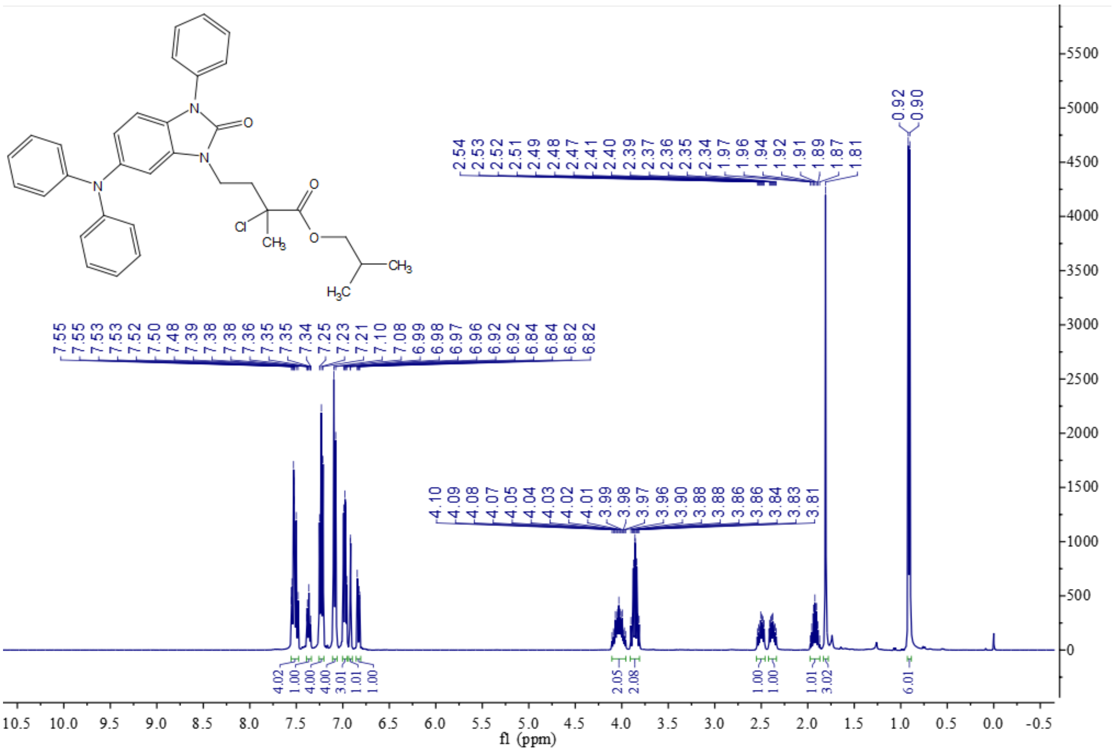

Figure S25. ^{13}C-NMR (100 MHz, $CDCl_3$) spectrum of **4aea**, related to **Scheme 3**.

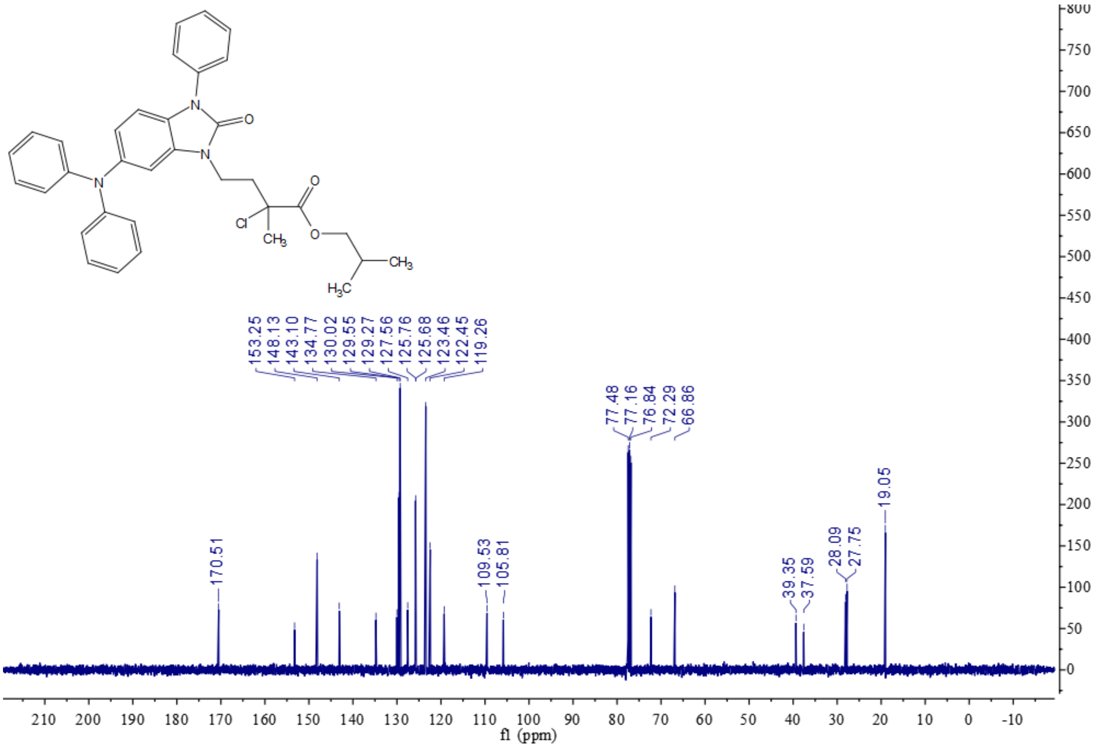

Figure S26. ^{1}H-NMR (400 MHz, $CDCl_3$) spectrum of **4aab**, related to **Scheme 3**.

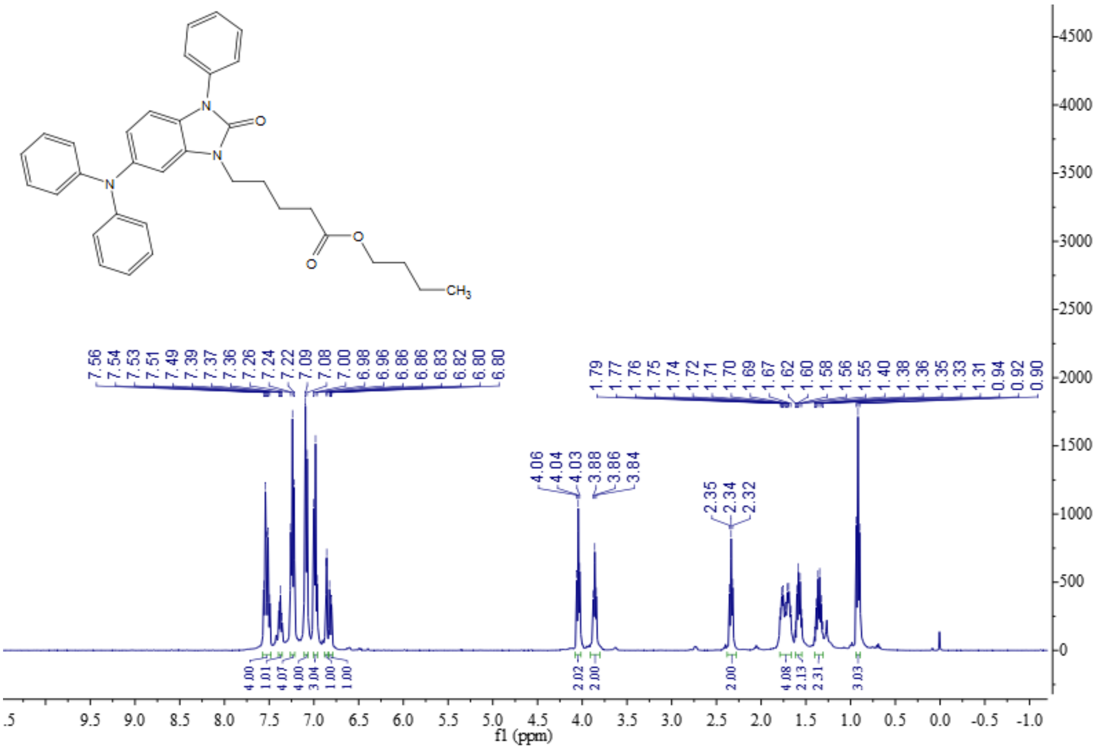

Figure S27. ^{13}C-NMR (100 MHz, $CDCl_3$) spectrum of **4aab**, related to **Scheme 3**.

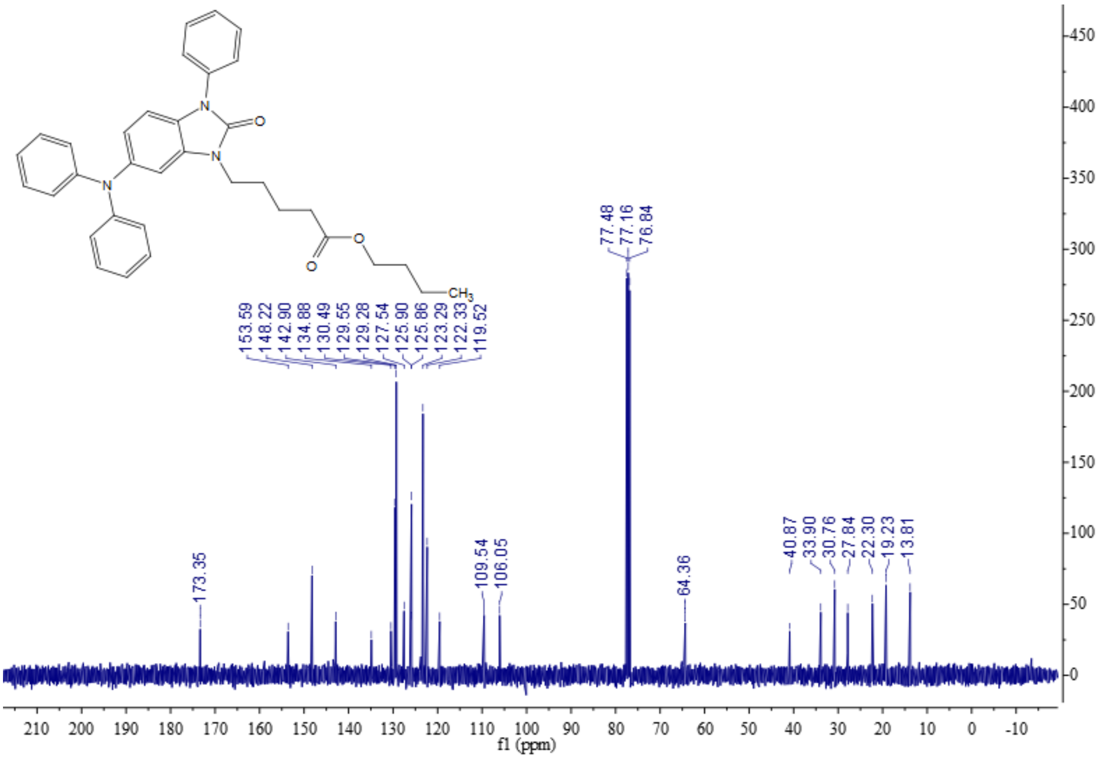

Figure S28. ^{1}H-NMR (400 MHz, $CDCl_3$) spectrum of **4aac**, related to **Scheme 3**.

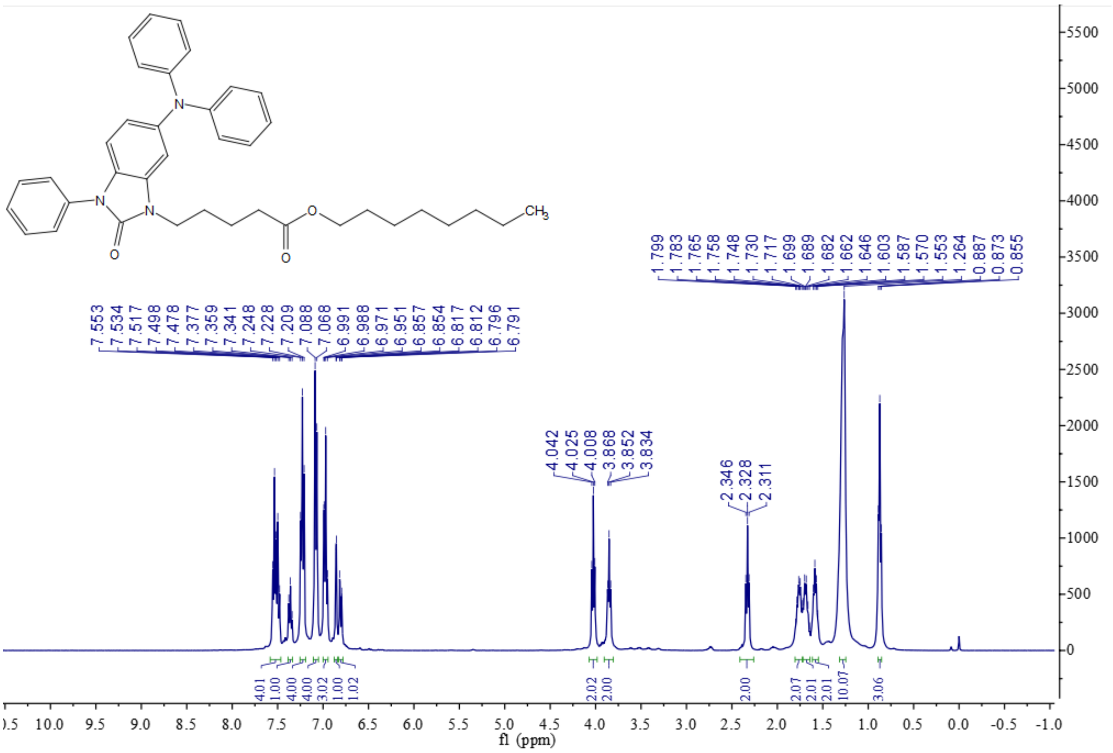

Figure S29. ^{13}C-NMR (100 MHz, $CDCl_3$) spectrum of **4aac**, related to **Scheme 3**.

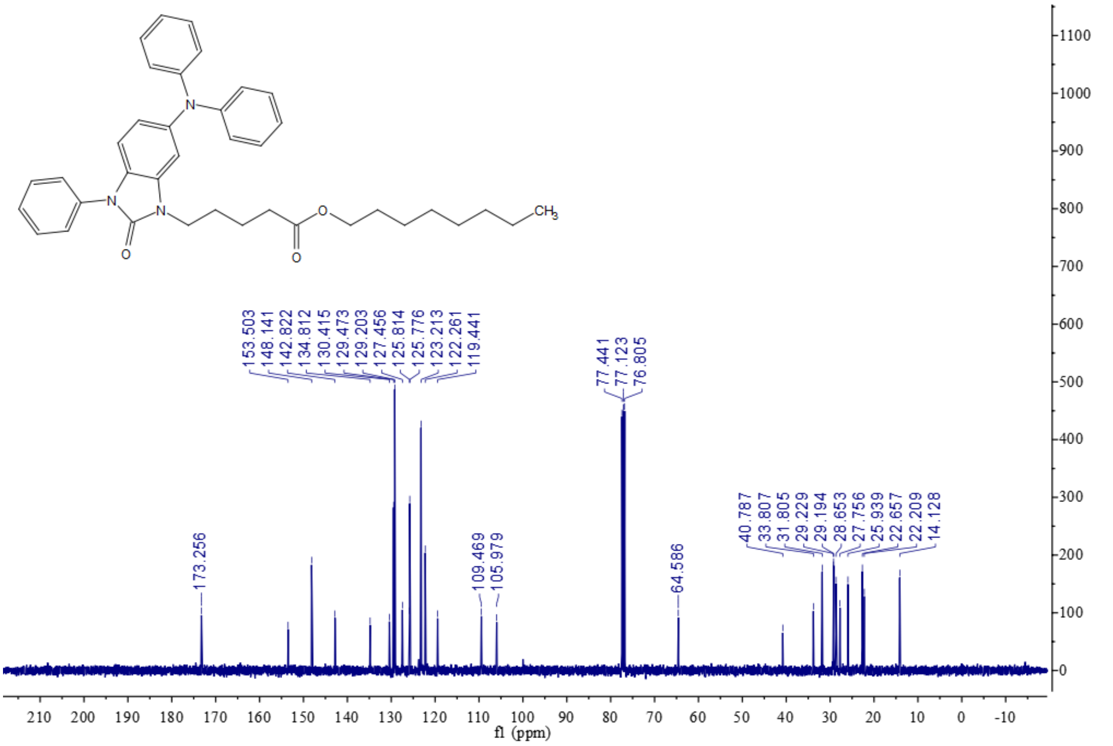

Figure S30. ^{1}H-NMR (400 MHz, $CDCl_3$) spectrum of **4aad**, related to **Scheme 3**.

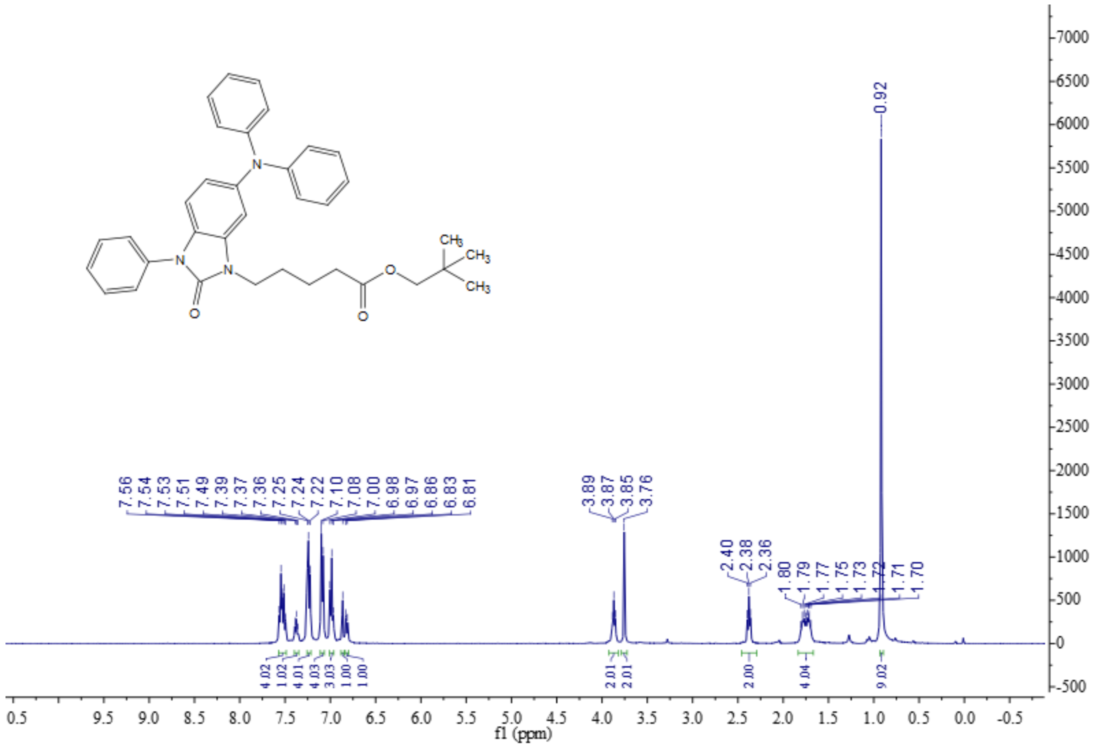

Figure S31. ^{13}C-NMR (100 MHz, $CDCl_3$) spectrum of **4aad**, related to **Scheme 3**.

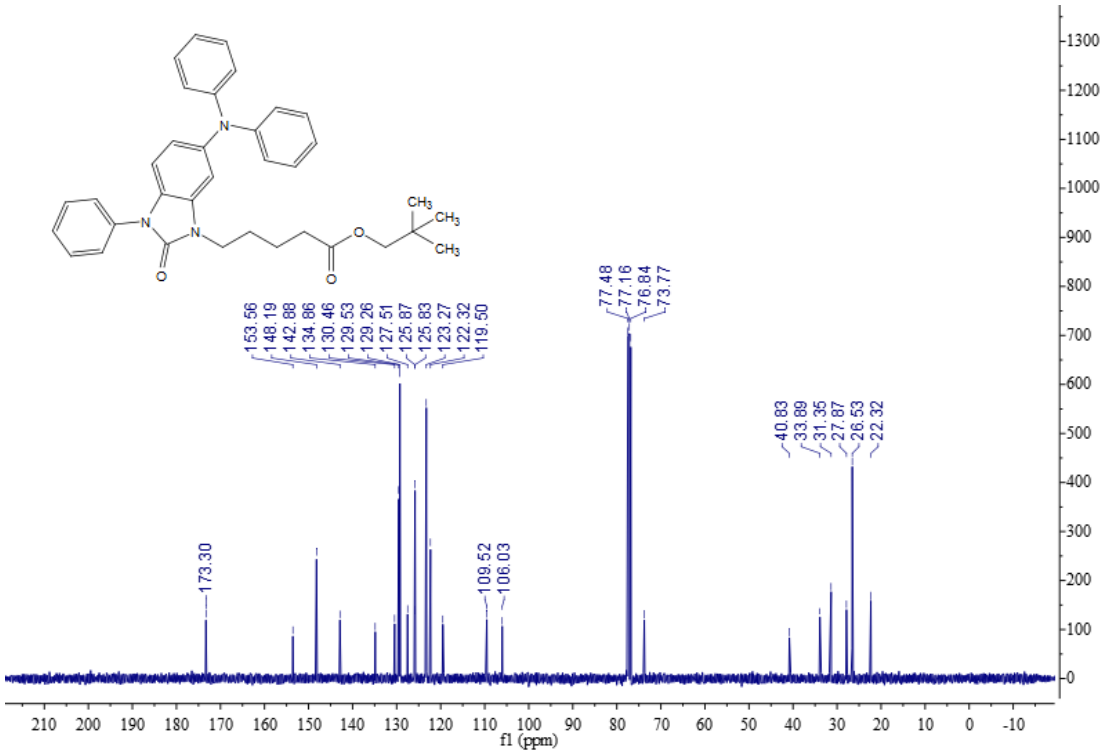

Figure S32. ^{1}H-NMR (400 MHz, $CDCl_3$) spectrum of **4aae**, related to **Scheme 3**.

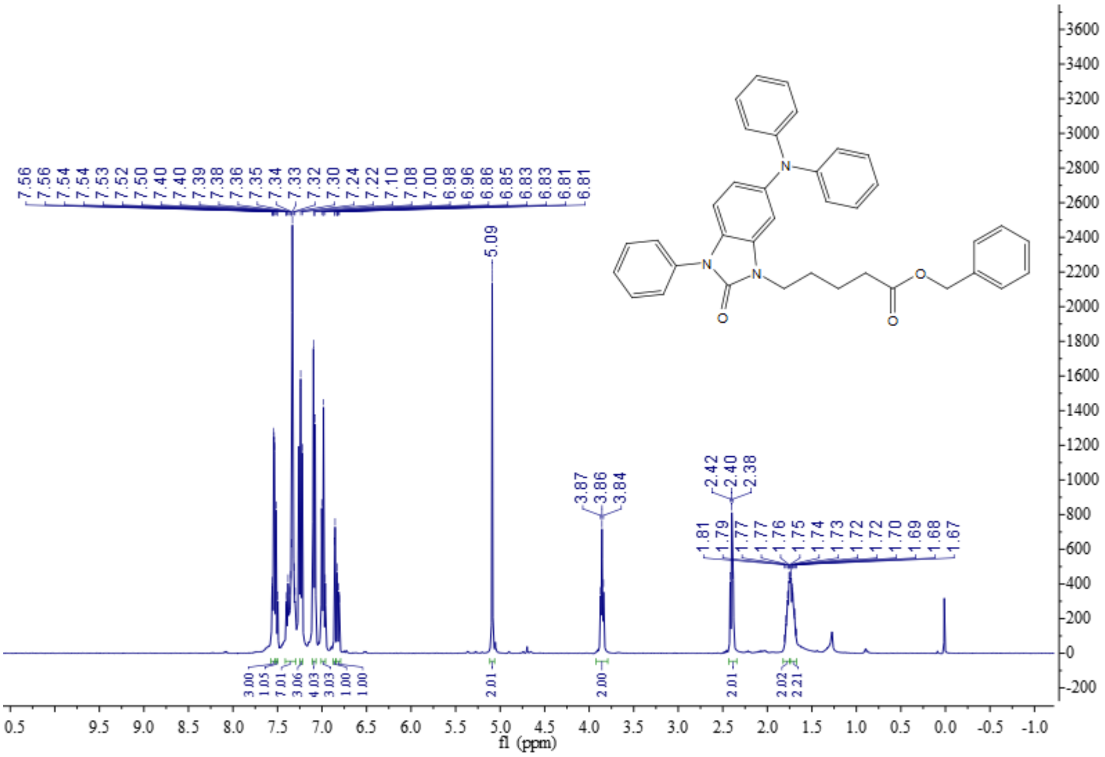

Figure S33. ^{13}C-NMR (100 MHz, $CDCl_3$) spectrum of **4aae**, related to **Scheme 3**.

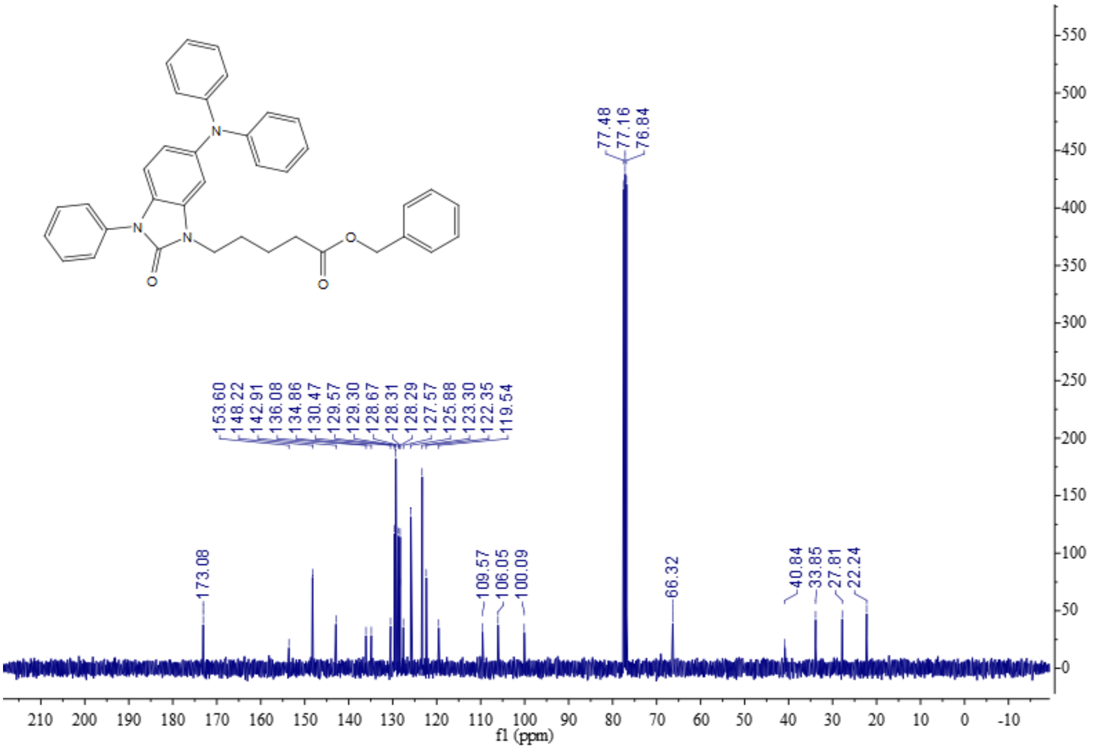

Figure S34. ^{1}H-NMR (400 MHz, $CDCl_3$) spectrum of **4aaf**, related to **Scheme 3**.

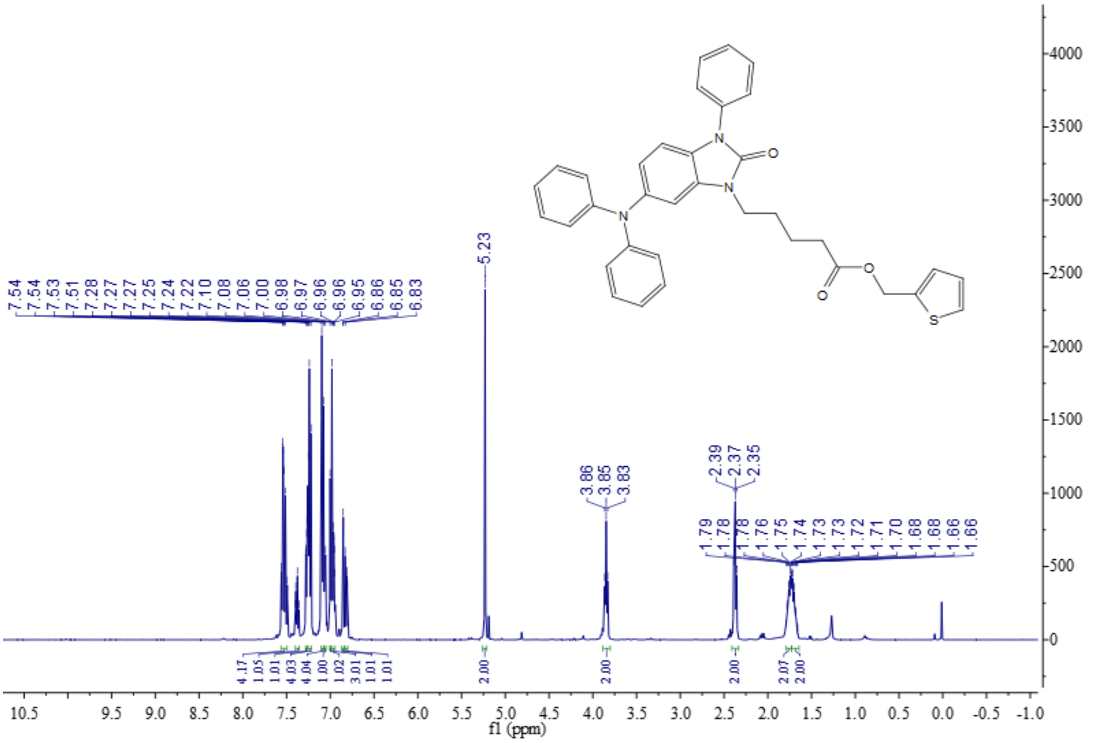

Figure S35. ^{13}C-NMR (100 MHz, $CDCl_3$) spectrum of **4aaf**, related to **Scheme 3**.

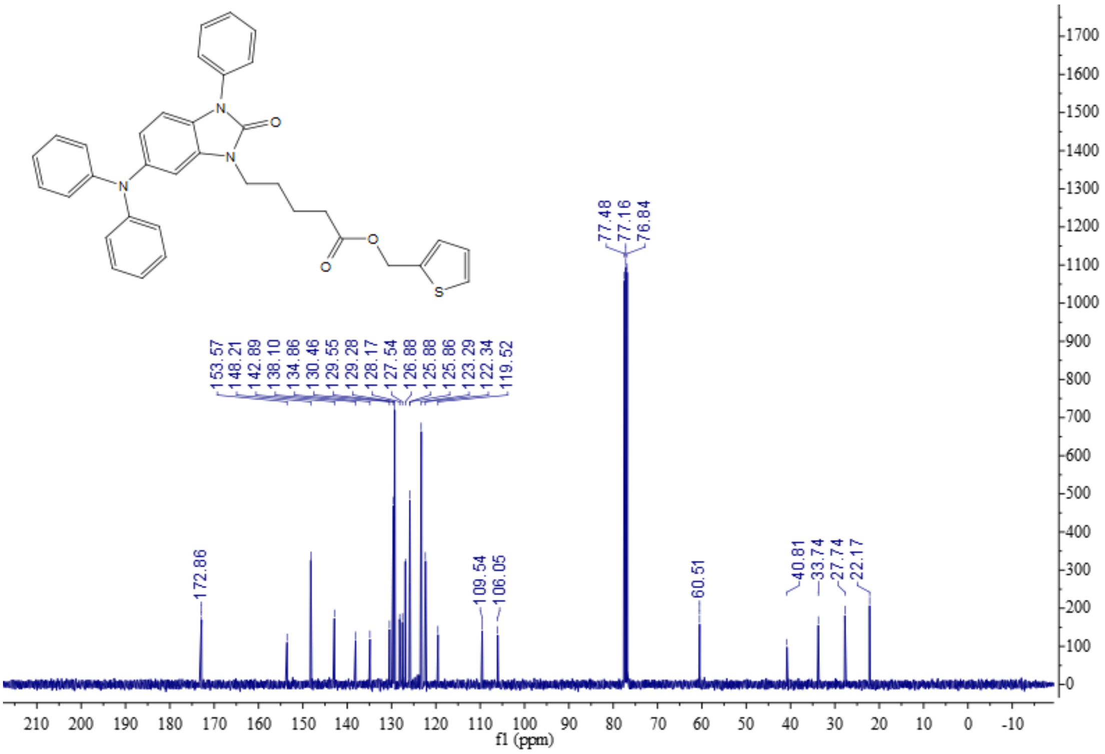

Figure S36. ^{1}H-NMR (400 MHz, $CDCl_3$) spectrum of **4aag**, related to **Scheme 3**.

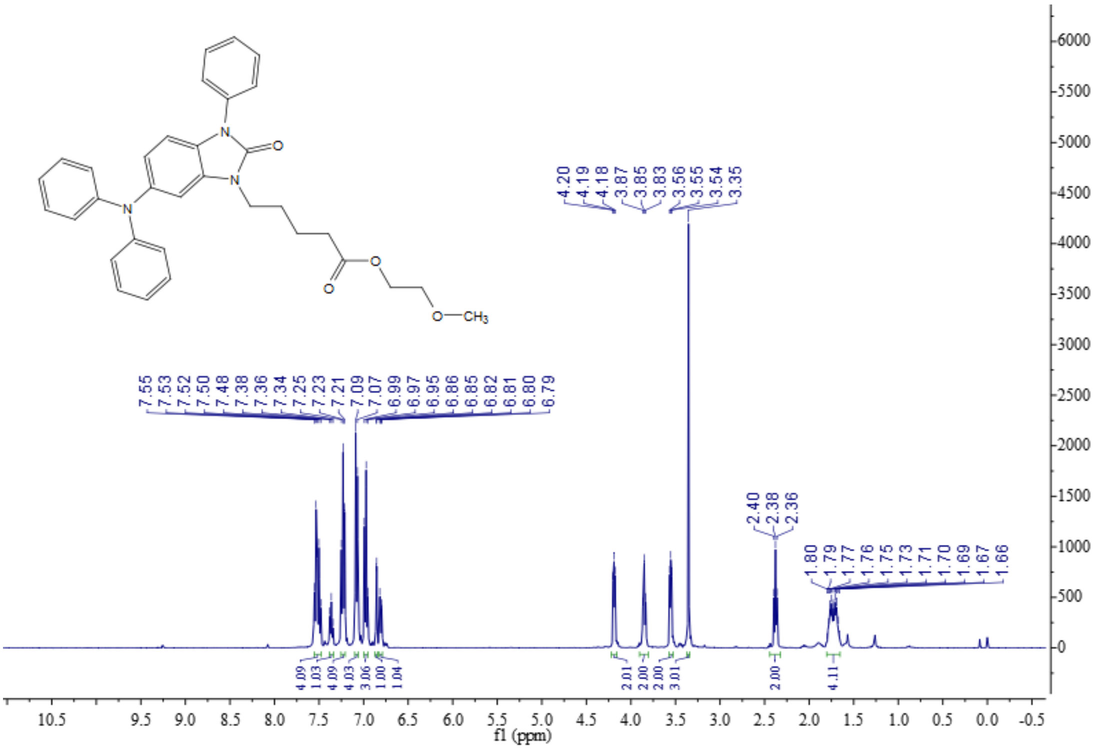

Figure S37. ^{13}C-NMR (100 MHz, $CDCl_3$) spectrum of **4aag**, related to **Scheme 3**.

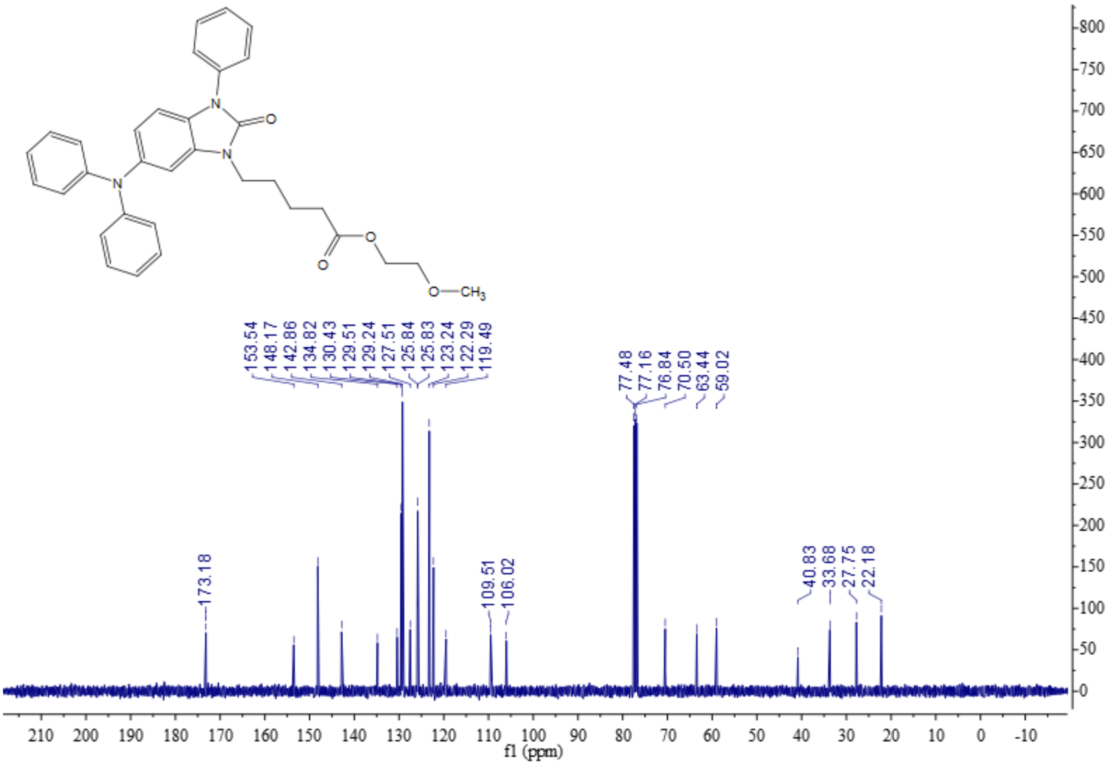

Figure S38. ^{1}H-NMR (400 MHz, $CDCl_3$) spectrum of **5af**, related to **Scheme 4**.

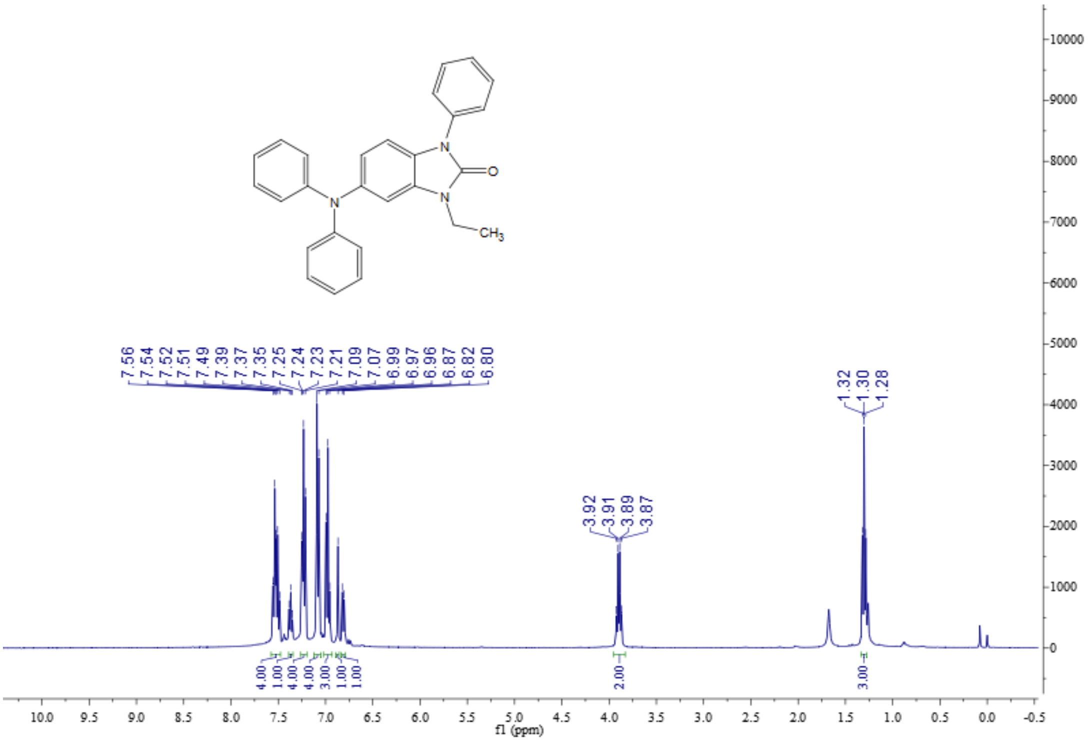

Figure S39. ^{13}C-NMR (100 MHz, $CDCl_3$) spectrum of **5af**, related to **Scheme 4**.

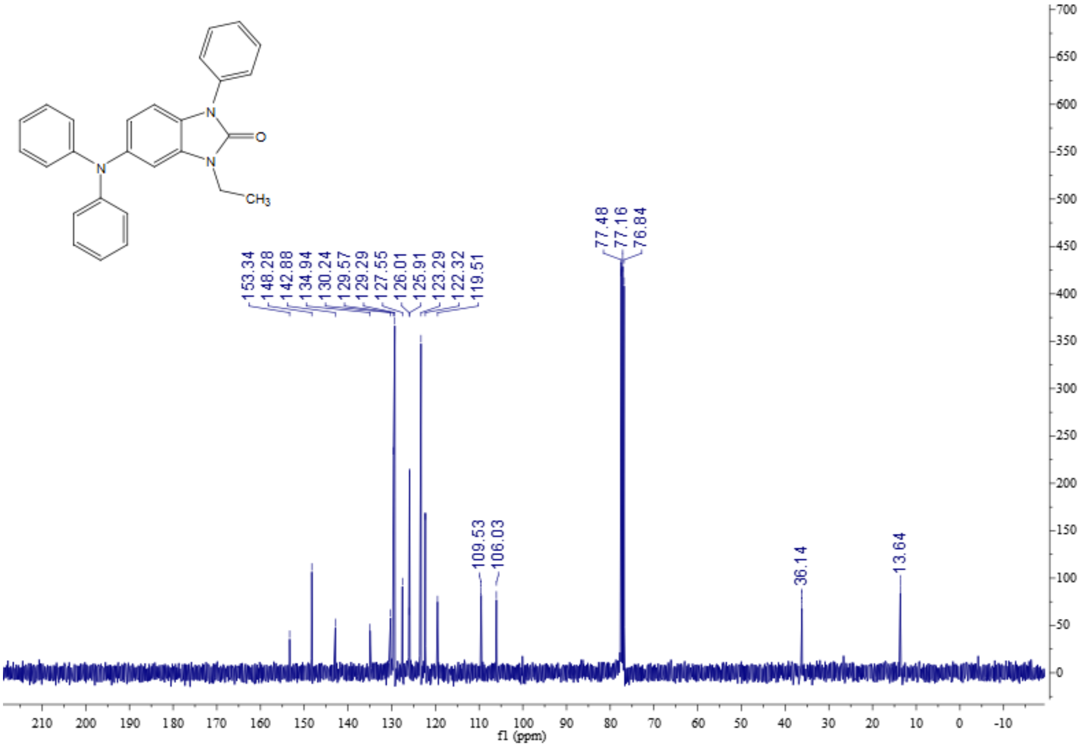

Figure S40. ^{1}H-NMR (400 MHz, $CDCl_3$) spectrum of **5ag**, related to **Scheme 4**.

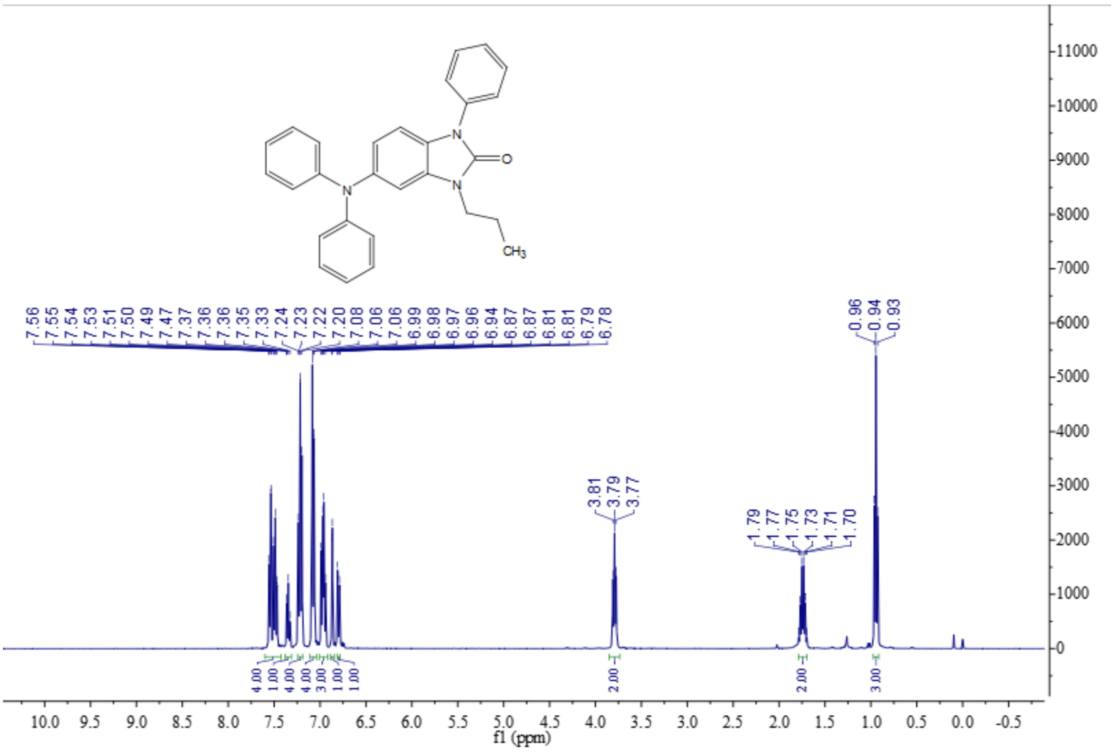

Figure S41. ^{13}C-NMR (100 MHz, $CDCl_3$) spectrum of **5ag**, related to **Scheme 4**.

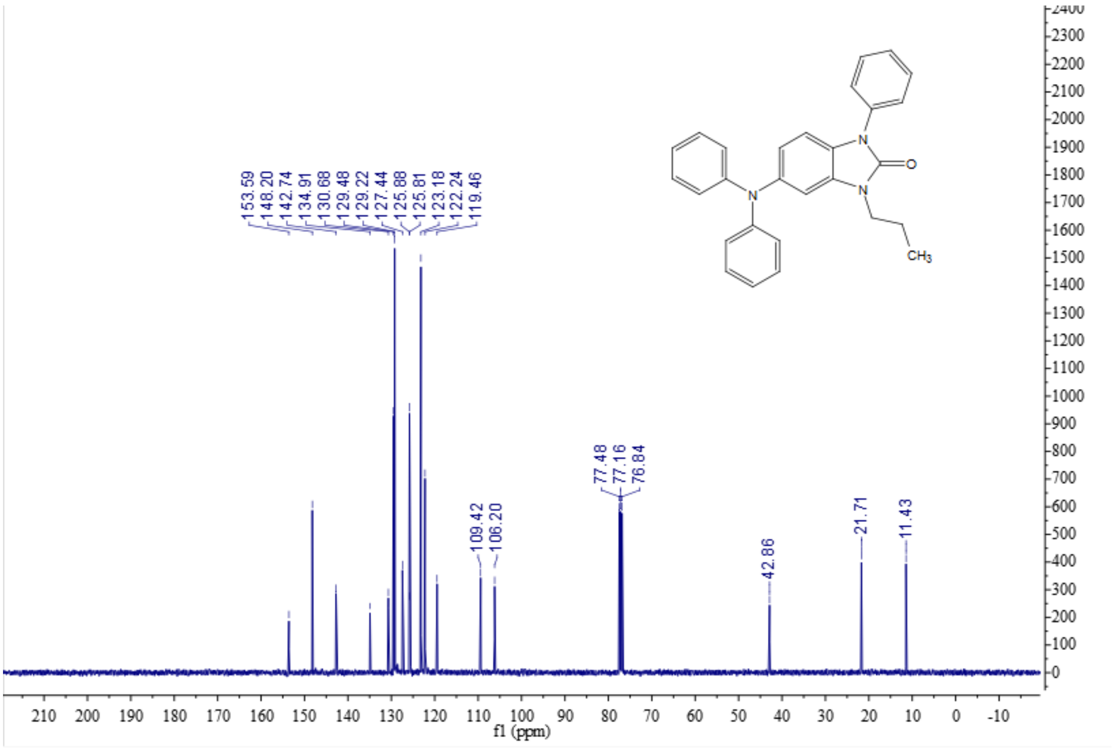

Figure S42. ^{1}H-NMR (400 MHz, $CDCl_3$) spectrum of **5ah**, related to **Scheme 4**.

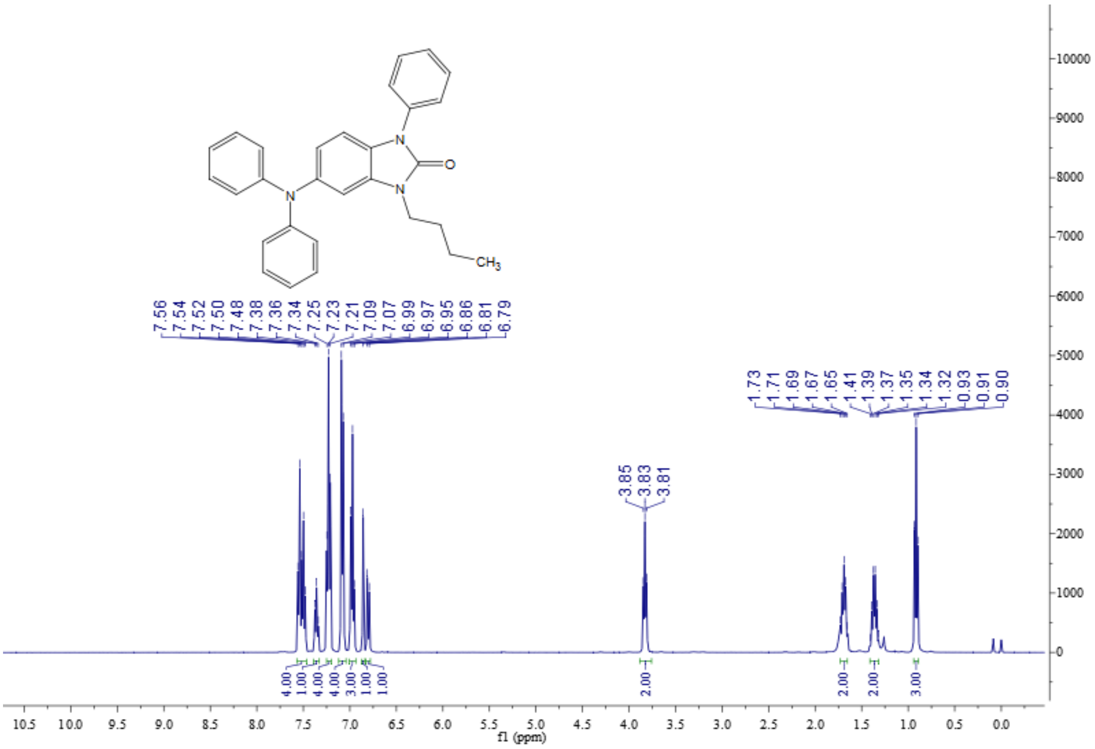

Figure S43. ^{13}C-NMR (100 MHz, $CDCl_3$) spectrum of **5ah**, related to **Scheme 4**.

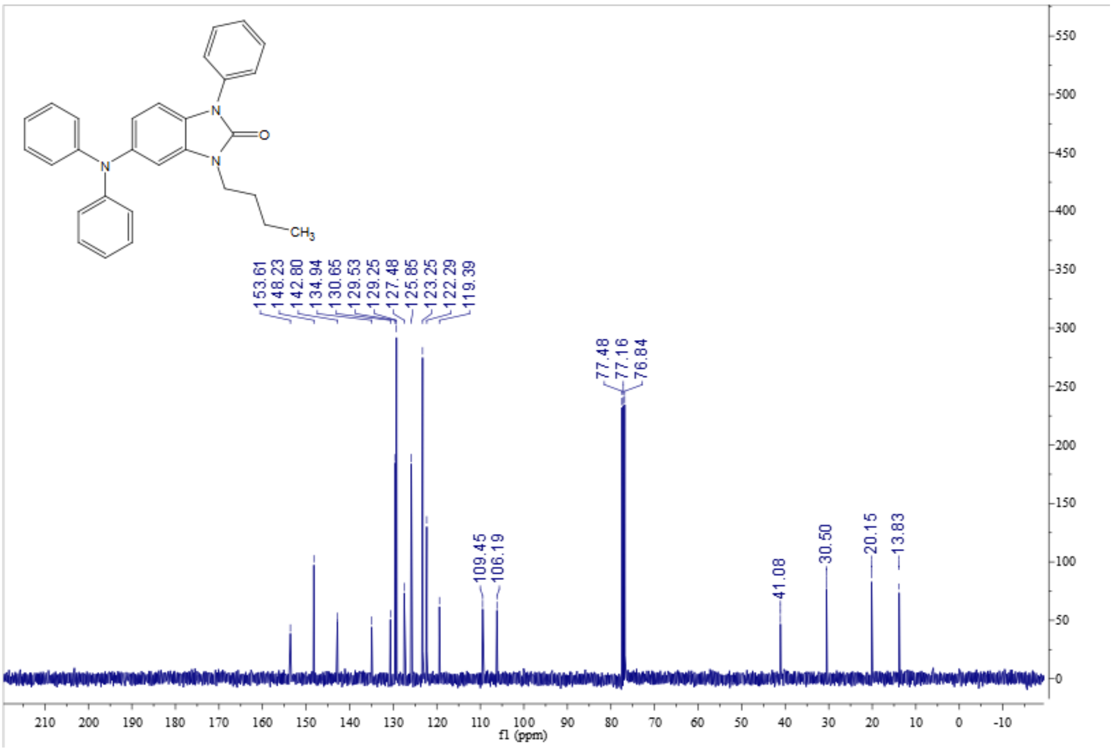

Figure S44. ^{1}H-NMR (400 MHz, $CDCl_3$) spectrum of **5ai**, related to **Scheme 4**.

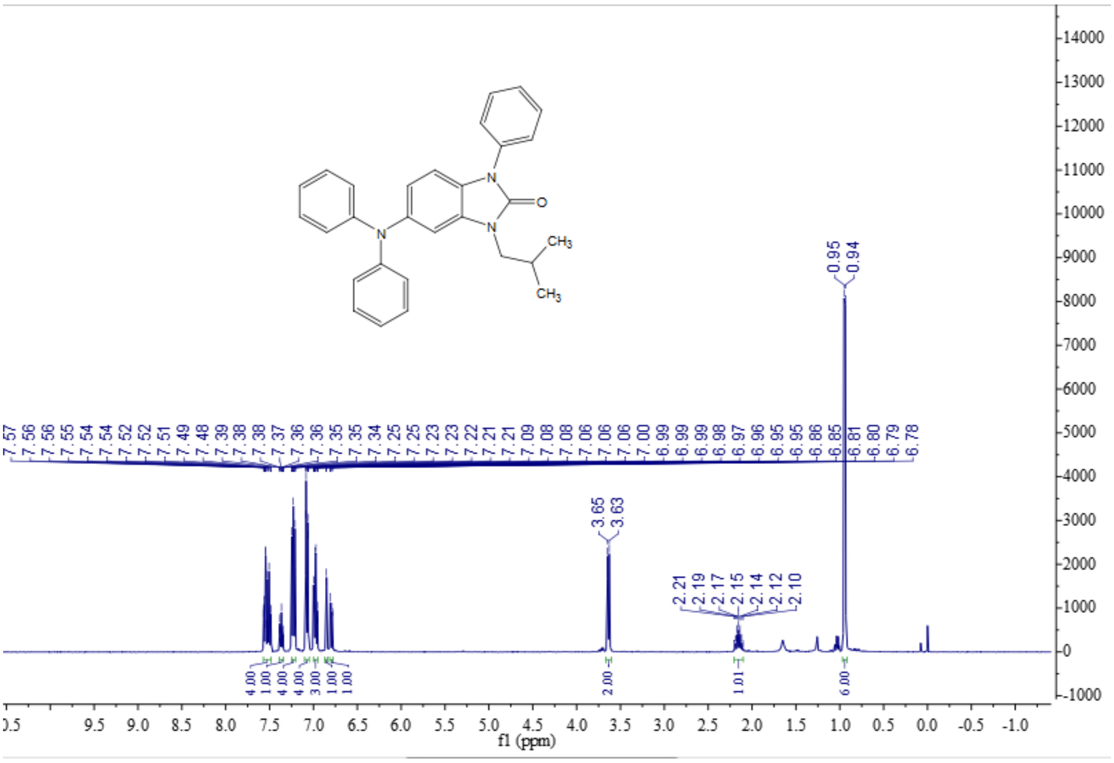

Figure S45. ^{13}C-NMR (100 MHz, $CDCl_3$) spectrum of **5ai**, related to **Scheme 4**.

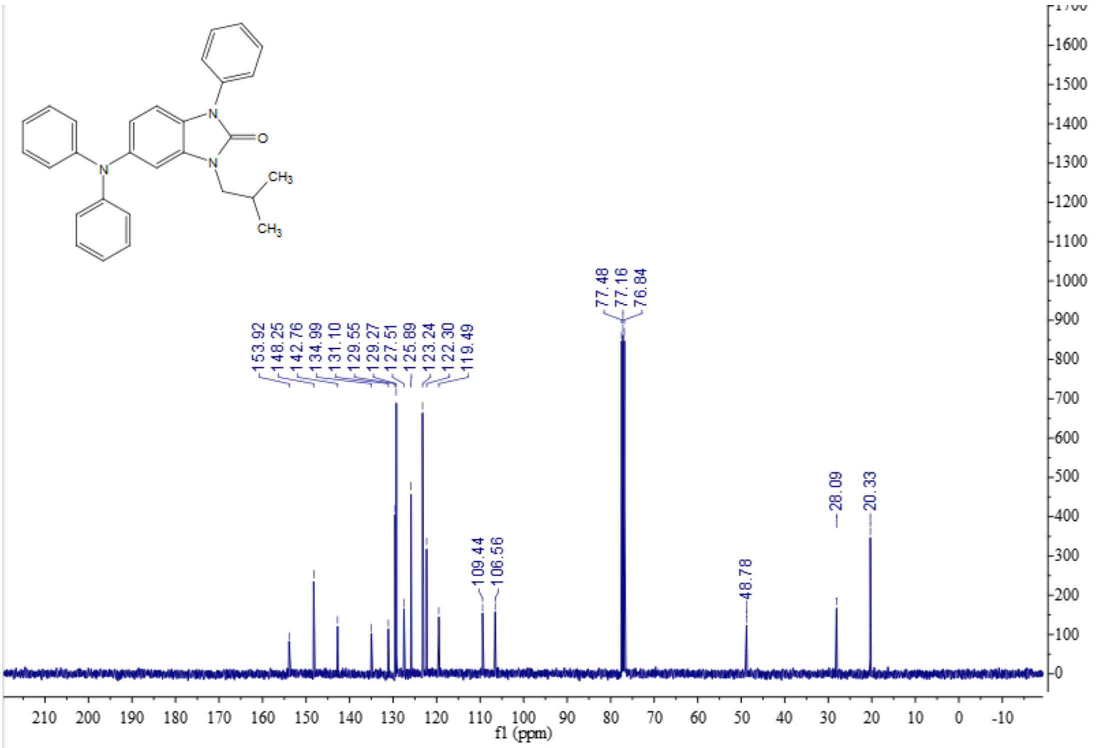

Figure S46. ^{1}H-NMR (400 MHz, $CDCl_3$) spectrum of **5aj**, related to **Scheme 4**.

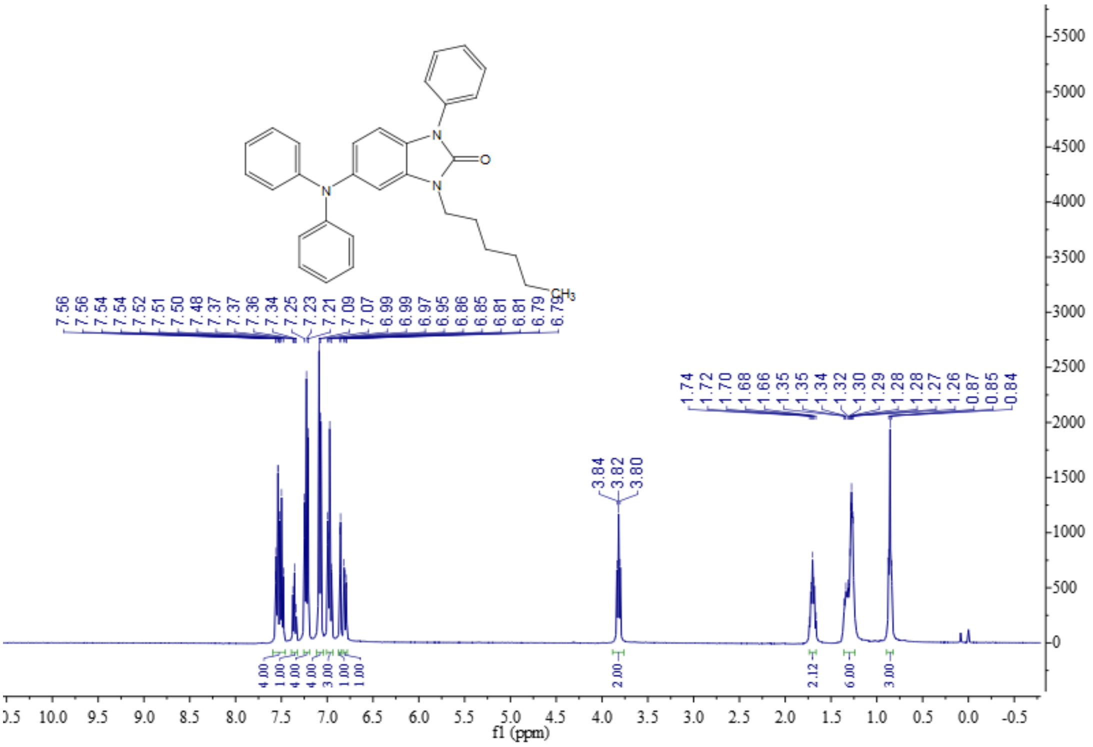

Figure S47. ^{13}C-NMR (100 MHz, $CDCl_3$) spectrum of **5aj**, related to **Scheme 4**.

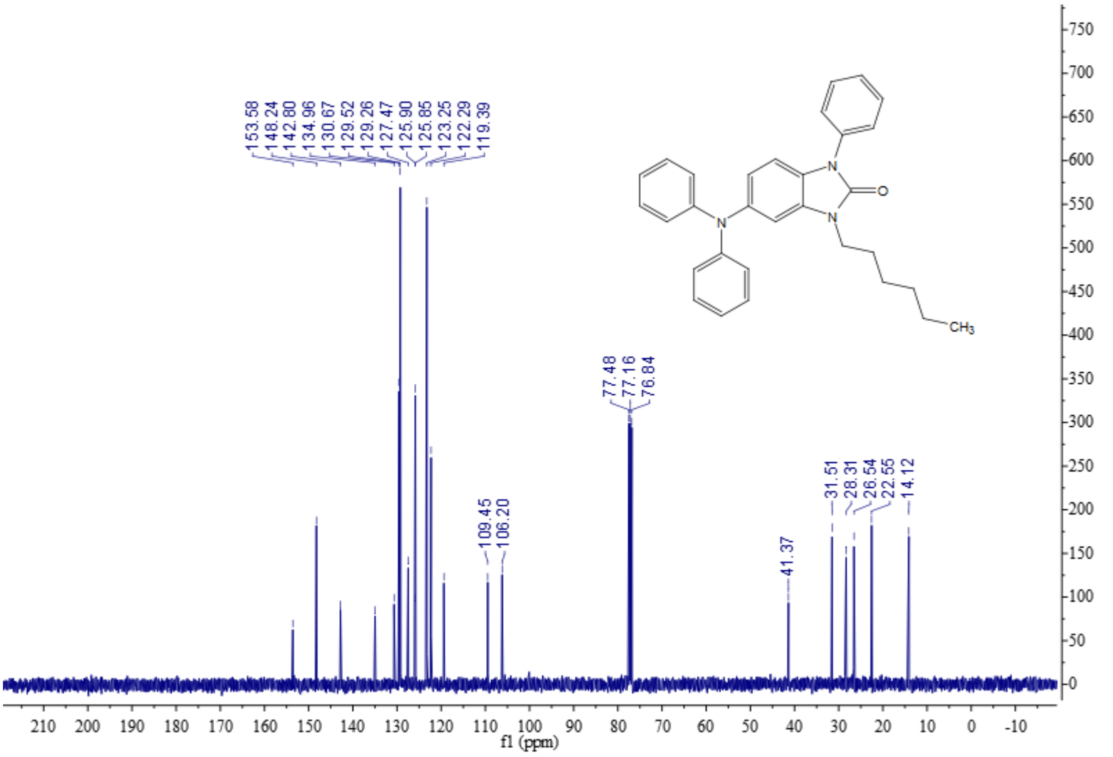

Figure S48. ^{1}H-NMR (400 MHz, $CDCl_3$) spectrum of **5al**, related to **Scheme 4**.

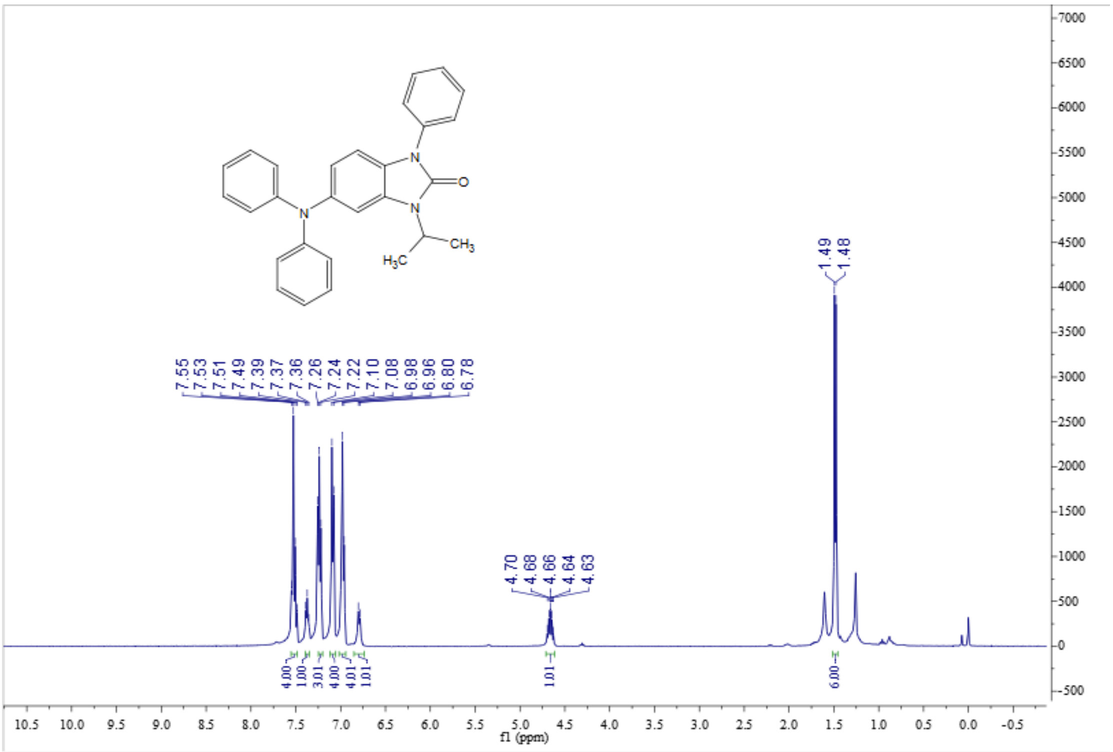

Figure S49. ^{13}C-NMR (100 MHz, $CDCl_3$) spectrum of **5al**, related to **Scheme 4**.

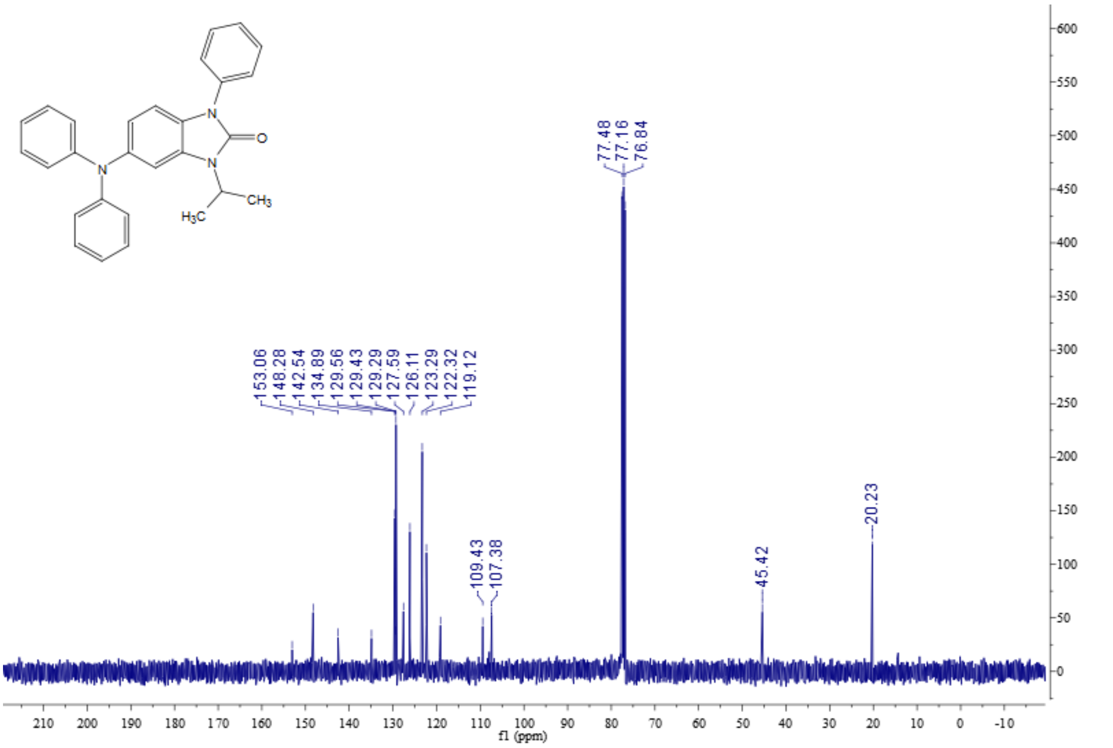

Figure S50. ^{1}H-NMR (400 MHz, $CDCl_3$) spectrum of **5am**, related to **Scheme 4**.

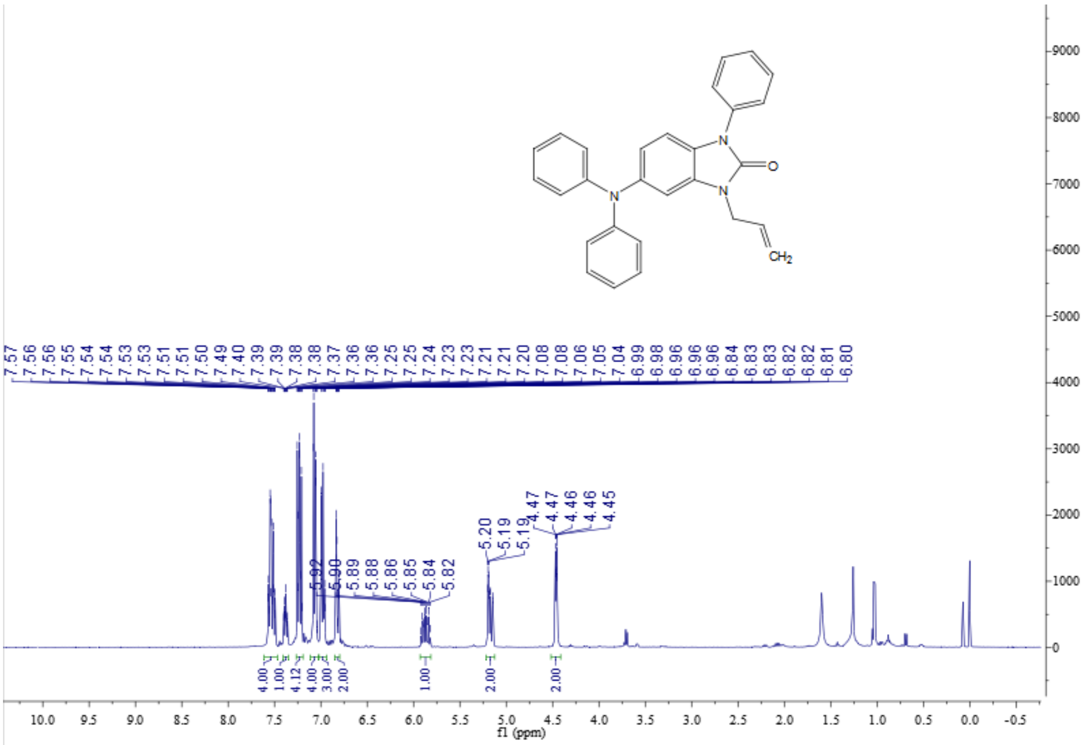

Figure S51. ^{13}C-NMR (100 MHz, $CDCl_3$) spectrum of **5am**, related to **Scheme 4**.

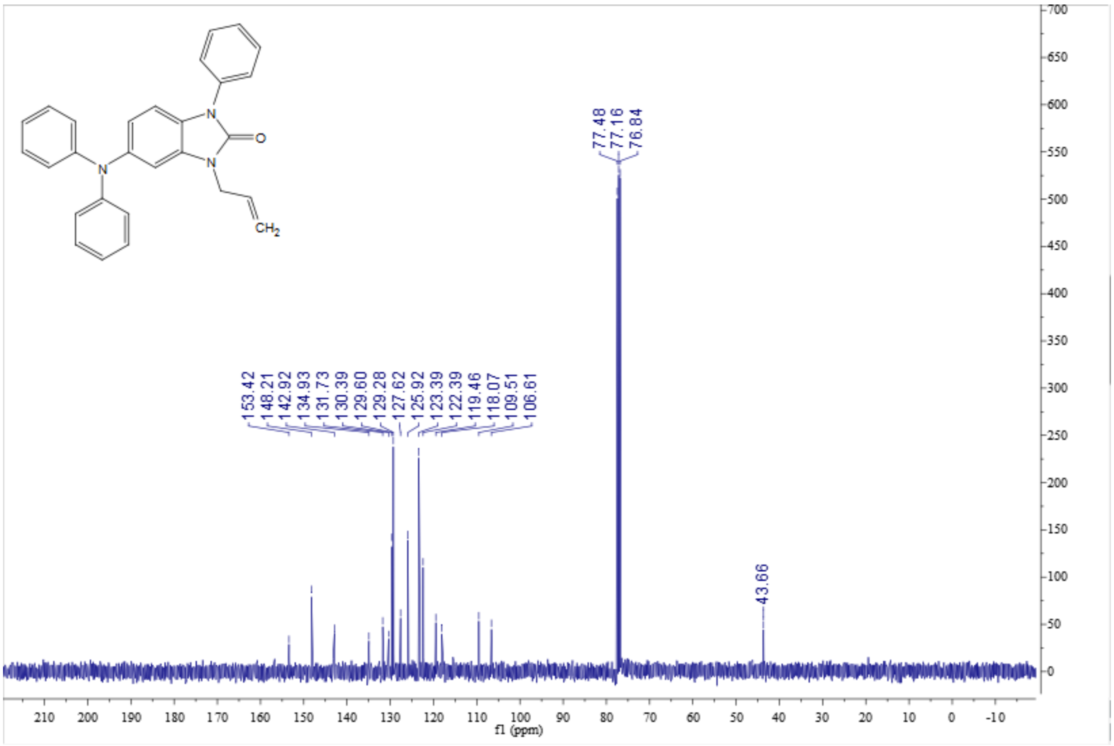

Figure S52. ^{1}H-NMR (400 MHz, $CDCl_3$) spectrum of **5bf**, related to **Scheme 5**.

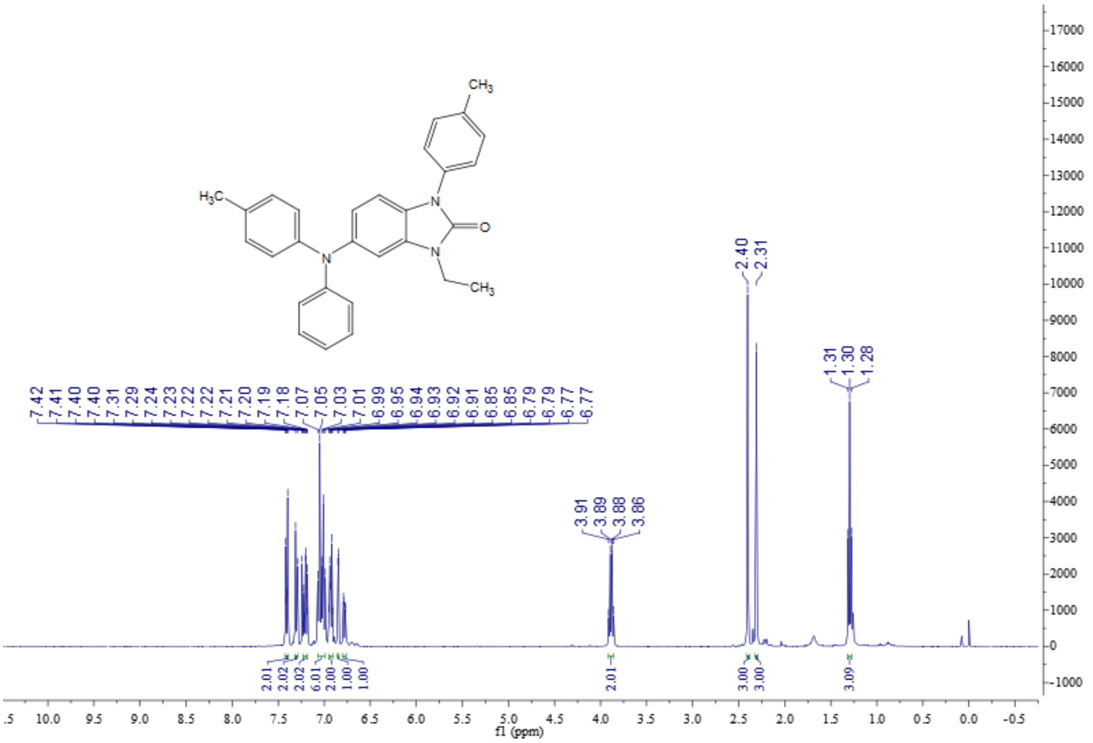

Figure S53. ^{13}C-NMR (100 MHz, $CDCl_3$) spectrum of **5bf**, related to **Scheme 5**.

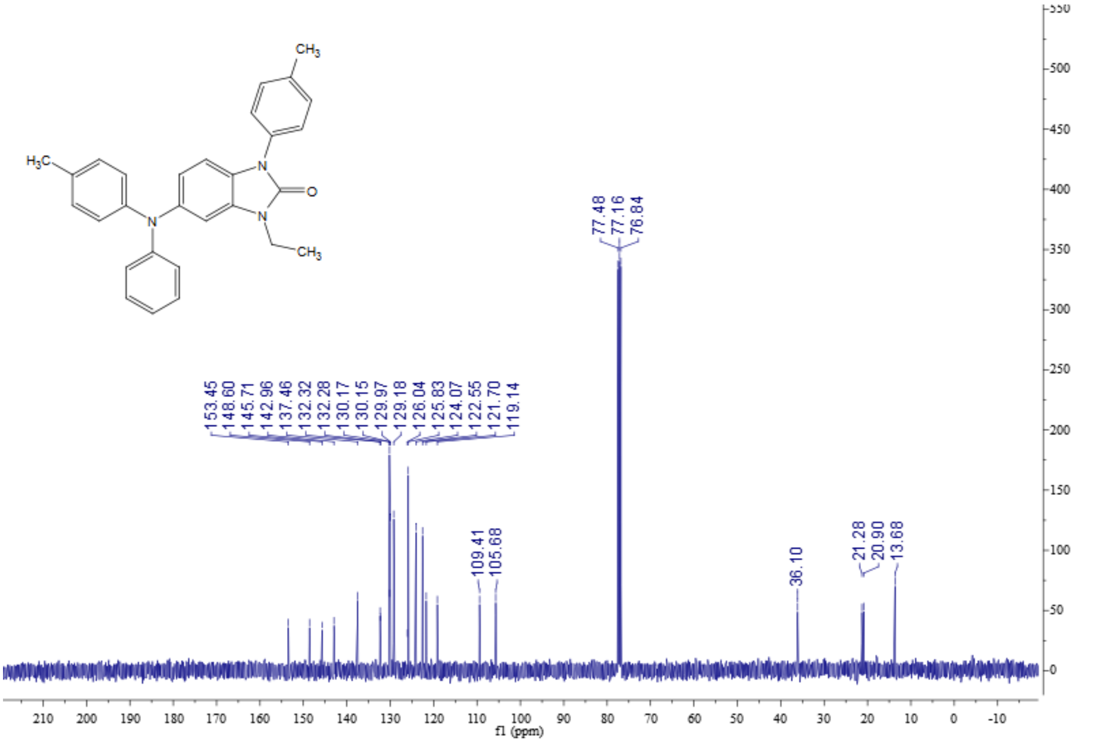

Figure S54. ^{1}H-NMR (400 MHz, $CDCl_3$) spectrum of **5cf**, related to **Scheme 5**.

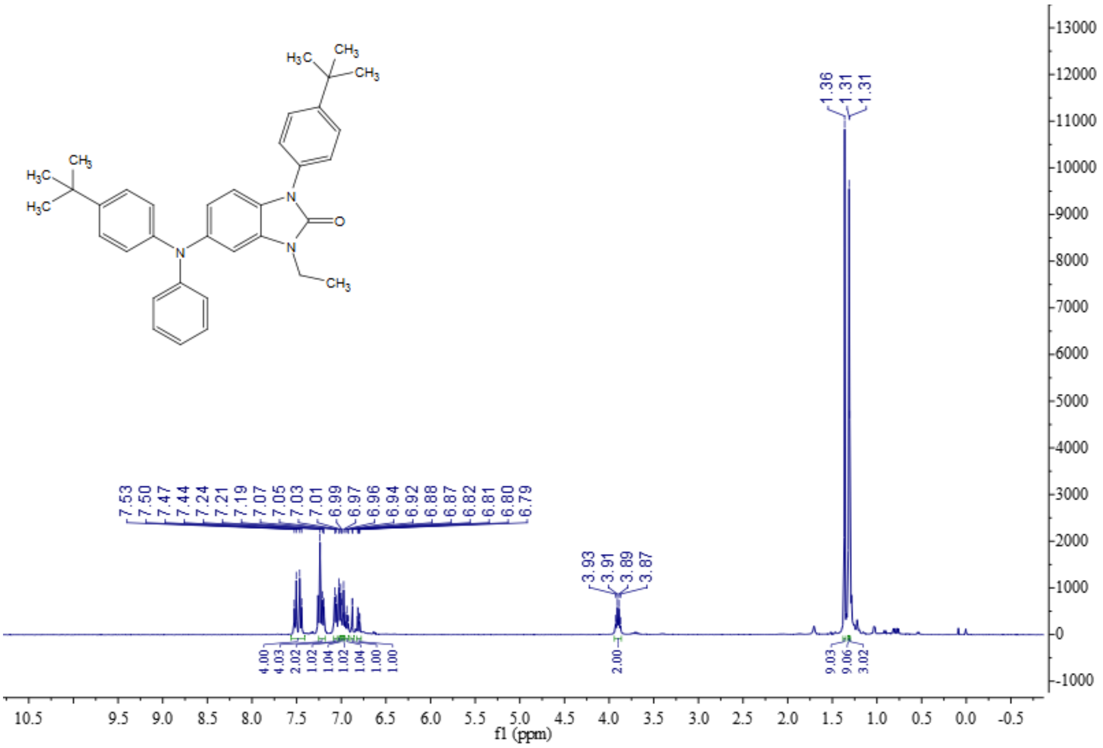

Figure S55. ^{13}C-NMR (100 MHz, $CDCl_3$) spectrum of **5cf**, related to **Scheme 5**.

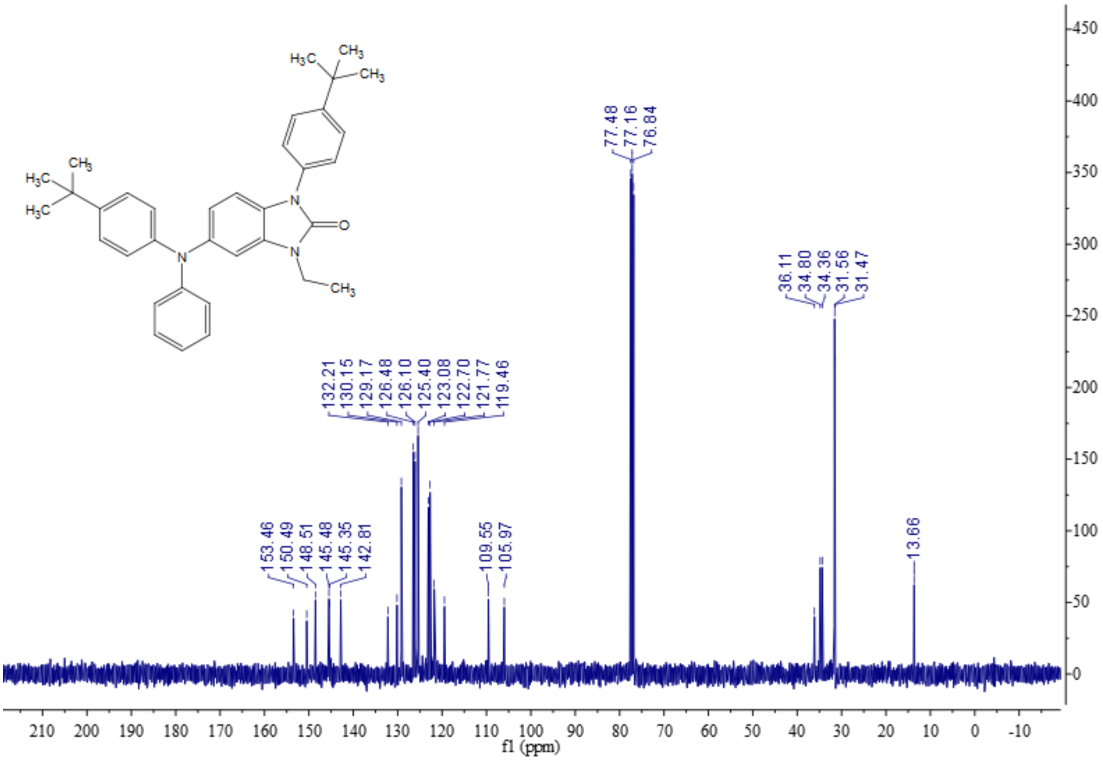

Figure S56. ^{1}H-NMR (400 MHz, $CDCl_3$) spectrum of **5df**, related to **Scheme 5**.

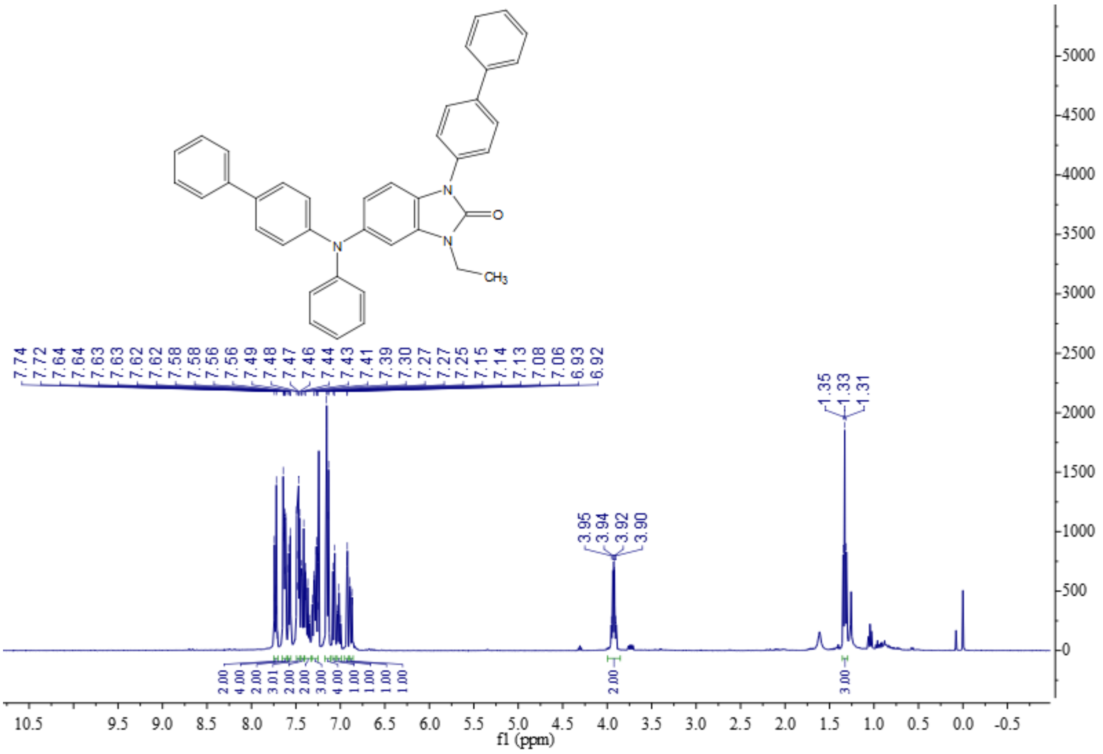

Figure S57. ^{13}C-NMR (100 MHz, $CDCl_3$) spectrum of **5df**, related to **Scheme 5**.

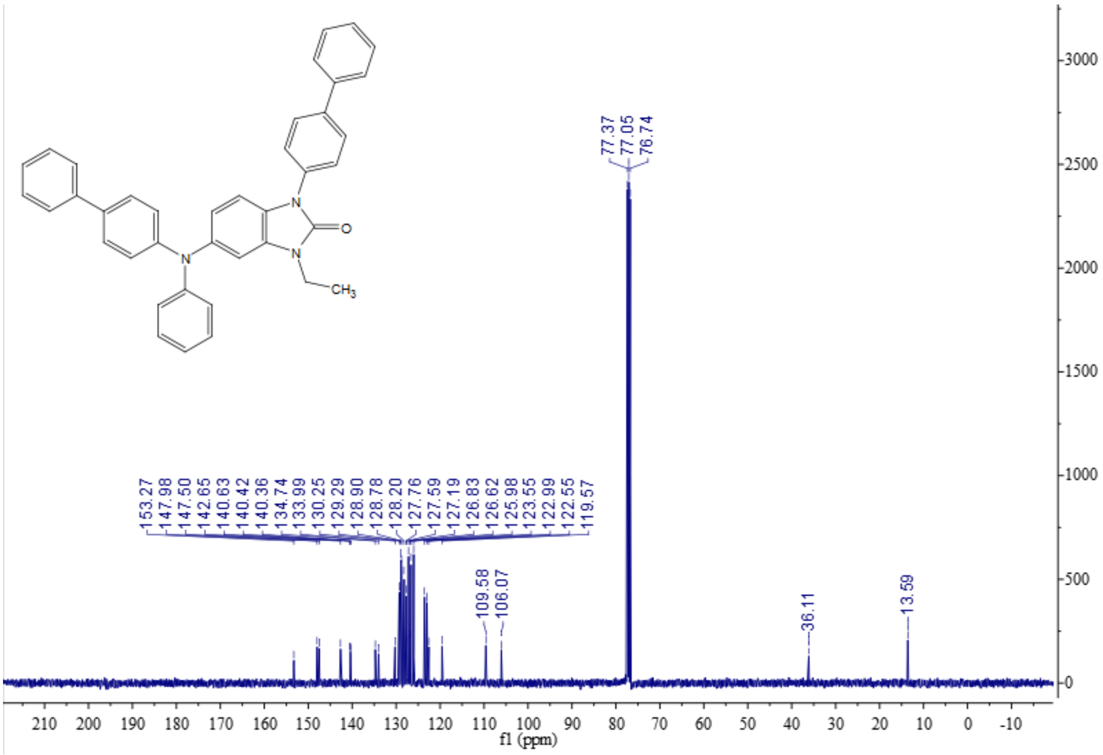

Figure S58. ^{1}H-NMR (400 MHz, $CDCl_3$) spectrum of **5ef**, related to **Scheme 5**.

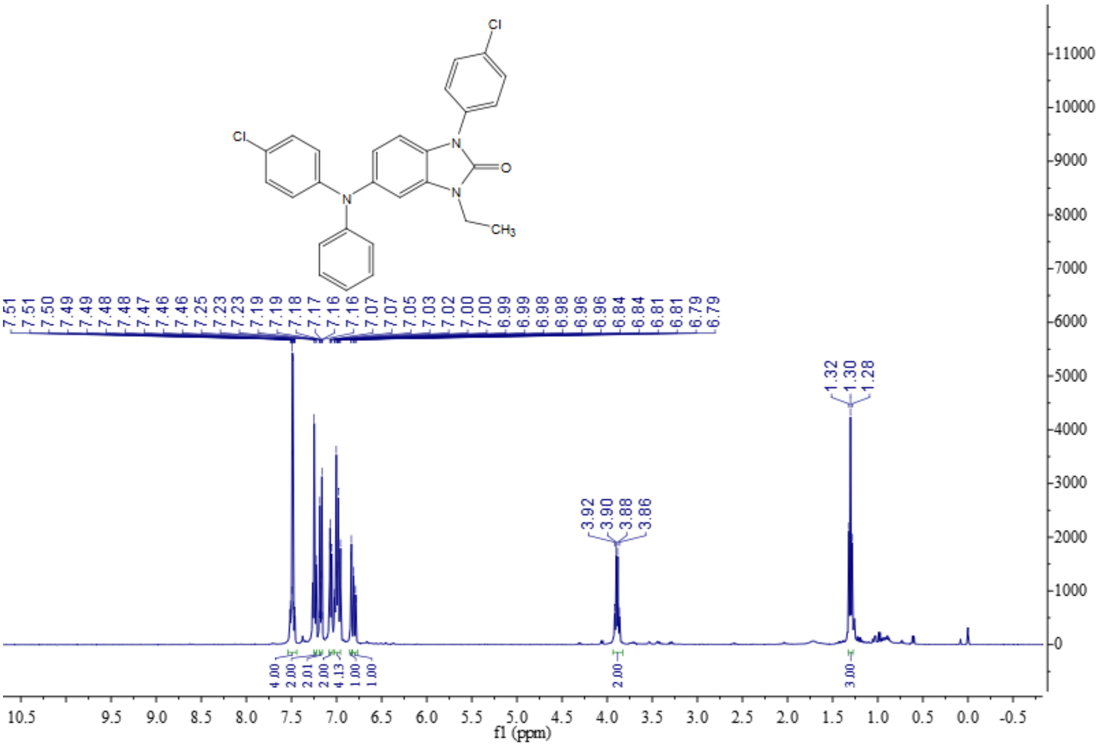

Figure S59. ^{13}C-NMR (100 MHz, $CDCl_3$) spectrum of **5ef**, related to **Scheme 5**.

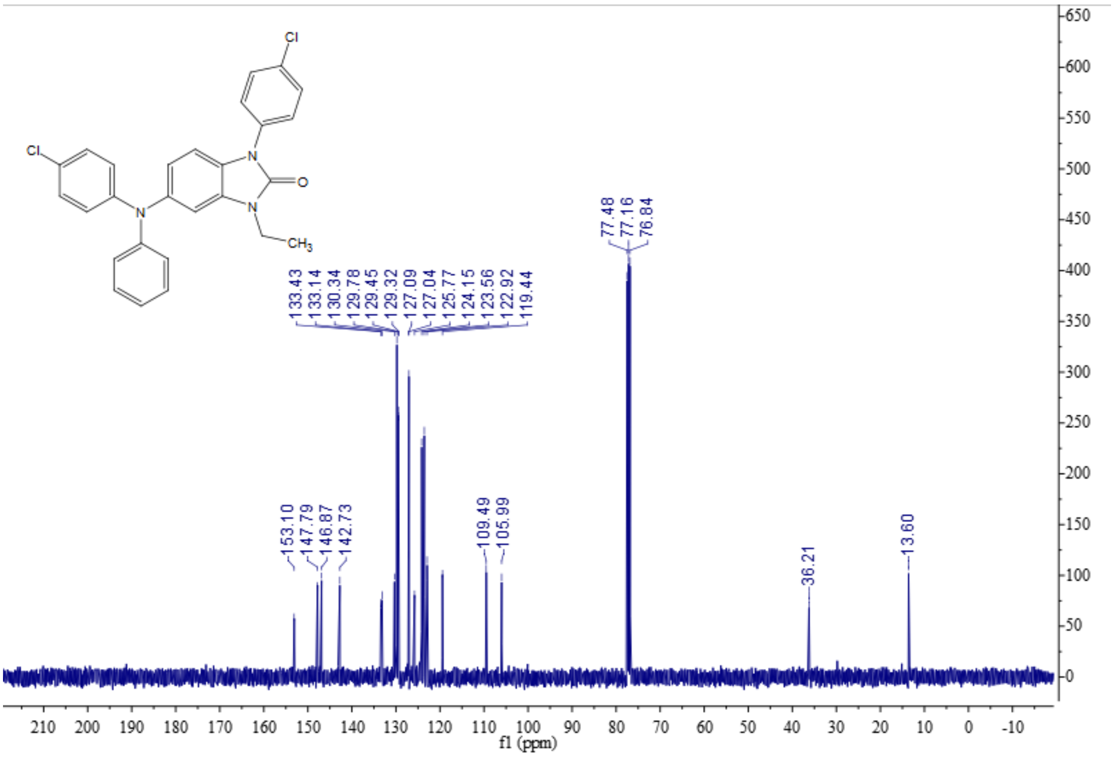

Figure S60. ^{1}H-NMR (400 MHz, $CDCl_3$) spectrum of **5ff**, related to **Scheme 5**.

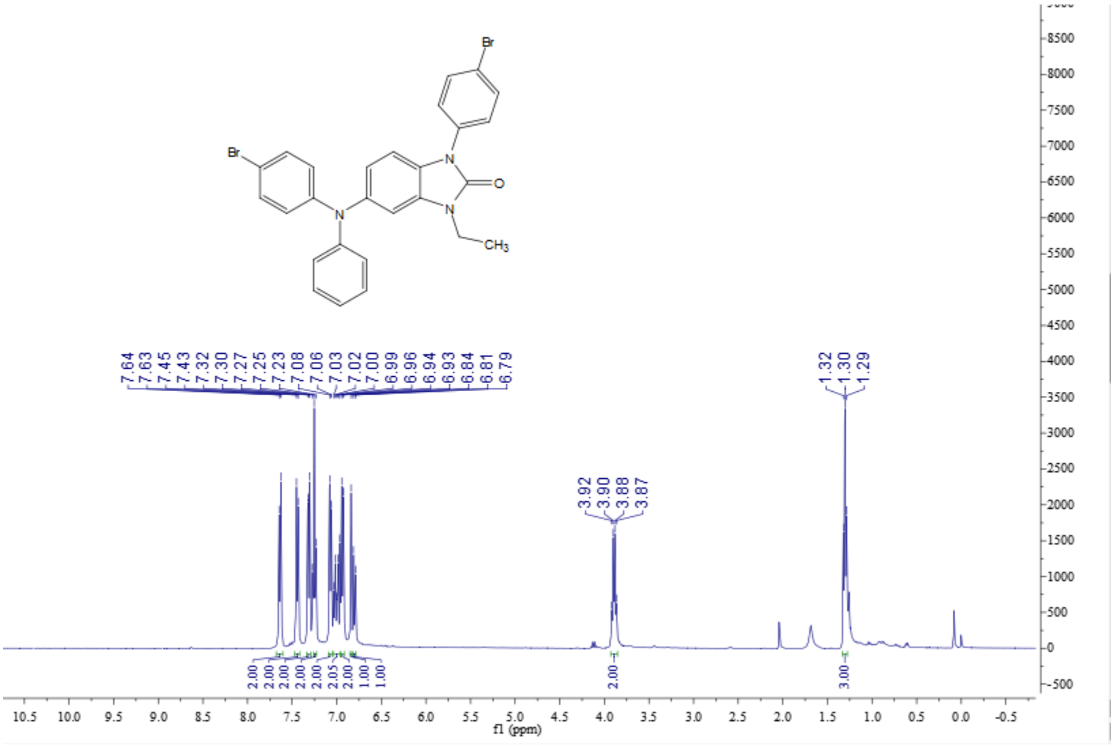

Figure S61. ^{13}C-NMR (100 MHz, $CDCl_3$) spectrum of **5ff**, related to **Scheme 5**.

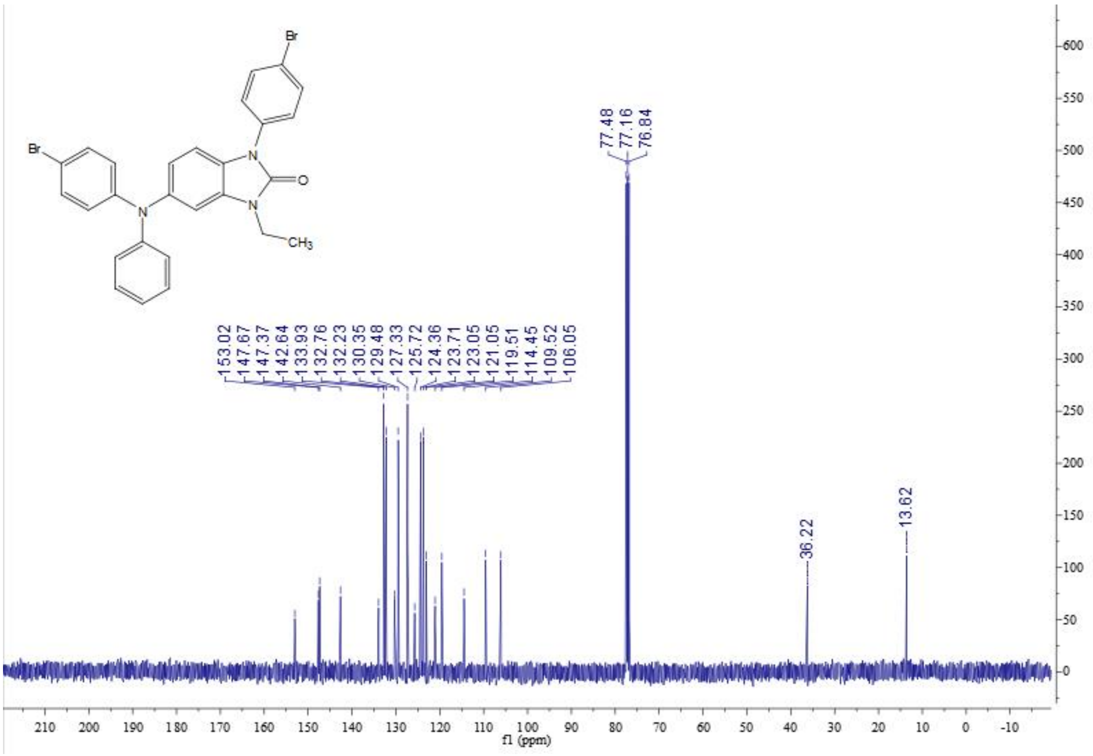

Figure S62. ^{1}H-NMR (400 MHz, $CDCl_3$) spectrum of **5if**, related to **Scheme 5**.

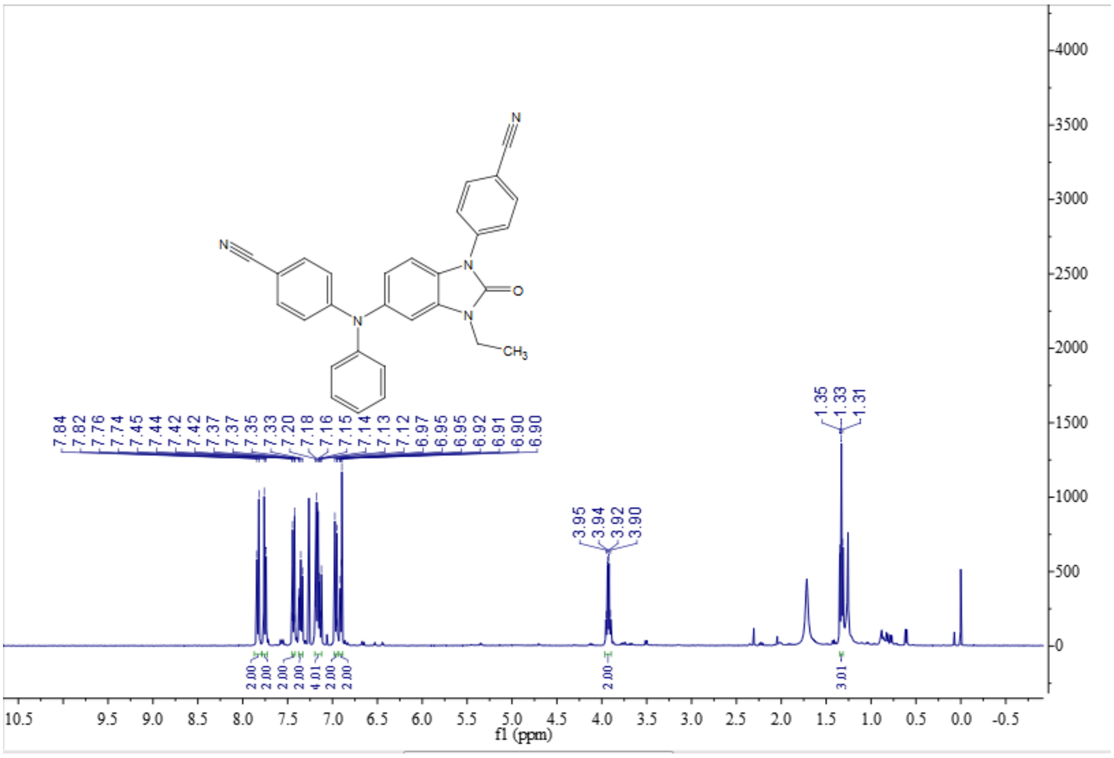

Figure S63. ^{13}C-NMR (100 MHz, $CDCl_3$) spectrum of **5if**, related to **Scheme 5**.

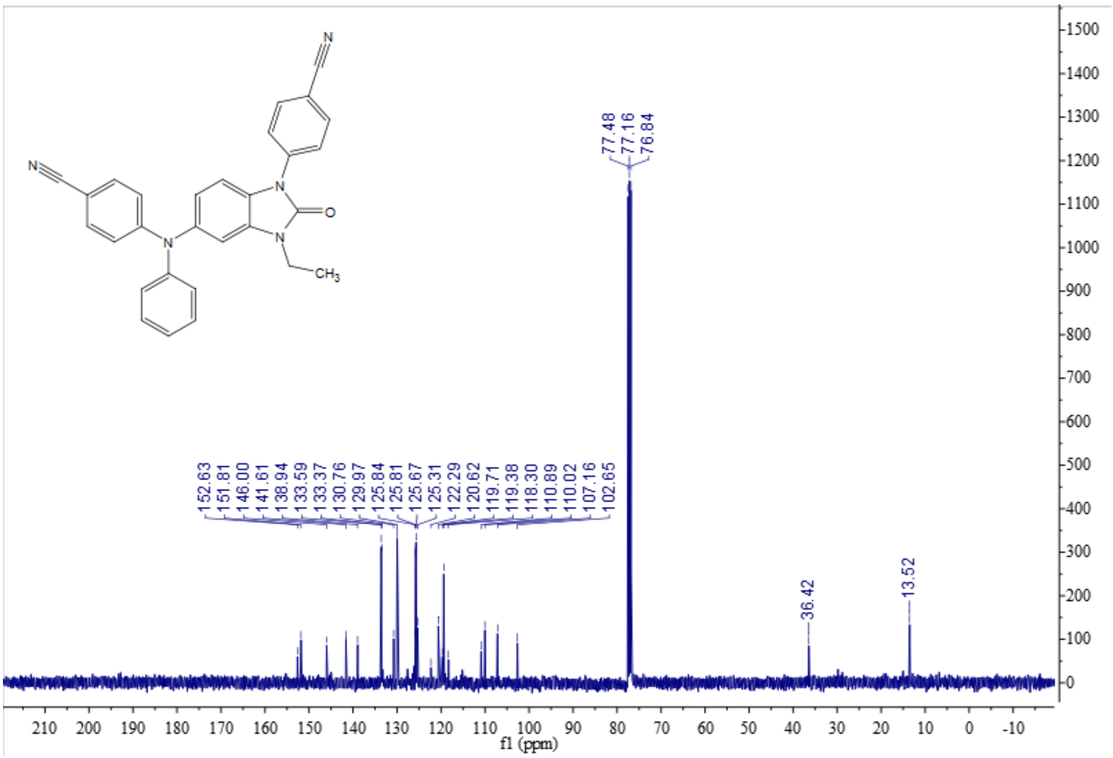

Figure S64. ^{1}H-NMR (400 MHz, $CDCl_3$) spectrum of **5bg**, related to **Scheme 5**.

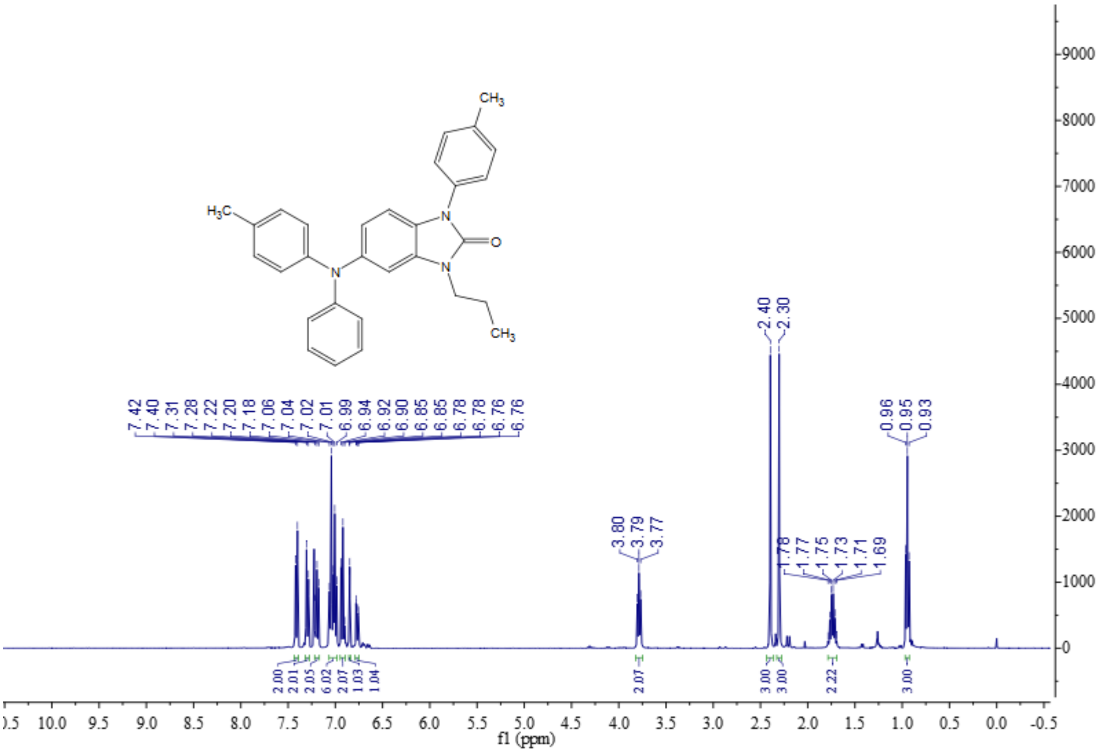

Figure S65. ^{13}C-NMR (100 MHz, $CDCl_3$) spectrum of **5bg**, related to **Scheme 5**.

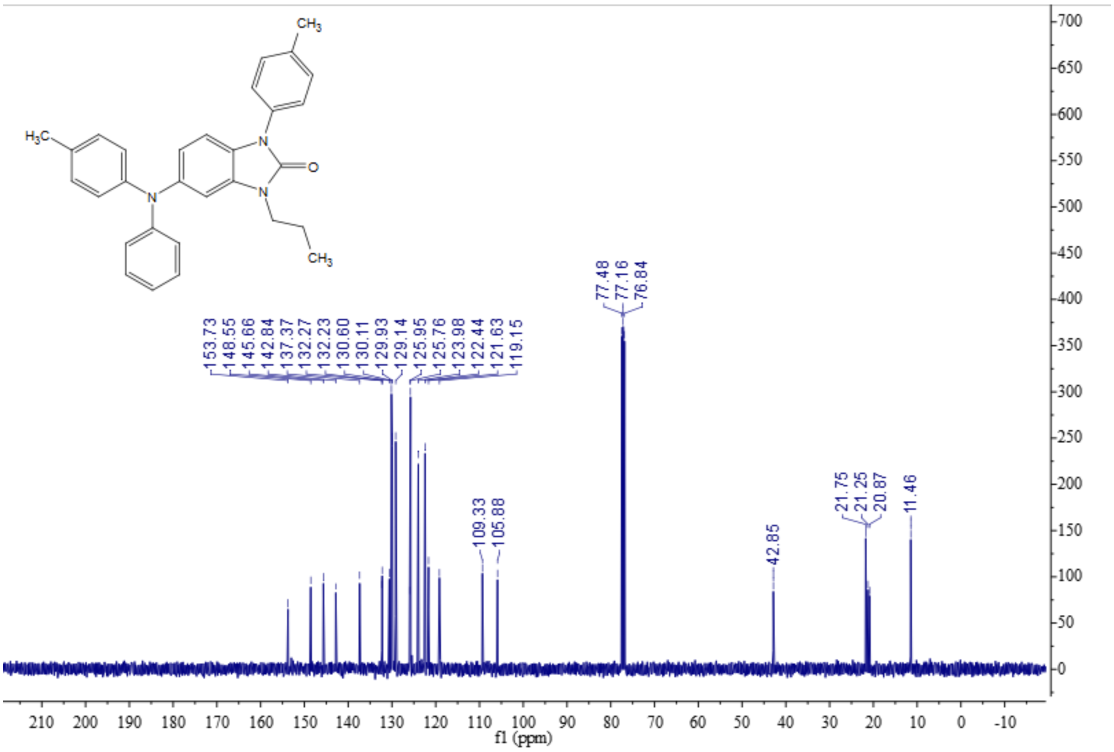

Figure S66. ^{1}H-NMR (400 MHz, $CDCl_3$) spectrum of **5jg**, related to **Scheme 5**.

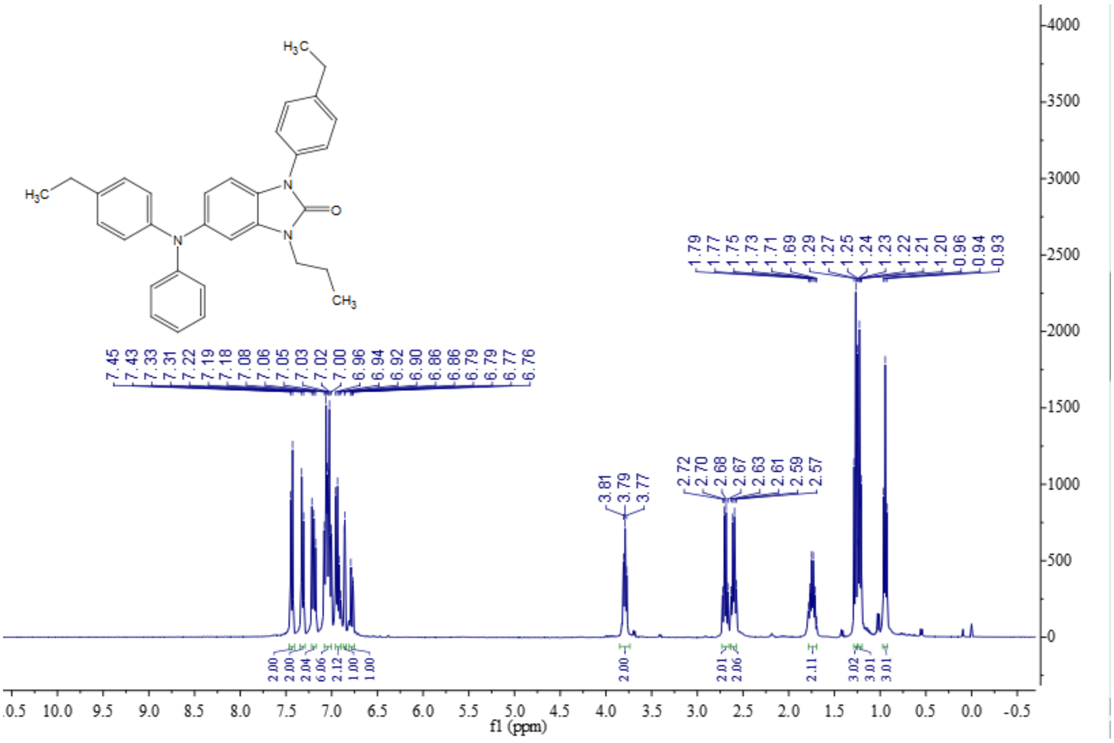

Figure S67. ^{13}C-NMR (100 MHz, $CDCl_3$) spectrum of **5jg**, related to **Scheme 5**.

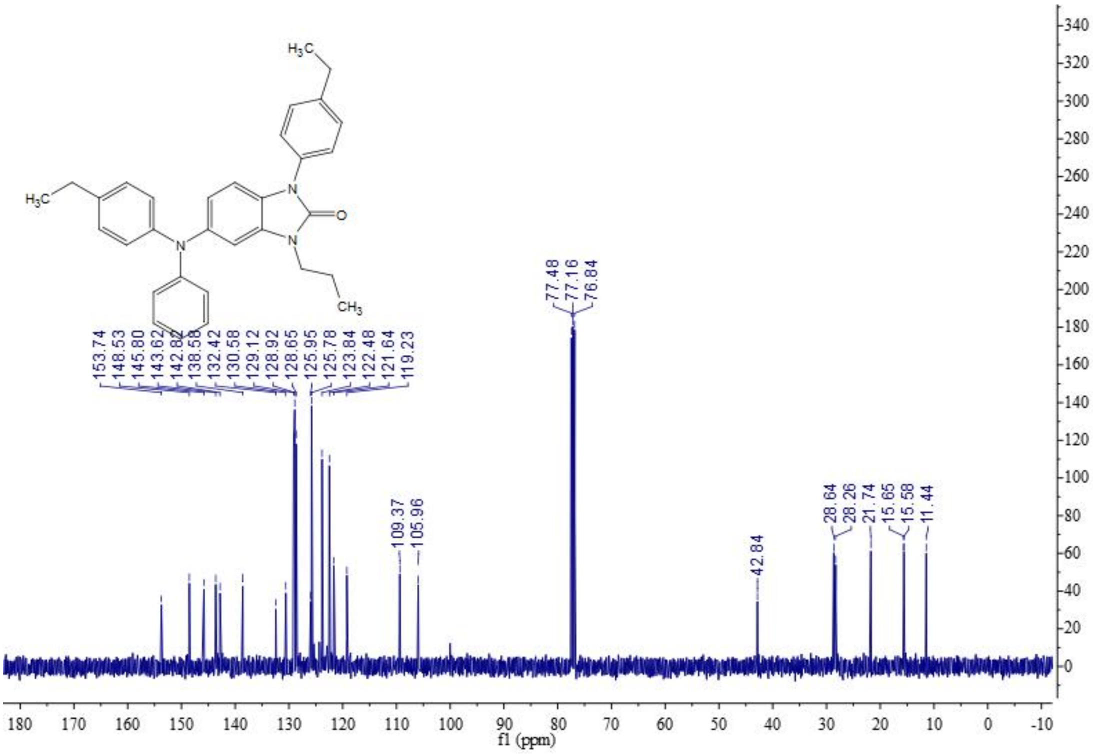

Figure S68. ^{1}H-NMR (400 MHz, $CDCl_3$) spectrum of **5kg**, related to **Scheme 5**.

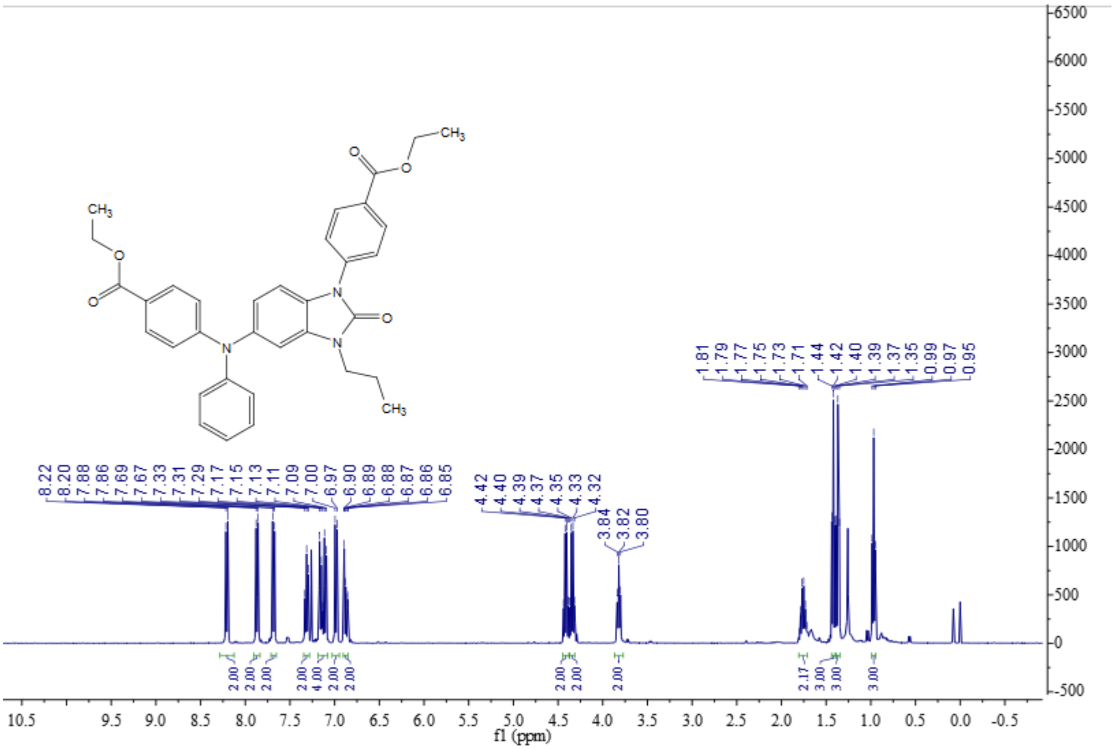

Figure S69. ^{13}C-NMR (100 MHz, $CDCl_3$) spectrum of **5kg**, related to **Scheme 5**.

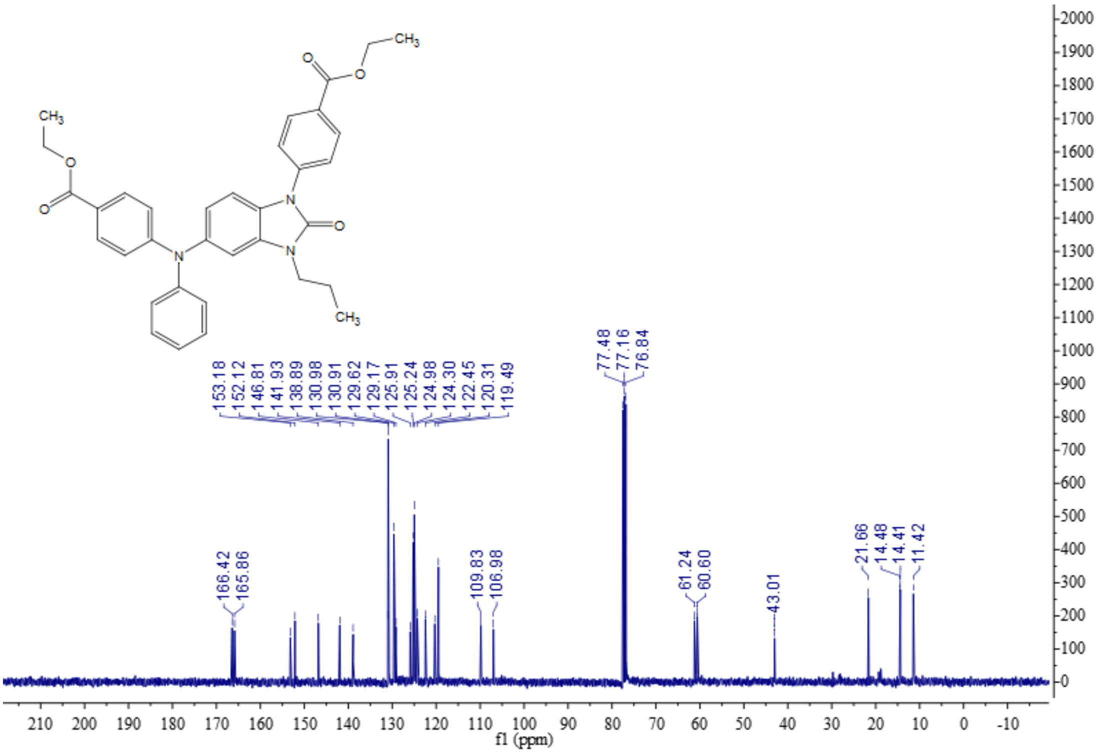

Figure S70. ^{1}H-NMR (400 MHz, $CDCl_3$) spectrum of **5lg**, related to **Scheme 5**.

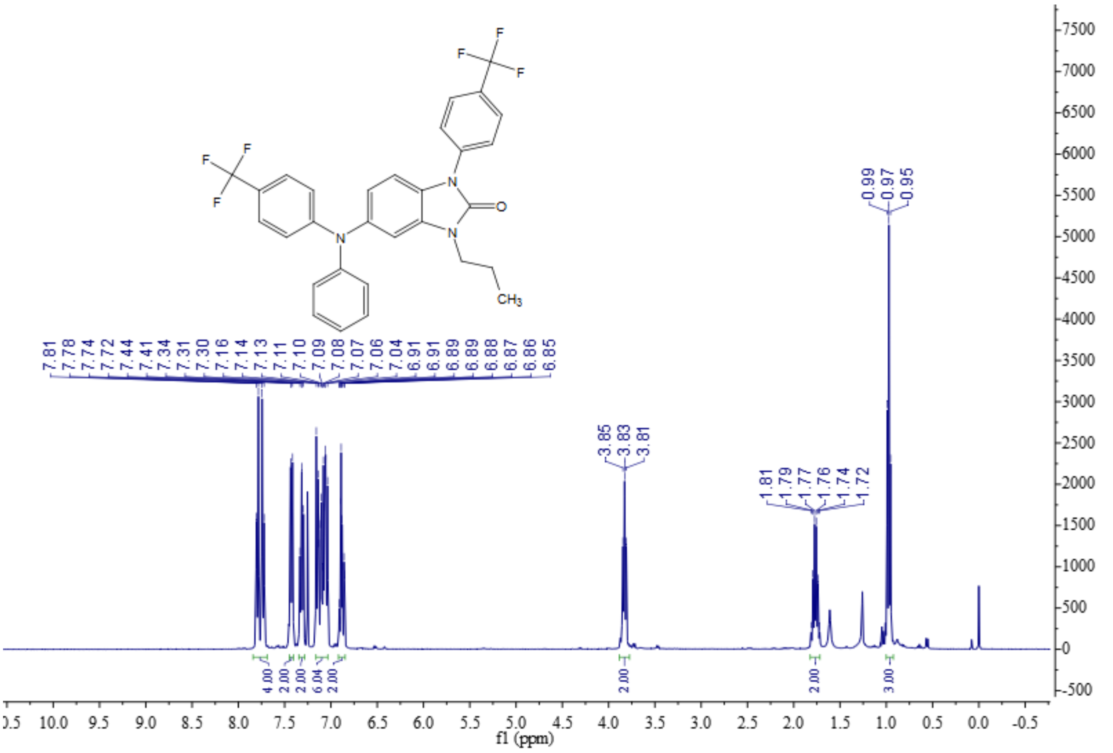

Figure S71. ^{13}C-NMR (100 MHz, $CDCl_3$) spectrum of **5lg**, related to **Scheme 5**.

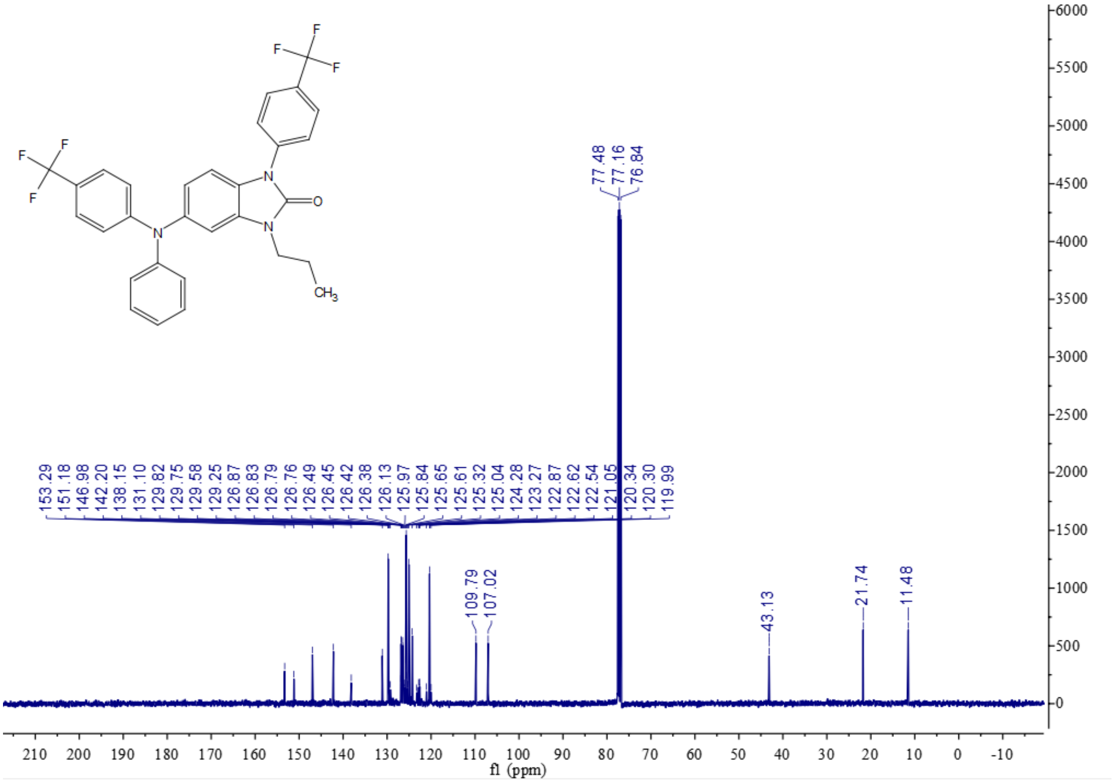

Figure S72. ^{1}H-NMR (400 MHz, $CDCl_3$) spectrum of **5mf**, related to **Scheme 5**.

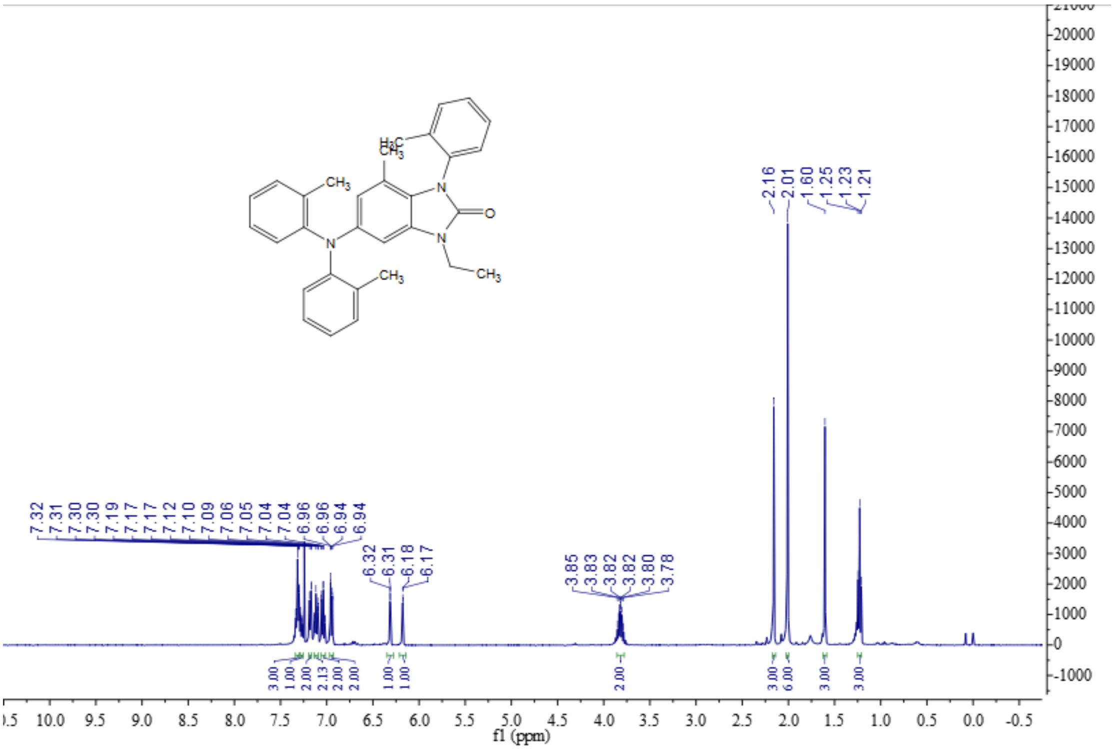

Figure S73. ^{13}C-NMR (100 MHz, $CDCl_3$) spectrum of **5mf**, related to **Scheme 5**.

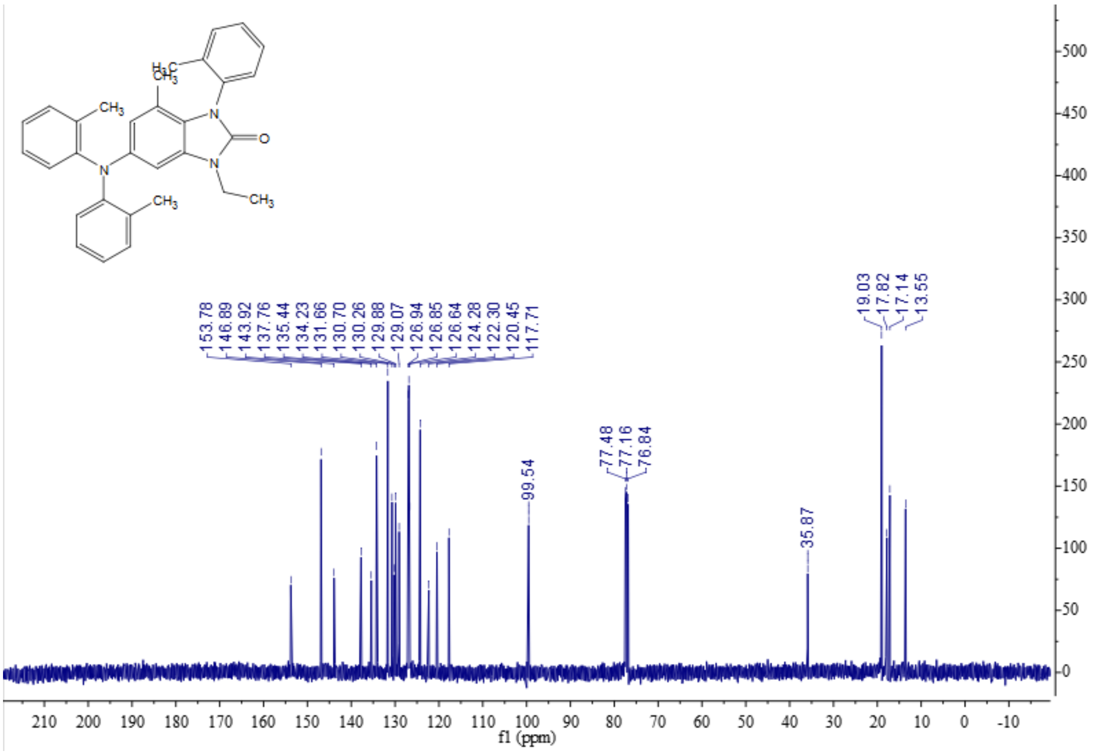

Figure S74. ^{1}H-NMR (400 MHz, $CDCl_3$) spectrum of **5nf**, related to **Scheme 5**.

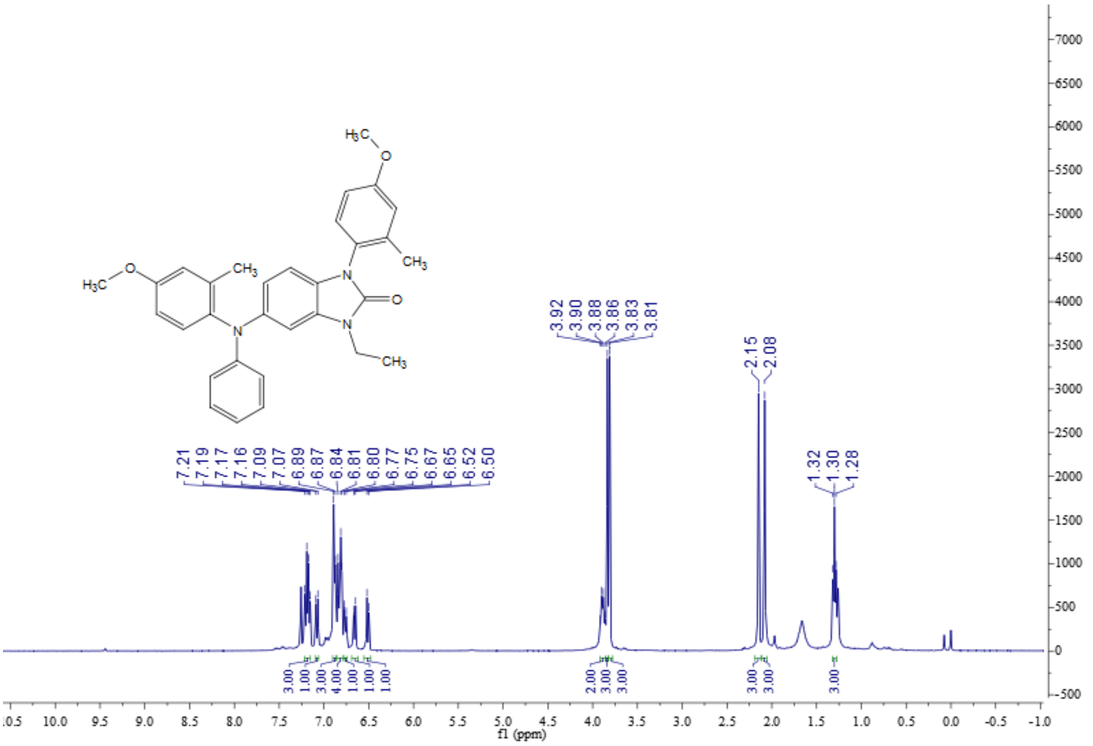

Figure S75. ^{13}C-NMR (100 MHz, $CDCl_3$) spectrum of **5nf**, related to **Scheme 5**.

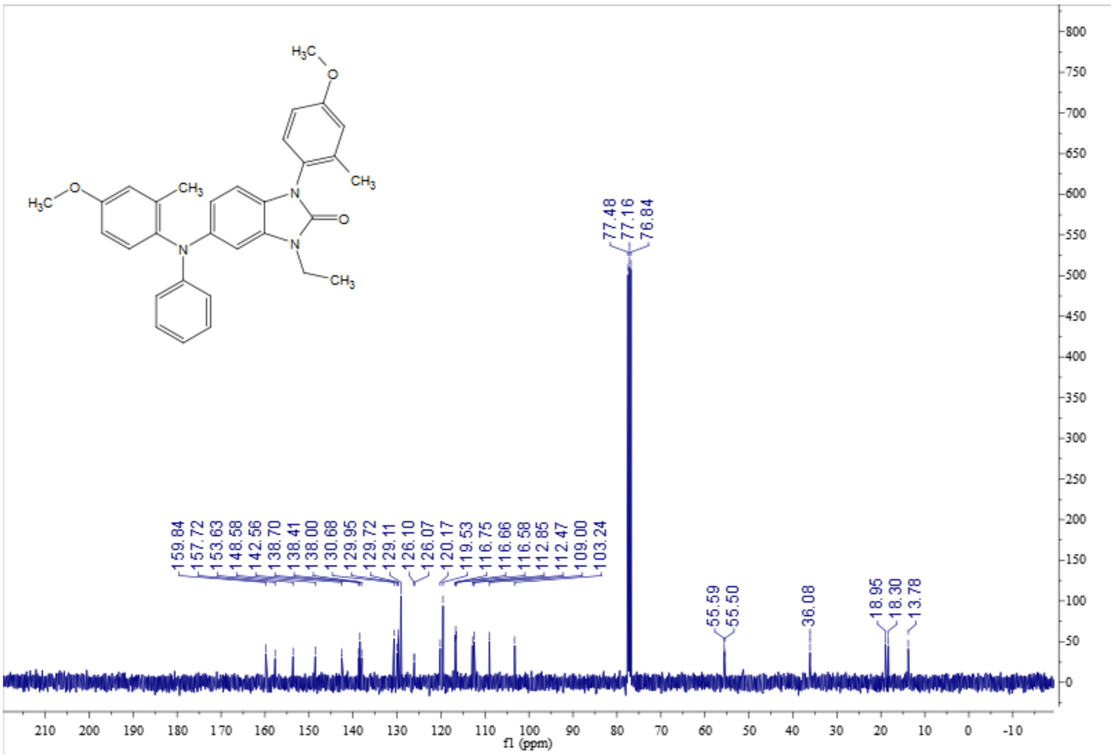

Figure S76. ^{1}H-NMR (400 MHz, $CDCl_3$) spectrum of **5og**, related to **Scheme 5**.

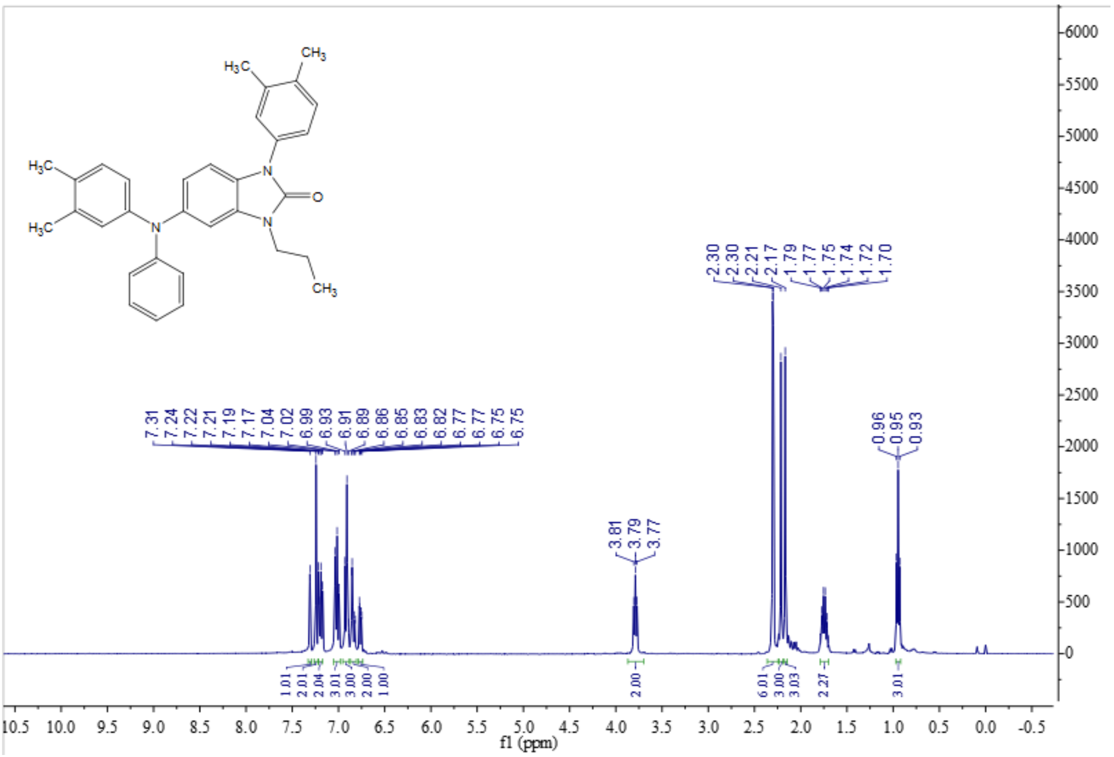

Figure S77. ^{13}C-NMR (100 MHz, $CDCl_3$) spectrum of **5og**, related to **Scheme 5**.

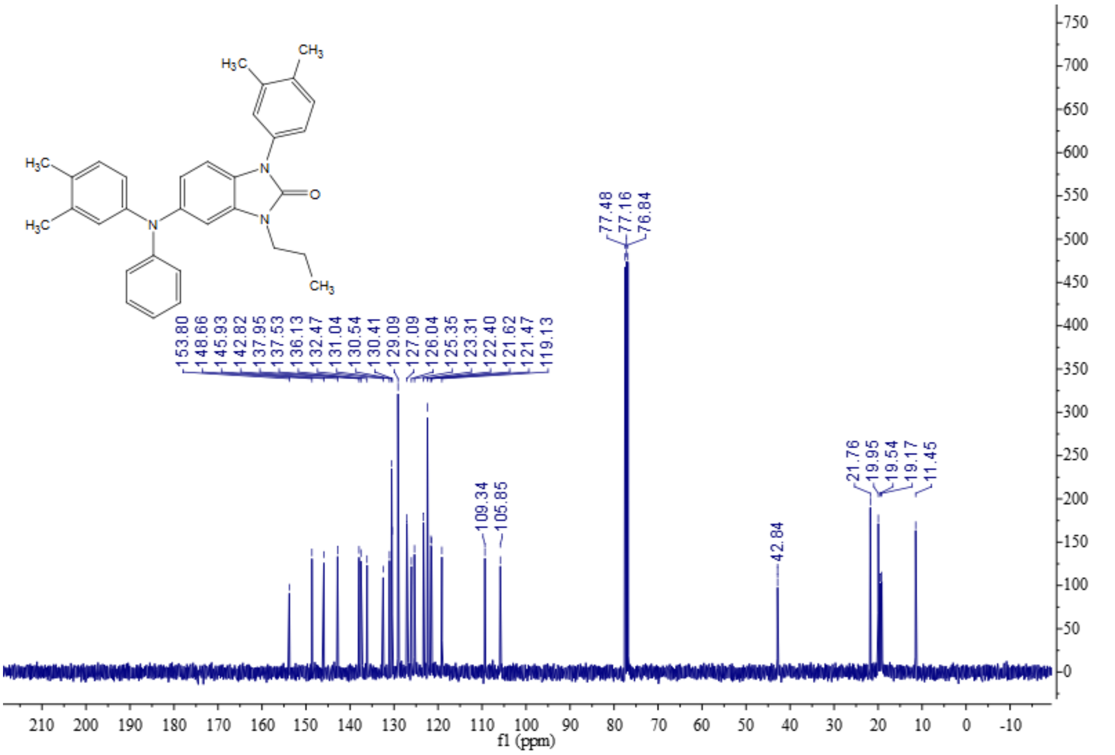

Figure S78. ^{1}H-NMR (400 MHz, $CDCl_3$) spectrum of **5pf**, related to **Scheme 5**.

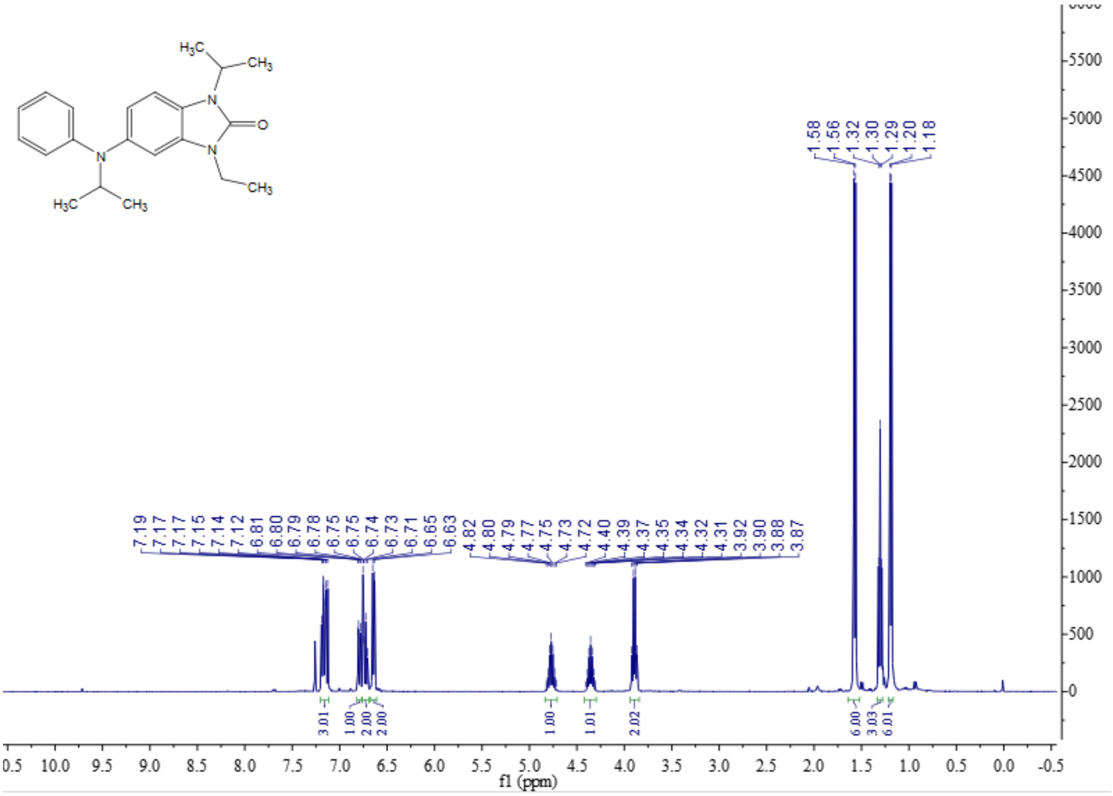

Figure S79. ^{13}C-NMR (100 MHz, $CDCl_3$) spectrum of **5pf**, related to **Scheme 5**.

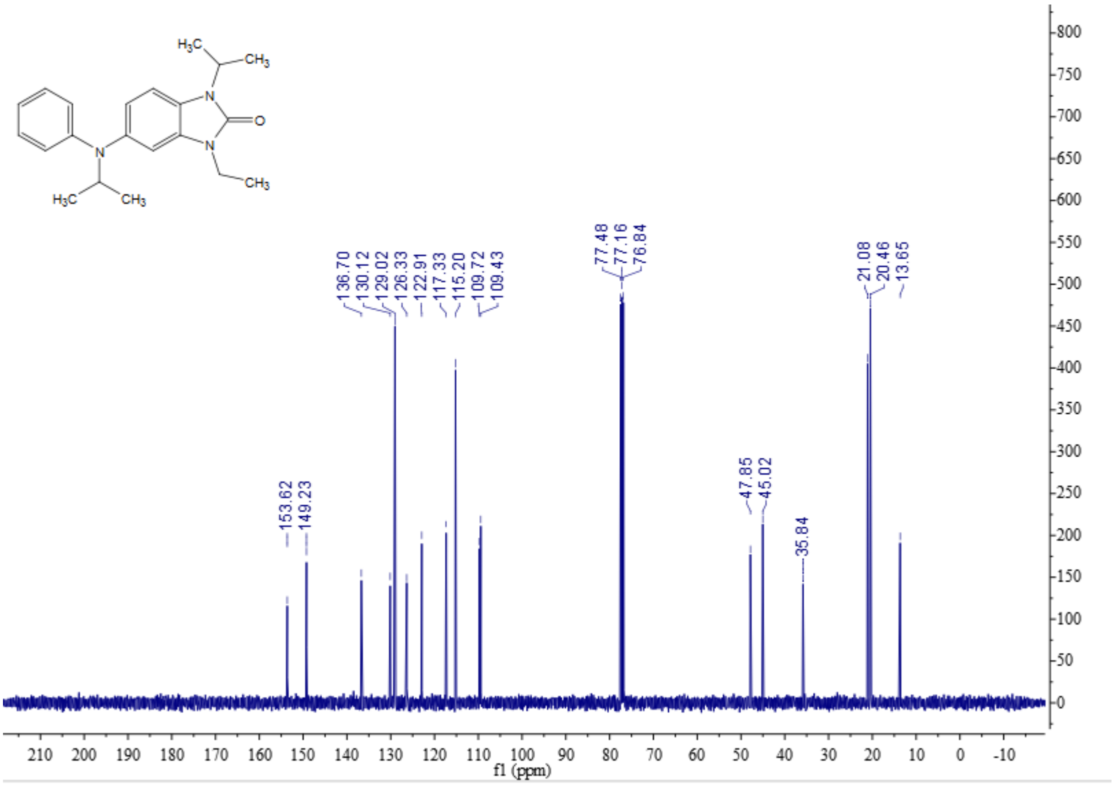

Figure S80. ^{1}H-NMR (400 MHz, $CDCl_3$) spectrum of **1q**, related to **Scheme 6**.

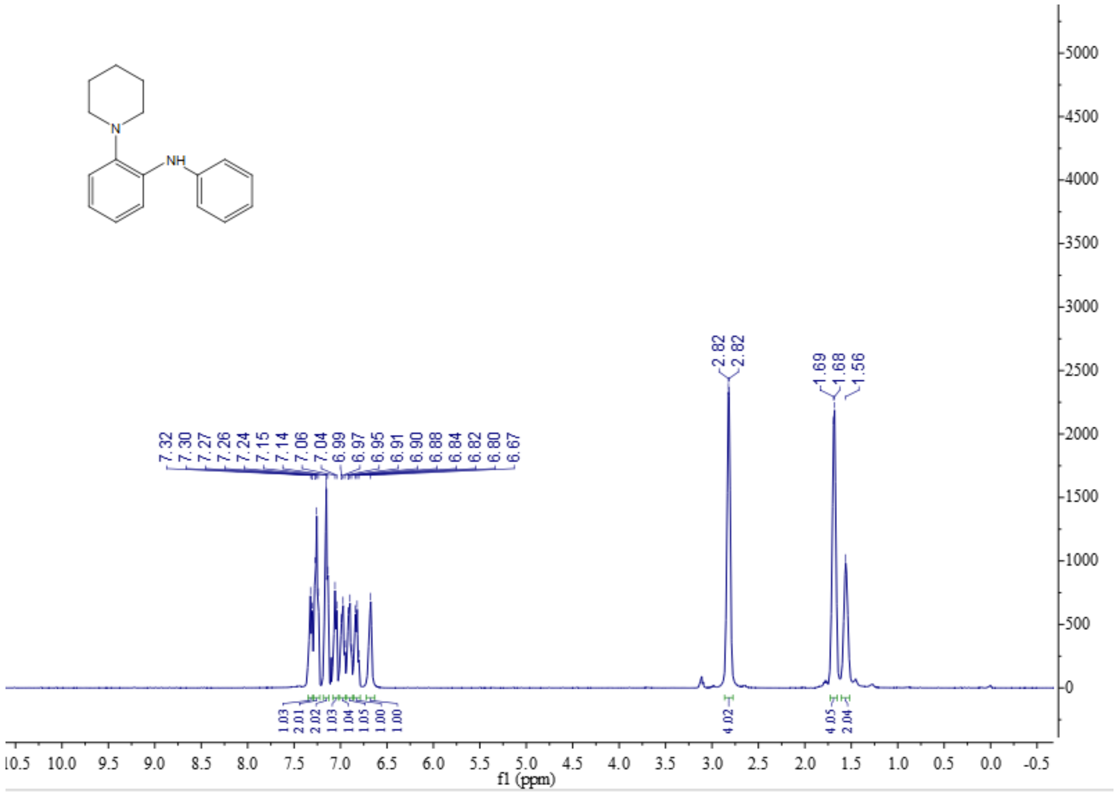

Figure S81. ^{13}C-NMR (100 MHz, $CDCl_3$) spectrum of **1q**, related to **Scheme 6**.

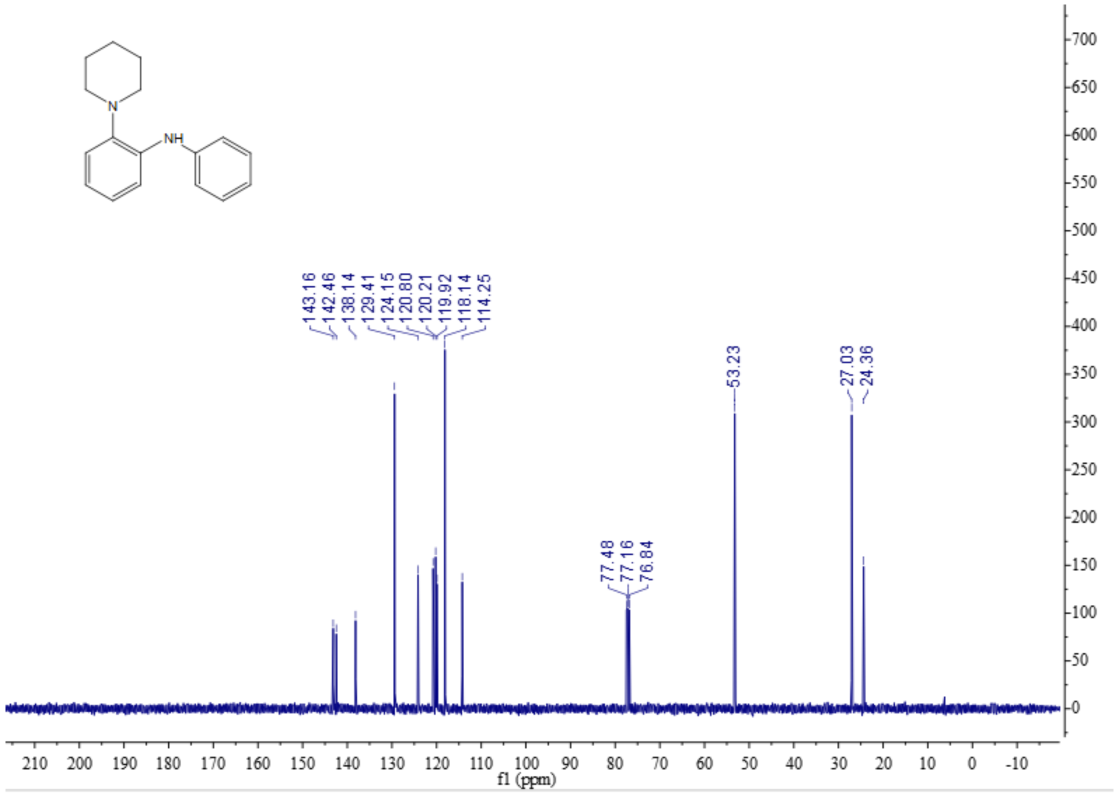

Figure S82. ^{1}H-NMR (400 MHz, $CDCl_3$) spectrum of **6qa**, related to **Scheme 6**.

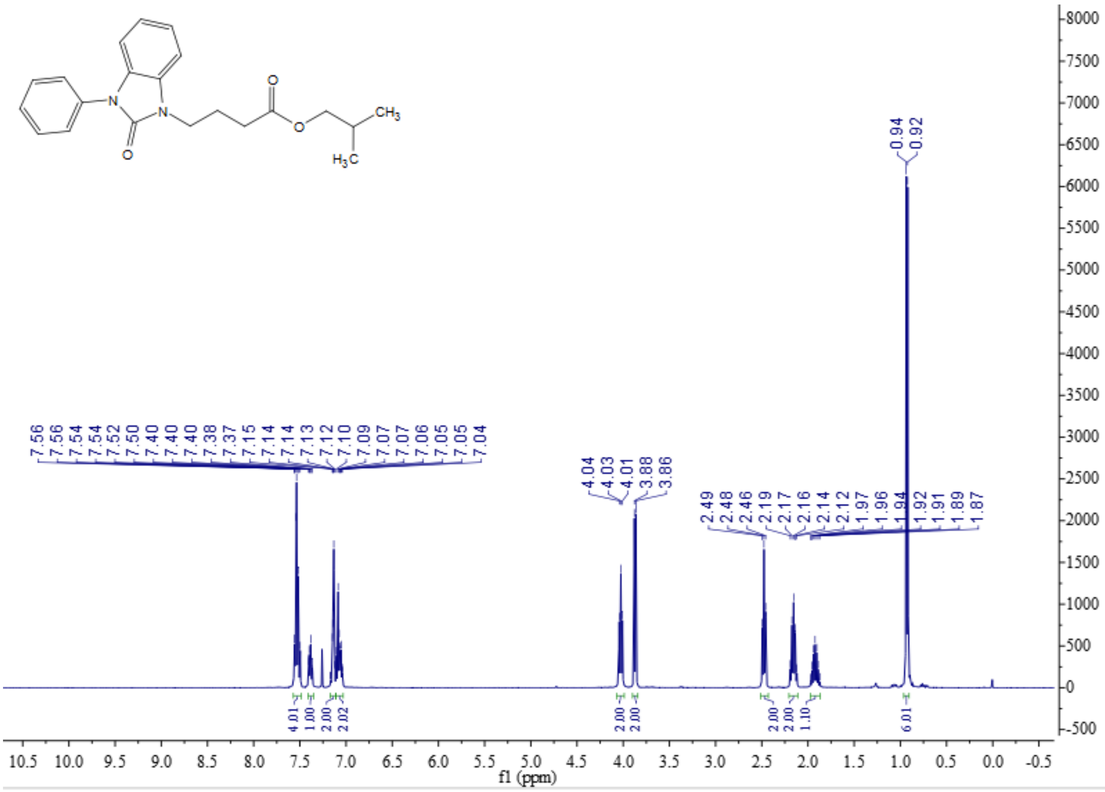

Figure S83. ^{13}C-NMR (100 MHz, $CDCl_3$) spectrum of **6qa**, related to **Scheme 6**.

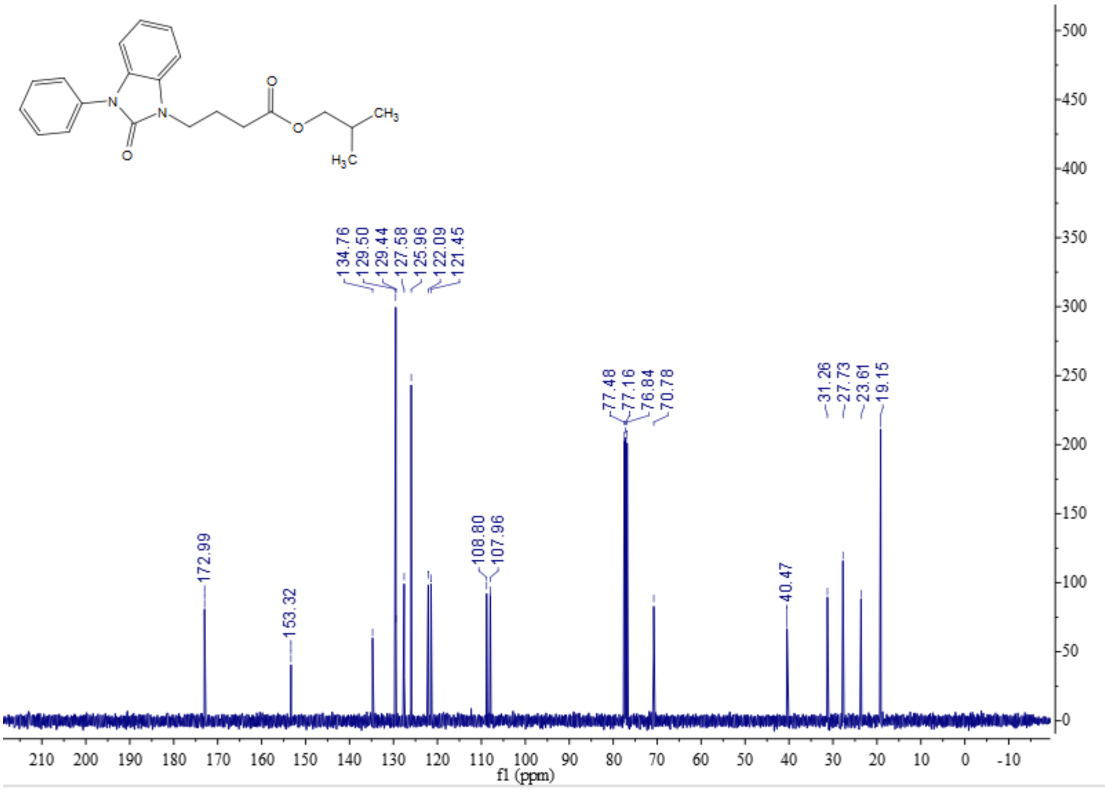

Transparent Methods.

All the obtained products were characterized by melting points (m.p), ^{1}H-NMR, ^{13}C-NMR and infrared spectra (IR). Melting points were measured on an Electrothemal SGW-X4 microscopy digital melting point apparatus and are uncorrected; IR spectra were recorded on a FTLA2000 spectrometer; ^{1}H-NMR and ^{13}C-NMR spectra were obtained on Bruker-400 and referenced to 7.26 ppm for chloroform solvent with TMS as internal standard (0 ppm). Chemical shifts were reported in parts per million (ppm, δ) downfield from tetramethylsilane. Proton coupling patterns are described as singlet (s), doublet (d), triplet (t), multiplet (m); TLC was performed using commercially prepared 100-400 mesh silica gel plates (GF254), and visualization was effected at 254 nm; Unless otherwise stated, all the reagents were purchased from commercial sources (J&K Chemic, TCI, Fluka, Acros, SCRC), used without further purification.

Optimization of reaction conditions.

General procedure for optimization studies. The mixture of diphenylamine **1a** (85 mg, 0.5 mmol), hexamethyleneimine hydrochloride **2a** (34 mg, 0.25 mmol), and catalyst (20 mol %) in *i*-butanol **3a** (1.5 mL) was stirred at 100 °C for 12 h under O_2 atmosphere (using an O_2 balloon). After being cooled to room temperature, the resulting mixture was concentrated by removing the solvent under vacuum, and the residue was purified by preparative TLC on silica (petroleum ether/ethyl acetate = 4/1) to give **4aaa**.

Table S1. Screening of optimal reaction conditions. [a] Related to the first paragraph of "RESULTS AND DISCUSSION" in main text.

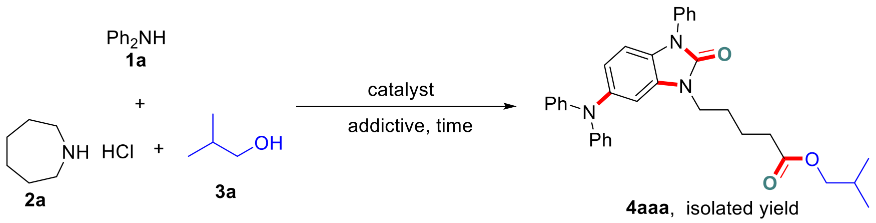

Entry	Catalyst	Additive	Yield% of **4aaa**[b]
1.	CuCl	-	29
2.	$CuCl_2$	-	32
3.	$CuBr_2$	-	trace
4.	$Cu(OAc)_2$	-	nd
5.	$Cu(OTf)_2$	-	19
6.	CuF_2	-	15
7.	$Cu(CH_3CN)_4PF_6$	-	23
8.	CuI	-	nd
9.	$CuCl_2$	pyridine(1.0 eq)	(50, <5, nd)[c]
10.	$CuCl_2$	pyridine(2.0 eq)	(45, 57, 58, 52)[d]
11.	$CuCl_2$	2-phenylpyridine (1.0 eq)	27
12.	$CuCl_2$	4-cyanopyridine (1.0 eq)	25
13.	$CuCl_2$	1,10-phen(1.0 eq)	nd
14.	$CuCl_2$	Ph_3P(1.0 eq)	<5
15.	$CuCl_2$	$Cu(OAc)_2$ (1.0 eq)	25

16.	$CuCl_2$	AgOAc (1.0 eq)	<5
17.	$CuCl_2$	pyridine(2.0 eq) + H_2O_2(2.0 eq)	38
18.	$CuCl_2$	pyridine(2.0 eq) + DCP (2.0 eq)	55
19.	$CuCl_2$	pyridine(2.0 eq) + TBHP(2.0 eq)	42
20.	$CuCl_2$	pyridine(2.0 eq) + DTBP(2.0 eq)	59
21.	$CuCl_2$	pyridine(2.0 eq) + DTBP(1.0 eq)	37
22.	$CuCl_2$	pyridine(2.0 eq) + DTBP(5.0 eq)	45
23.	$CuCl_2$	pyridine(2.0 eq) + NaOH (1.0 eq)	nd
24.	$CuCl_2$	pyridine(2.0 eq) + Na_2CO_3 (1.0 eq)	60
25.	$CuCl_2$	Pyridine(2.0 eq) + Na_2CO_3 (0.2 eq)	43
**26.**	**$CuCl_2$**	**pyridine(2.0 eq) + Na_2CO_3 (2.0 eq)**	**71**
27.	$CuCl_2$	pyridine(2.0 eq) + Na_2CO_3 (5.0 eq)	41
28.	$CuCl_2$	Na_2CO_3 (1.0 eq)	49
29.	$CuCl_2$	pyridine(2.0 eq) + $NaHCO_3$ (1.0 eq)	56
30.	$CuCl_2$	pyridine(2.0 eq) + K_2CO_3 (1.0 eq)	50
31.	$CuCl_2$	pyridine(2.0 eq) + NaH (1.0 eq)	39
32.	$CuCl_2$	pyridine(2.0 eq) + *t*-BuONa (1.0 eq)	trace
33.	$CuCl_2$	pyridine(2.0 eq) + *t*-BuOK (1.0 eq)	trace
34.	$CuCl_2$	pyridine(2.0 eq) + $CsCO_3$ (1.0 eq)	trace
35.	$CuCl_2$	pyridine(2.0 eq) + CH_3ONa (1.0 eq)	trace
36.	$CuCl_2$	pyridine(2.0 eq) + K_3PO_4 (1.0 eq)	32
37.	$CuCl_2$	pyridine(2.0 eq) + $NaH_2PO_2{\cdot}H_2O$ (1.0 eq)	32
38.	$CuCl_2$	pyridine(2.0 eq) + KPF_6 (1.0 eq)	47
39.	$CuCl_2$	pyridine(2.0 eq) + alanine (1.0 eq)	20
40.	$CuCl_2$	pyridine(2.0 eq) + citric Acid (1.0 eq)	trace
41.	$CuCl_2$	pyridine(2.0 eq) + HBF_4 (1.0 eq)	20

[a] Reation conditions: Unless otherwise stated, all the reactions were performed with **1a** (0.50 mmol), **2a** (0.25 mmol), **3a** (1.5 mL) Catalyst (20 mol %) at 100 ^{o}C for 12 h under O_2 atmosphere (by using an O_2 ballroom). [b] Isolated yield. [c] Yields are with respect to under O_2 atmosphere, under air atmosphere and under N_2 atmosphere, respectively. [d] Yields are with respect to 8h, 12h, 16h and 24h, respectively.

Typical procedure for the synthesis of 4aaa.

The mixture of diphenylamine **1a** (85mg, 0.5 mmol), hexamethyleneimine hydrochloride **2a** (34 mg, 0.25 mmol), $CuCl_2$ (7 mg, 0.05 mmol), Na_2CO_3 (53 mg, 0.5 mmol) and pyridine (40 mg, 0.5 mmol) in *i*-butanol **3a** (1.5 mL) was stirred at 100 °C for 12 h under O_2 atmosphere (using an O_2 balloon). After being cooled to room temperature, the resulting mixture was concentrated by removing the solvent under vacuum, and the residue was purified by preparative TLC on silica (petroleum ether/ethyl acetate = 4/1) to give isobutyl-5-(6-(diphenylamino)-2-oxo-3-phenyl-2,3-dihydro-1*H*-benzo[*d*]imidazol-1-yl)pentanoate **4aaa**.

Scheme S1. Substrates employed for synthesizing **4** and **5**. Related to **Scheme 3, 4** & **5**.

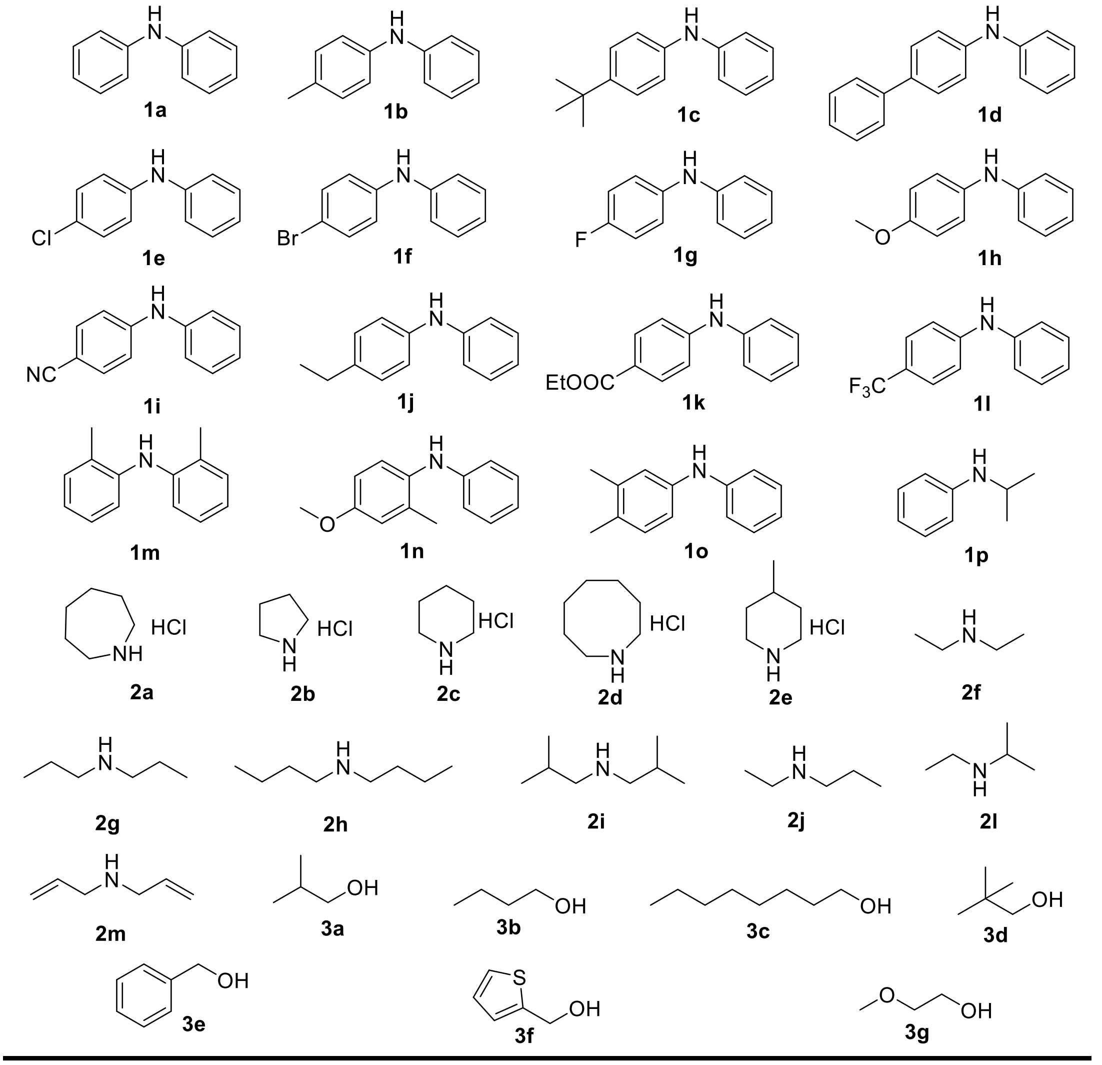

The control Experiments.

(1) The preparation of **1aa** was similar to the literature procedures. (Yang et al., 2012) A mixture of N[1],N[4]-diphenylphenylenediamine (3905 mg, 15 mmol), bromobenzene (785 mg, 5 mmol), $Pd_2(dba)_3$ (27 mg, 0.03 mmol), DPPF (34 mg, 0.06 mmol), and *t*-BuONa (1440 mg, 15 mmol) in toluene (10 mL) was refluxed under N_2 atmosphere for 21 h. The reaction mixture was then filtered. The filtrate was evaporated under vacuum to remove the solvent and the crude product was then purified by column chromatography on silica gel eluting with dichloromethane/hexane (1:2), which afforded compounds **1aa** as a white solid (605 mg, 36%).

The analytic data of compound **1aa**: ^1H NMR (400 MHz, DMSO) δ 8.14 (s, 1H), 7.26 – 7.18 (m, 6H), 7.03 – 7.11 (m, 4H), 6.92 – 6.99 (m, 8H), 6.79 (t, *J* = 7.2 Hz, 1H).

Under the optimized reaction conditions, the reaction of **1aa** (84 mg, 0.25 mmol) and **2a** (34 mg, 0.25 mmol) was carried out for 12 h. Then, the reaction mixture was purified by preparative TLC on silica eluting with petroleum ether/ethyl acetate (4:1) to give product **4aaa** as a brownish oid (109 mg, 82% yield).

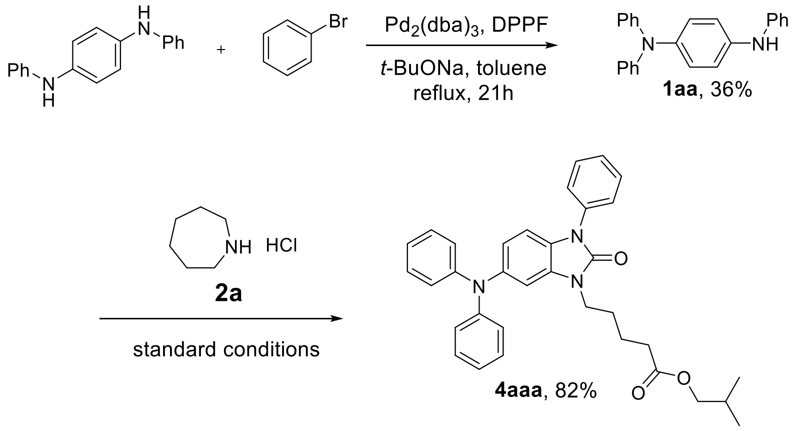

Figure S84. Related to **Scheme 6**.

(2) Under the optimized reaction conditions, the model reaction was carried out for 12 hours by introducing 3.0 equivalent of TEMPO (2,2,6,6-tetramethyl-1-piperidinyloxy), and the crude reaction mixture was analyzed by TLC and GC-MS, which indicated that no **4aaa** was formed during the reaction.

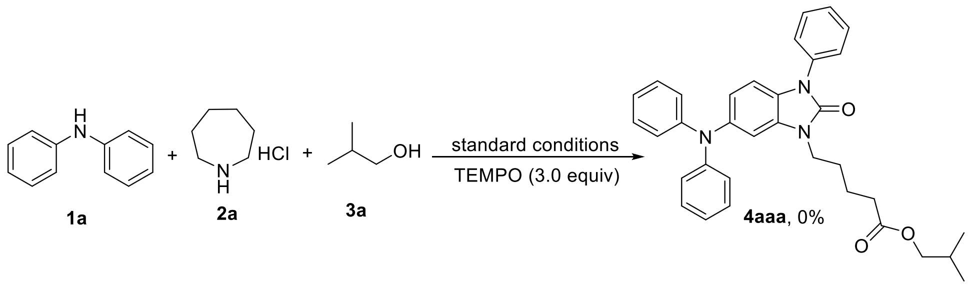

Figure S85. Related to **Scheme 6**.

(3) The preparation of N-phenyl-2-(piperidin-1-yl)aniline **1q** was similar to literature procedures. (Shi et al., 2013) General procedure: a mixture of fresh aniline **1q'** (921 mg, 5.0 mmol), 1,5-diiodopentane (1620 mg, 5.0 mmol), and K_2CO_3 (1382 mg, 10 mmol) in EtOH (10 mL) was refluxed at 75 ^oC for 18 h. The suspension was filtered, and the resulting solid was washed

with CH_2Cl_2. The filtered solution was extracted with water, and the organic layer was dried over anhydrous $MgSO_4$ and concentrated in vacuo. Purification by column chromatography on silica gel (eluent: $CHCl_3$) produced an oil.

The analytic data of compound (**1q**): Brownish liquid, (1046 mg, 83% yield); ^{1}H NMR (400 MHz, $CDCl_3$) δ 7.31 (d, *J* = 7.6 Hz, 1H), 7.26 (t, *J* = 6.8 Hz, 2H), 7.14 (d, *J* = 7.2 Hz, 2H), 7.05 (d, *J* = 7.4 Hz, 1H), 6.97 (t, *J* = 7.4 Hz, 1H), 6.90 (t, *J* = 7.0 Hz, 1H), 6.82 (t, *J* = 7.2 Hz, 1H), 6.67 (s, 1H), 2.82 (d, *J* = 2.8 Hz, 4H), 1.69 (d, *J* = 4.0 Hz, 4H), 1.56 (s, 2H). ^{13}C NMR (100 MHz, $CDCl_3$) δ 143.16, 142.46, 138.14, 129.41, 124.15, 120.80, 120.21, 119.92, 118.14, 114.25, 53.23, 27.03, 24.36. IR (KBr): 3042, 2934, 2850, 2807, 1714, 1591, 1512, 1462, 1418, 1314, 1225, 788, 744 cm^{-1}. MS (EI, m/z): 252 [M]$^+$. HRMS (ESI): Calcd. for $C_{17}H_{21}N_2$ [M+H]$^+$: 253.1699; found: 253.1701.

Then, under the standard conditions, the reaction of **1q** (63 mg, 0.25 mmol) add an equivalent of **1a** was carried out, and the reaction mixture was analyzed by TLC, after the reaction finished completely. Then being cooled to room temperature, the resulting mixture was concentrated by removing the solvent under vacuum, and the reaction mixture was purified by preparative TLC on silica gel eluting with petroleum ether / ethyl acetate (20:1) to give product **6qa** as a brownish solid.

The analytic data of compound (**6qa**): Brownish oil liquid, (69 mg, 78% yield); ^{1}H NMR (400 MHz, $CDCl_3$): δ 7.57 – 7.49 (m, 4H), 7.41 – 7.35 (m, 1H), 7.17 – 7.11 (m, 2H), 7.11 – 7.03 (m, 2H), 4.03 (t, *J* = 7.0 Hz, 2H), 3.87 (d, *J* = 6.8 Hz, 2H), 2.48 (t, *J* = 7.2 Hz, 2H), 2.16 (p, *J* = 7.2 Hz, 2H), 1.98 – 1.87 (m, 1H), 0.93 (d, *J* = 6.8 Hz, 6H). ^{13}C NMR (100 MHz, $CDCl_3$): δ 172.99, 153.32, 134.76, 129.50, 129.44, 127.58, 125.96, 122.09, 121.45, 108.80, 107.96, 70.78, 40.47, 31.26, 27.73, 23.61, 19.15. IR (KBr): 3063, 2961, 2875, 2831, 1715, 1598, 1502, 1173, 753, 734, 697 cm^{-1}. MS (EI, m/z): 352 [M]$^+$. HRMS (ESI): Calcd. for $C_{21}H_{25}N_2O_3$ [M+H]$^+$: 353.1860; found: 353.1864.

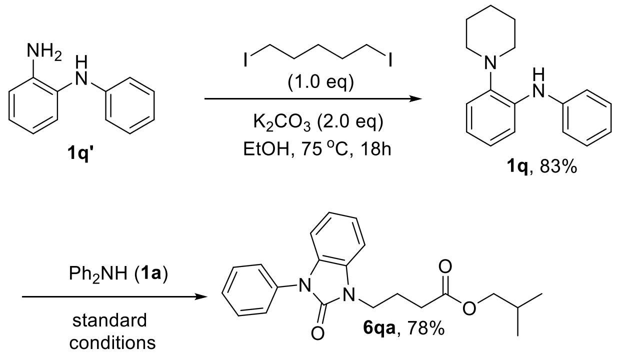

Figure S86. Related to **Scheme 6**.

(4) Under the optimized reaction conditions, the reaction of diarylamine **1r** and dialkylamine **2f** was carried out at 100 ^{o}C for 12 hours, and the crude reaction mixture was analyzed by TLC and GC-MS, which indicated that no **5rf** was formed during the reaction.

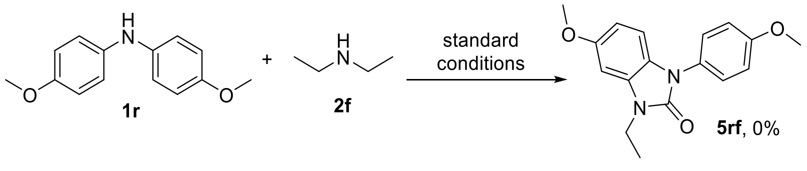

Figure S87. Related to **Scheme 6**.

Single crystal X-ray diffraction of 5al.

Yellow block-like single crystals of **5al** were grown by layering a dichlormethane solution with *n*-hexane at ambient temperature. X-Ray diffraction data of one these crystals were collected on a R-AXIS SPIDER diffractometer. The measurements were performed with Mo-Kα radiation (λ = 0.71073 Å). Data were collected at 296(2) K, using the *ω*- and *φ*- scans to a maximum θ value of 25.03°. The data were refined by full-matrix least-squares techniques on F^2 with SHELXTL-2014. And the structures were solved by direct methods SHELXS-2014. All the non-hydrogen atoms were refined anisotropically. The hydrogen atoms were included at geometrically idealized positions. An ORTEP representation of the structure is shown below.

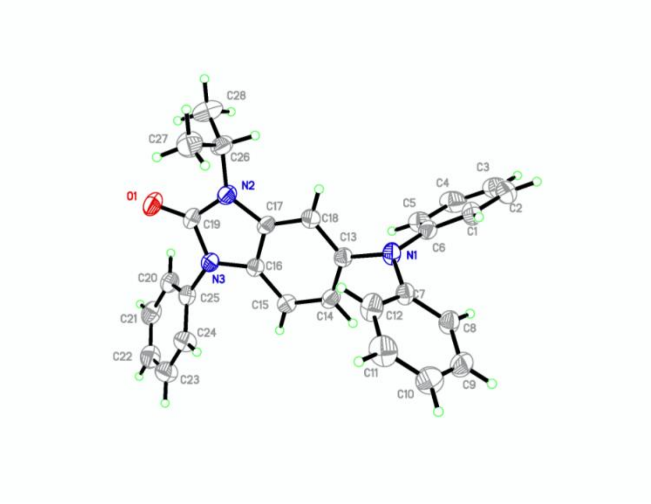

Figure S88. ORTEP drawing of **5al** with the numbering scheme. Related to **Scheme 4**.

Table S2. Crystal data and structure refinement for **5al**. Related to **Scheme 4**.

Identification code	5al	
Empirical formula	$C_{28}H_{25}N_3O$	
Formula weight	419.51	
Temperature	296(2) K	
Wavelength	0.71073 Å	
Crystal system	Monoclinic	
space group	$P2_1/n$	
Unit cell dimensions	a = 7.9689(6) Å	alpha = 90°
	b = 11.6429(9) Å	beta = 97.043(2) °
	c = 24.549(2) Å	gamma = 90°
Volume	2260.5(3) $Å^3$	
Z	4	
Calculated density	1.233 Mg/m^3	
Absorption coefficient	0.076 mm^{-1}	
F(000)	888	
Crystal size	0.23 x 0.20 x 0.18 mm^3	
Theta range for data collection	2.61 to 25.03°.	
Limiting indices	-9<=h<=6, -13<=k<=13, -29<=l<=27	
Reflections collected / unique	14344 / 4002 [R(int) = 0.0486]	

Completeness to theta = 25.03	99.9 %
Absorption correction	None
Max. and min. transmission	0.9865 and 0.9828
Refinement method	Full-matrix least-squares on F^2
Data / restraints / parameters	4002 / 0 / 290
Goodness-of-fit on F^2	1.018
Final R indices [I>2sigma(I)]	R1 = 0.0494, wR2 = 0.1162
R indices (all data)	R1 = 0.0910, wR2 = 0.1392
Extinction coefficient	0.0068(12)
Largest diff. peak and hole	0.192 and -0.147 e. Å $^{-3}$

Table S3. Atomic coordinates (x 10^4) and equivalent isotropic displacement parameters (Å ^{2} x 10^3) for **5al**. U(eq) is defined as one third of the trace of the orthogonalized U^{ij} tensor. Related to **Scheme 4**.

	**x**	**y**	**z**	**U(eq)**
O(1)	9782(2)	2008(2)	2364(1)	68(1)
N(1)	5689(2)	3364(2)	-310(1)	52(1)
N(2)	7809(2)	2646(2)	1645(1)	52(1)
N(3)	10022(2)	1639(2)	1444(1)	47(1)
C(1)	3955(3)	1630(2)	-345(1)	62(1)
C(2)	2493(4)	1057(3)	-530(1)	82(1)
C(3)	1222(4)	1595(3)	-858(1)	89(1)
C(4)	1392(3)	2736(3)	-989(1)	84(1)
C(5)	2861(3)	3323(3)	-801(1)	67(1)
C(6)	4176(3)	2770(2)	-487(1)	50(1)
C(7)	6488(3)	4043(2)	-687(1)	48(1)
C(8)	6240(3)	3855(2)	-1248(1)	59(1)
C(9)	7084(3)	4515(2)	-1594(1)	74(1)
C(10)	8177(4)	5354(3)	-1391(1)	85(1)
C(11)	8433(4)	5542(2)	-834(1)	84(1)
C(12)	7596(3)	4899(2)	-482(1)	67(1)
C(13)	6826(3)	2872(2)	129(1)	46(1)
C(14)	8174(3)	2214(2)	7(1)	52(1)
C(15)	9330(3)	1742(2)	413(1)	49(1)
C(16)	9084(3)	1954(2)	948(1)	43(1)
C(17)	7712(3)	2597(2)	1076(1)	44(1)
C(18)	6558(3)	3070(2)	669(1)	47(1)
C(19)	9245(3)	2090(2)	1877(1)	51(1)
C(20)	11657(3)	18(2)	1844(1)	57(1)
C(21)	13073(4)	-673(2)	1875(1)	71(1)
C(22)	14328(3)	-439(2)	1556(1)	74(1)
C(23)	14205(3)	504(2)	1220(1)	73(1)
C(24)	12813(3)	1212(2)	1191(1)	63(1)
C(25)	11521(3)	956(2)	1502(1)	48(1)

C(26)	6652(3)	3298(2)	1954(1)	62(1)
C(27)	7417(3)	4429(2)	2142(1)	80(1)
C(28)	6001(4)	2587(3)	2389(1)	94(1)

Table S4. Bond lengths [Å] and angles [°] for **5al**. Related to **Scheme 4**.

O(1)-C(19)	1.223(3)
N(1)-C(6)	1.412(3)
N(1)-C(13)	1.440(3)
N(1)-C(7)	1.426(3)
N(2)-C(19)	1.376(3)
N(2)-C(17)	1.389(3)
N(2)-C(26)	1.474(3)
N(3)-C(19)	1.398(3)
N(3)-C(16)	1.396(3)
N(3)-C(25)	1.428(3)
C(1)-C(2)	1.370(3)
C(1)-C(6)	1.390(3)
C(2)-C(3)	1.366(4)
C(3)-C(4)	1.377(4)
C(4)-C(5)	1.385(4)
C(5)-C(6)	1.381(3)
C(7)-C(12)	1.385(3)
C(7)-C(8)	1.385(3)
C(8)-C(9)	1.379(3)
C(9)-C(10)	1.362(4)
C(10)-C(11)	1.374(4)
C(11)-C(12)	1.376(3)
C(13)-C(14)	1.382(3)
C(13)-C(18)	1.387(3)
C(14)-C(15)	1.386(3)
C(15)-C(16)	1.374(3)
C(16)-C(17)	1.393(3)
C(17)-C(18)	1.387(3)
C(20)-C(25)	1.375(3)
C(20)-C(21)	1.380(3)
C(21)-C(22)	1.371(3)
C(22)-C(23)	1.370(4)
C(23)-C(24)	1.377(3)
C(24)-C(25)	1.387(3)
C(26)-C(27)	1.499(3)
C(26)-C(28)	1.495(3)
C(6)-N(1)-C(13)	118.11(17)
C(6)-N(1)-C(7)	120.30(18)

C(13)-N(1)-C(7)	114.68(17)
C(19)-N(2)-C(17)	109.80(17)
C(19)-N(2)-C(26)	124.7(2)
C(17)-N(2)-C(26)	125.19(19)
C(19)-N(3)-C(16)	108.99(18)
C(19)-N(3)-C(25)	125.23(19)
C(16)-N(3)-C(25)	125.77(17)
C(2)-C(1)-C(6)	120.7(3)
C(3)-C(2)-C(1)	120.7(3)
C(2)-C(3)-C(4)	119.5(3)
C(5)-C(4)-C(3)	120.1(3)
C(6)-C(5)-C(4)	120.5(3)
C(5)-C(6)-C(1)	118.4(2)
C(5)-C(6)-N(1)	120.3(2)
C(1)-C(6)-N(1)	121.3(2)
C(12)-C(7)-C(8)	118.9(2)
C(12)-C(7)-N(1)	118.6(2)
C(8)-C(7)-N(1)	122.5(2)
C(9)-C(8)-C(7)	120.1(2)
C(8)-C(9)-C(10)	120.9(3)
C(9)-C(10)-C(11)	119.2(2)
C(12)-C(11)-C(10)	120.9(3)
C(7)-C(12)-C(11)	119.9(3)
C(14)-C(13)-C(18)	121.0(2)
C(14)-C(13)-N(1)	119.4(2)
C(18)-C(13)-N(1)	119.56(19)
C(13)-C(14)-C(15)	122.0(2)
C(16)-C(15)-C(14)	117.2(2)
C(15)-C(16)-C(17)	121.4(2)
C(15)-C(16)-N(3)	131.5(2)
C(17)-C(16)-N(3)	107.14(18)
C(16)-C(17)-C(18)	121.3(2)
C(16)-C(17)-N(2)	107.48(19)
C(18)-C(17)-N(2)	131.20(19)
C(17)-C(18)-C(13)	117.2(2)
O(1)-C(19)-N(2)	128.0(2)
O(1)-C(19)-N(3)	125.4(2)
N(2)-C(19)-N(3)	106.51(19)
C(25)-C(20)-C(21)	120.0(2)
C(22)-C(21)-C(20)	120.1(2)
C(21)-C(22)-C(23)	120.0(3)
C(24)-C(23)-C(22)	120.5(2)
C(25)-C(24)-C(23)	119.4(2)
C(20)-C(25)-C(24)	119.9(2)

C(20)-C(25)-N(3)	120.38(19)
C(24)-C(25)-N(3)	119.62(19)
N(2)-C(26)-C(27)	110.78(19)
N(2)-C(26)-C(28)	112.1(2)
C(27)-C(26)-C(28)	115.5(2)

Symmetry transformations used to generate equivalent atoms:

Table S5. Anisotropic displacement parameters ($Å^2 \times 10^3$) for **5al**. The anisotropic displacement factor exponent takes the form: $-2 pi^2 [h^2 a^{*2} U^{11} + ... + 2 h k a^* b^* U^{12}]$. Related to **Scheme 4**.

	U^{11}	U^{22}	U^{33}	U^{23}	U^{13}	U^{12}
O(1)	85(1)	77(1)	40(1)	-3(1)	-1(1)	9(1)
N(1)	52(1)	56(1)	45(1)	9(1)	-3(1)	-14(1)
N(2)	57(1)	56(1)	42(1)	0(1)	7(1)	6(1)
N(3)	52(1)	47(1)	42(1)	3(1)	5(1)	1(1)
C(1)	64(2)	54(2)	71(2)	-7(1)	16(1)	-10(1)
C(2)	72(2)	76(2)	102(3)	-24(2)	28(2)	-27(2)
C(3)	61(2)	121(3)	86(2)	-44(2)	20(2)	-35(2)
C(4)	51(2)	130(3)	68(2)	-9(2)	-1(1)	-8(2)
C(5)	55(2)	84(2)	59(2)	6(1)	-2(1)	-5(1)
C(6)	49(1)	57(2)	43(1)	-4(1)	6(1)	-8(1)
C(7)	52(1)	44(1)	46(2)	7(1)	-1(1)	-7(1)
C(8)	62(2)	61(2)	52(2)	6(1)	1(1)	-12(1)
C(9)	75(2)	89(2)	56(2)	21(2)	6(1)	-11(2)
C(10)	84(2)	86(2)	84(3)	38(2)	12(2)	-18(2)
C(11)	90(2)	60(2)	100(3)	16(2)	1(2)	-35(2)
C(12)	81(2)	54(2)	64(2)	2(1)	-3(1)	-19(1)
C(13)	49(1)	48(1)	41(1)	6(1)	4(1)	-8(1)
C(14)	61(2)	57(1)	38(1)	3(1)	9(1)	-7(1)
C(15)	53(1)	50(1)	47(2)	1(1)	9(1)	0(1)
C(16)	49(1)	39(1)	41(1)	6(1)	6(1)	-6(1)
C(17)	53(1)	44(1)	35(1)	0(1)	9(1)	-7(1)
C(18)	48(1)	45(1)	48(2)	2(1)	7(1)	-3(1)
C(19)	64(2)	52(1)	36(2)	-1(1)	2(1)	-5(1)
C(20)	70(2)	53(1)	48(2)	4(1)	3(1)	1(1)
C(21)	91(2)	62(2)	56(2)	8(1)	-3(2)	17(2)
C(22)	76(2)	74(2)	70(2)	-9(2)	-4(2)	23(2)
C(23)	63(2)	78(2)	80(2)	1(2)	14(1)	5(2)
C(24)	62(2)	54(2)	74(2)	8(1)	13(1)	1(1)
C(25)	55(1)	43(1)	45(1)	-1(1)	0(1)	-1(1)
C(26)	70(2)	68(2)	50(2)	-7(1)	16(1)	10(1)
C(27)	89(2)	66(2)	86(2)	-18(2)	10(2)	11(2)
C(28)	116(2)	93(2)	82(2)	-1(2)	51(2)	7(2)

Table S6. Hydrogen coordinates (x 10^4) and isotropic displacement parameters (Å ^{2} x 10^3) for **5al**. Related to **Scheme 4**.

	x	y	z	U(eq)
H(1A)	4808	1251	-122	75
H(2A)	2364	295	-430	98
H(3A)	250	1194	-992	106
H(4A)	519	3112	-1204	101
H(5A)	2962	4097	-887	80
H(8A)	5502	3282	-1393	70
H(9A)	6906	4386	-1970	88
H(10A)	8743	5796	-1626	102
H(11A)	9182	6111	-693	101
H(12A)	7775	5039	-106	81
H(14A)	8309	2085	-359	62
H(15A)	10234	1301	327	59
H(18A)	5642	3501	754	56
H(20A)	10795	-151	2055	69
H(21A)	13176	-1298	2113	85
H(22A)	15263	-920	1568	89
H(23A)	15069	667	1009	88
H(24A)	12739	1856	965	76
H(26A)	5666	3483	1689	74
H(27A)	7767	4832	1834	120
H(27B)	8379	4299	2410	120
H(27C)	6594	4879	2302	120
H(28A)	5518	1890	2229	141
H(28B)	5151	3010	2549	141
H(28C)	6915	2403	2668	141

Analytic data of the obtained compounds.

(1)isobutyl-5-(6-(diphenylamino)-2-oxo-3-phenyl-2,3-dihydro-1*H*-benzo[*d*]imidazol-1-yl)pentanoate (4aaa)

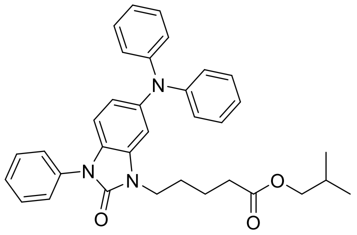

Brownish oil liquid, (95 mg, 71% yield); ^{1}H NMR (400 MHz, $CDCl_3$): δ 7.56 – 7.46 (m, 4H), 7.35 (t, *J* = 7.2 Hz, 1H), 7.25 – 7.19 (m, 4H), 7.08 (d, *J* = 7.6 Hz, 4H), 7.00 – 6.94 (m, 3H), 6.86 (d, *J* =2.0 Hz, 1H), 6.80 (dd, *J* = 8.4, 1.6 Hz, 1H), 3.89 – 3.79 (m, 4H), 2.35 (t, *J* = 7.0 Hz, 2H), 1.94 – 1.84 (m, 1H), 1.81 – 1.73 (m, 2H), 1.73 – 1.65 (m, 2H), 0.90 (d, *J* = 6.8 Hz, 6H). ^{13}C NMR (100 MHz, $CDCl_3$): δ 173.19, 153.48, 148.12, 142.80, 134.79, 130.40, 129.46, 129.19, 127.43, 125.79, 125.75, 123.19, 122.25, 119.43, 109.45, 105.96, 70.48, 40.75, 33.79, 27.76, 27.70, 22.23, 19.12. IR (KBr): 3062, 2959, 2872, 1715, 1594, 1491, 1399, 1274, 1173, 754, 695, 657 cm^{-1}. MS (EI, m/z): 533 $[M]^+$. HRMS (ESI): Calcd. for $C_{34}H_{35}N_3NaO_3$ $[M+H]^+$: 556.2571; found: 556.2579.

(2)isobutyl-5-(2-oxo-6-(phenyl(p-tolyl)amino)-3-(*p*-tolyl)-2,3-dihydro-1*H*-benzo[*d*]imidazol-1-yl)pentanoate (4baa)

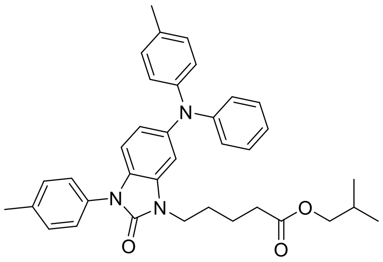

Brownish oil liquid, (107 mg, 76% yield); ^{1}H NMR (400 MHz, $CDCl_3$): δ 7.42 (d, *J* = 8.4 Hz, 2H), 7.32 (d, *J* = 8.4 Hz, 2H), 7.22 (t, *J* = 7.8 Hz, 2H), 7.04 (s, 3H), 7.01 (d, *J* = 8.4 Hz, 2H), 6.97 – 6.93 (m, 2H), 6.85 (d, *J* = 1.6 Hz, 1H), 6.79 (dd, *J* = 8.4, 1.6 Hz, 1H), 3.95 – 3.74 (m, 4H), 2.42 (s, 3H), 2.36 (t, *J* = 7.2 Hz, 2H), 2.33 (s, 3H), 1.96 – 1.86 (m, 1H), 1.83 – 1.66 (m, 4H), 0.92 (d, *J* = 6.8 Hz, 6H). ^{13}C NMR (100 MHz, $CDCl_3$): δ 173.32, 153.70, 148.53, 145.65, 142.98, 137.45, 132.31, 132.21, 130.37, 130.14, 129.96, 129.16, 125.91, 125.77, 124.07, 122.53, 121.72, 119.16, 109.42, 105.68, 70.57, 40.82, 33.89, 27.87, 27.79, 22.32, 21.25, 20.88, 19.18. IR (KBr): 3031, 2925, 1715, 1593, 1492, 1400, 1268, 1108, 811, 747, 652 cm^{-1}. MS (EI, m/z): 561 $[M]^+$. HRMS (ESI): Calcd. for $C_{36}H_{39}N_3NaO_3$ $[M+H]^+$: 584.2884; found: 584.2892.

(3)isobutyl-5-(3-(4-(tert-butyl)phenyl)-6-((4-(tert-butyl)phenyl)(phenyl)amino)-2-oxo-2,3-dihydro-1*H*-benzo[*d*]imidazol-1-yl)pentanoate (4caa)

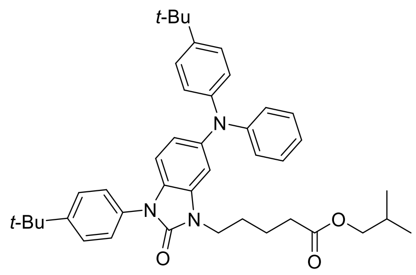

Brownish oil liquid, (100 mg, 62% yield); ^{1}H NMR (400 MHz, $CDCl_3$) δ 7.48 (dd, *J* = 25.4, 8.6 Hz, 2H), 7.26 – 7.18 (m, 4H), 7.06 (d, *J* = 8.0 Hz, 2H), 7.03 – 6.97 (m, 3H), 6.94 (t, *J* = 7.2 Hz, 1H), 6.85 (d, *J* = 1.6 Hz, 1H), 6.80 (dd, *J* = 8.4, 2.0 Hz, 1H), 3.92 – 3.75(m, 4H), 2.35 (t, *J* = 7.0 Hz, 2H), 1.94 – 1.84 (m, 1H), 1.81 – 1.65 (m, 4H), 1.36 (s, 9H), 1.31 (s, 9H), 0.90 (d, *J* = 6.8 Hz, 6H). ^{13}C NMR (100 MHz, $CDCl_3$) δ 173.32, 153.72, 150.50, 148.44, 145.42, 145.37, 142.82, 132.13, 130.38, 129.16, 126.48, 126.09, 125.98, 125.98, 125.35, 123.09, 122.67, 121.78, 119.49, 109.57, 106.01, 70.58, 40.82, 34.80, 34.35, 33.90, 31.55, 31.46, 27.88, 27.79, 22.31, 19.20. IR (KBr): 2961, 2871, 1717, 1597, 1515, 1492, 1399, 1365, 1271, 1115, 834, 788, 752, 698 cm^{-1}. MS (EI, m/z): 645 $[M]^+$. HRMS (ESI): Calcd. for $C_{42}H_{52}N_3O_3$ $[M+H]^+$: 646.4003; found: 646.4010.

(4)isobutyl-5-(3-([1,1'-biphenyl]-4-yl)-6-([1,1'-biphenyl]-4-yl(phenyl)amino)-2-oxo-2,3-dihydro-1*H*-benzo[*d*]imidazol-1-yl)pentanoate (4daa)

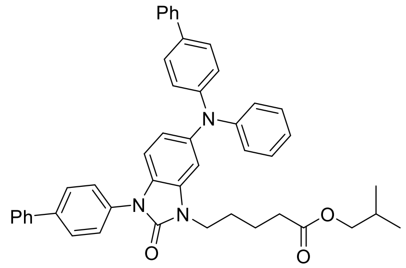

Brownish oil liquid, (113 mg, 66% yield); ^{1}H NMR (400 MHz, $CDCl_3$): δ 7.77 (d, *J* = 8.4 Hz, 2H), 7.69 – 7.64 (m, 4H), 7.61 (d, *J* = 7.2 Hz, 2H), 7.55 – 7.49 (m, 4H), 7.47 (d, *J* = 3.2 Hz, 1H), 7.44 (s, 1H), 7.43 – 7.41 (m, 1H), 7.39 (d, *J* = 7.2 Hz, 1H), 7.32 (dd, *J* = 15.0, 7.4Hz, 3H), 7.19 (d, *J* = 8.4 Hz, 3H), 7.11 (d, *J* = 8.4 Hz, 1H), 7.05 (t, *J* = 7.2 Hz, 1H), 6.96 (d, *J* = 1.6 Hz, 1H), 6.91 (dd, *J* = 8.4, 2.0 Hz, 1H), 3.92 (t, *J* = 6.6 Hz, 2H), 3.86 (d, *J* = 6.8 Hz, 2H), 2.40 (t, *J* = 7.2 Hz, 2H), 1.97 – 1.89 (m, 1H), 1.88 – 1.80 (m, 2H), 1.79 – 1.71 (m, 2H), 0.93 (d, *J* = 6.7 Hz, 6H). ^{13}C NMR (100 MHz, $CDCl_3$): δ 173.27, 153.58, 147.98, 147.49, 142.71, 140.66, 140.46, 140.37, 134.78, 133.97, 130.54, 129.35, 128.95, 128.83, 128.24, 127.82, 127.65, 127.22, 126.87, 126.66, 126.00, 125.92, 123.61, 123.03, 122.63, 119.67, 109.67, 106.16, 70.56, 40.89, 33.85, 27.85, 27.76, 22.30, 19.17. IR (KBr): 3059, 3032, 2959, 2872, 2827, 1715, 1599, 1489, 1399, 1277, 1175, 763, 698 cm^{-1}. MS (EI, m/z): 685 $[M]^+$. HRMS (ESI): Calcd. for $C_{46}H_{44}N_3O_3$ $[M+H]^+$: 686.3377; found: 686.3376.

(5)isobutyl-5-(3-(4-chlorophenyl)-6-((4-chlorophenyl)(phenyl)amino)-2-oxo-2,3-dihydro-1*H*-benzo[*d*]imidazol-1-yl)pentanoate (4eaa)

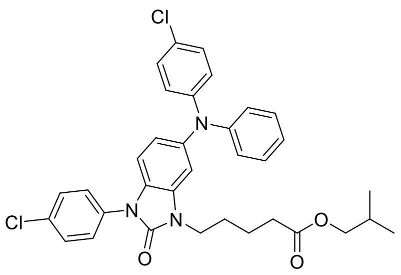

Brownish oil liquid, (96 mg, 64% yield); ^{1}H NMR (400 MHz, $CDCl_3$): δ 7.51 – 7.45 (m, 4H), 7.25 – 7.22 (m, 2H), 7.19 – 7.15 (m, 2H), 7.09 – 7.01 (m, 3H), 7.01 – 6.95 (m, 4H), 6.82 (d, *J* = 2.0 Hz, 1H), 6.79 (dd, *J* = 8.4, 2.0 Hz, 1H), 3.87 – 3.79 (m, 4H), 2.34 (t, *J* = 7.2 Hz, 2H), 1.93 – 1.83 (m, 1H), 1.79 – 1.72 (m, 2H), 1.72 – 1.64 (m, 2H), 0.89 (d, *J* = 6.4 Hz, 6H). ^{13}C NMR (100 MHz, $CDCl_3$): δ 173.27, 153.37, 147.75, 146.81, 142.75, 133.36, 133.37, 130.59, 129.78, 129.45, 129.32, 127.08, 127.02, 125.67, 124.12, 123.57, 122.94, 119.50, 109.51, 106.05, 70.62, 40.96, 33.83, 27.81, 27.79, 22.30, 19.19. IR (KBr): 2961, 2873, 1718, 1589, 1490, 1399, 1173, 1091, 824, 755, 697 cm^{-1}. MS (EI, m/z): 601 $[M]^+$. HRMS (ESI): Calcd. for $C_{34}H_{34}Cl_2N_3O_3$ $[M+H]^+$: 602.1972; found: 602.1971.

(6)isobutyl-5-(3-(4-bromophenyl)-6-((4-bromophenyl)(phenyl)amino)-2-oxo-2,3-dihydro-1*H*-benzo[*d*]imidazol-1-yl)pentanoate (4faa)

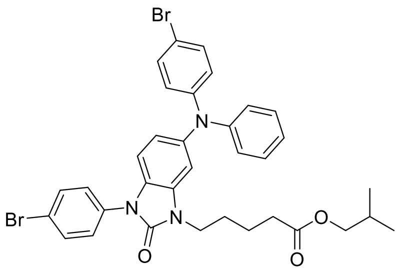

Brownish oil liquid, (105 mg, 61% yield); ^{1}H NMR (400 MHz, $CDCl_3$): δ 7.64 (d, *J* = 8.4 Hz, 2H), 7.44 (d, *J* = 8.4 Hz, 2H), 7.31 (d, *J* = 8.8 Hz, 2H), 7.24 (d, *J* = 8.8 Hz, 2H), 7.06 (d, *J* = 8.0 Hz, 2H), 7.03 (d, *J* = 7.2 Hz, 1H), 7.00 – 6.91 (m, 4H), 6.82 (s, 1H), 6.80 (d, *J* = 8.4 Hz, 1H), 3.89 – 3.79 (m, 4H), 2.35 (t, *J* = 7.0 Hz, 2H), 1.95 – 1.84 (m, 1H), 1.79 – 1.65 (m, 4H), 0.90 (d, *J* = 6.4 Hz, 6H). ^{13}C NMR (100 MHz, $CDCl_3$): δ 173.18, 153.22, 147.55, 147.23, 142.58, 133.80, 132.67, 132.15, 130.53, 129.39, 127.22, 125.55, 124.25, 123.65, 122.99, 120.99, 119.48, 114.38, 109.45, 106.02, 70.54, 40.89, 33.7, 27.72, 27.71, 22.21, 19.11. IR (KBr): 2959, 2872, 2829, 1718, 1596, 1489,1399, 1173, 1072, 1009, 820, 754, 697 cm^{-1}. MS (EI, m/z): 689 $[M]^+$. HRMS (ESI): Calcd. for $C_{34}H_{34}Br_2N_3O_3$ $[M+H]^+$: 690.0961; found: 690.0962.

(7)isobutyl-5-(3-(4-fluorophenyl)-6-((4-fluorophenyl)(phenyl)amino)-2-oxo-2,3-dihydro-1*H*-benzo[*d*]imidazol-1-yl)pentanoate (4gaa)

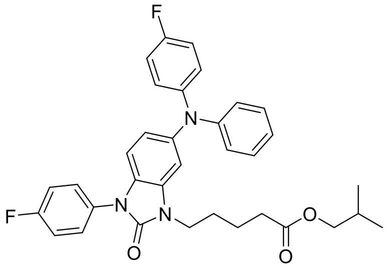

Brownish oil liquid, (95 mg, 67% yield); ^{1}H NMR (400 MHz, $CDCl_3$): δ 7.54 – 7.49 (m, 2H), 7.26 – 7.16 (m, 4H), 7.09 – 7.05 (m, 2H), 7.03 (d, *J* = 7.6 Hz, 2H), 6.99 – 6.91 (m, 4H), 6.83 (d, *J* = 1.6 Hz, 1H), 6.78 (dd, *J* = 8.4, 2.0 Hz, 1H), 3.92 – 3.18 (m, 4H), 2.36 (t, *J* = 7.2 Hz, 2H), 1.95 – 1.85 (m, 1H), 1.82 – 1.74 (m, 2H), 1.74 – 1.63 (m, 2H), 0.91 (d, *J* = 7.2 Hz, 6H). ^{13}C NMR (100 MHz, $CDCl_3$): δ 173.26, 161.61 (d, *J* = 248.5 Hz), 158.73 (d, *J* = 243.7 Hz), 153.57, 148.30, 144.20 (d, *J* = 2.7 Hz), 143.10, 130.73 (d, *J* = 3.1 Hz), 130.44, 129.30, 127.73 (d, *J* = 8.6 Hz), 125.73, 125.66 (d, *J* = 8.1 Hz), 122.48, 122.10, 119.01, 116.52 (d, *J* = 23.0 Hz), 116.12 (d, *J* = 22.5 Hz), 109.30, 105.59, 70.58, 40.89, 33.82, 27.82, 27.77, 22.29, 19.16. ^{19}F NMR (376 MHz, $CDCl_3$) δ -113.62, -119.98. IR (KBr): 3065, 2901, 2874, 1716, 1627, 1596, 1494, 1401, 1222, 833, 750, 696 cm^{-1}. MS (EI, m/z): 569 $[M]^+$. HRMS (ESI): Calcd. for $C_{34}H_{34}F_2N_3O_3$ $[M+H]^+$:570.2563; found: 570.2568.

(8)isobutyl-5-(3-(4-methoxyphenyl)-6-((4-methoxyphenyl)(phenyl)amino)-2-oxo-2,3-dihydro-1*H*-benzo[*d*]imidazol-1-yl)pentanoate (4haa)

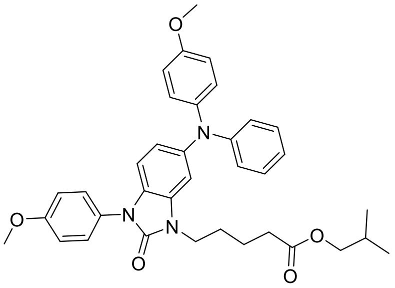

Brownish oil liquid, (93 mg, 63% yield); ^{1}H NMR (400 MHz, $CDCl_3$) δ 7.45 – 7.40 (m, 2H), 7.19 (t, *J* = 8.0 Hz, 2H), 7.10 – 7.06 (m, 2H), 7.03 (s, 1H), 7.02 – 6.97 (m, 3H), 6.92 – 6.86 (m, 2H), 6.85 (s, 1H), 6.84 – 6.80 (m, 2H), 6.77 (dd, *J* = 8.4, 2.0 Hz, 1H), 3.88 – 3.81 (m, 7H), 3.80 (s, 3H), 2.35 (t, *J* = 7.2 Hz, 2H), 1.95 – 1.84 (m, 1H), 1.80 – 1.73 (m, 2H), 1.72 – 1.65 (m, 2H), 0.90 (d, *J* = 6.4 Hz, 6H). ^{13}C NMR (100 MHz, $CDCl_3$) δ 173.37, 158.92, 155.98, 153.88, 148.83, 143.13, 141.22, 130.29, 129.14, 127.53, 127.44, 126.61, 126.01, 121.47, 121.12, 118.68, 114.86, 114.84, 109.25, 105.23, 70.61, 55.67, 55.61, 40.84, 33.92, 27.91, 27.81, 22.35, 19.21. IR (KBr): 2958, 2835, 1712, 1596, 1513, 1492, 1245, 1175, 1033, 830, 790, 748, 696 cm^{-1}. MS (EI, m/z): 593 $[M]^+$. HRMS (ESI): Calcd. for $C_{36}H_{40}N_3O_5$ $[M+H]^+$: 594.2962; found: 594.2964.

(9)isobutyl-3-(6-(diphenylamino)-2-oxo-3-phenyl-2,3-dihydro-1*H*-benzo[*d*]imidazol-1-yl)propanoate (4aba)

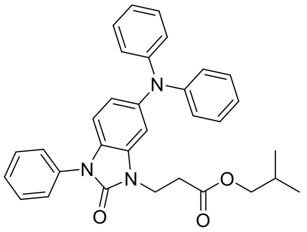

Brownish oil liquid, (57 mg, 45% yield); ^{1}H NMR (400 MHz, $CDCl_3$): δ 7.55 – 7.49 (m, 4H), 7.40 –7.36 (m, 1H), 7.26 – 7.21 (m, 4H), 7.07 (d, *J* = 7.6 Hz, 4H), 7.00 – 6.96 (m, 3H), 6.93 (d, *J* = 1.6 Hz, 1H), 6.82 (dd, *J* = 8.4, 2.0 Hz, 1H), 4.14 (t, *J* = 7.2 Hz, 2H), 3.80 (d, *J* = 6.4 Hz, 2H), 2.79 (t, *J* = 7.2 Hz, 2H), 1.94 – 1.79 (m, 1H), 0.87 (d, *J* = 6.8 Hz, 6H). ^{13}C NMR (100 MHz, $CDCl_3$): δ 171.18, 153.39, 148.23, 142.99, 134.73, 130.21, 129.63, 129.31, 127.69, 126.00,

125.91, 123.28, 122.36, 119.89, 109.64, 106.42, 71.06, 37.41, 33.08, 27.70, 19.15. IR (KBr): 2959, 2927, 2873, 2850, 1714, 1638, 1619, 1597, 1490, 1399, 1105, 754, 695, 616 cm^{-1}. MS (EI, m/z): 505 $[M]^+$. HRMS (ESI): Calcd. for $C_{32}H_{32}N_3O_3$ $[M+H]^+$: 506.2438; found: 506.2443.

(10)isobutyl-4-(6-(diphenylamino)-2-oxo-3-phenyl-2,3-dihydro-1*H*-benzo[*d*]imidazol-1-yl) butanoate (4aca)

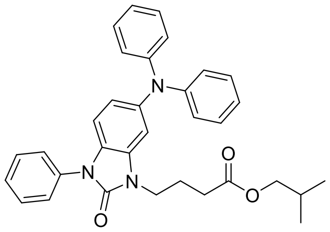

Brownish oil liquid, (87 mg, 66% yield); ^1H NMR (400 MHz, $CDCl_3$): δ 7.60 – 7.48 (m, 4H), 7.41 – 7.35 (m, 1H), 7.27 – 7.21 (m, 4H), 7.08 (d, *J* = 7.6 Hz, 4H), 7.02 – 6.95 (m, 3H), 6.90 (d, *J* = 1.6 Hz, 1H), 6.82 (dd, *J* = 8.4, 2.0 Hz, 1H), 3.91 (t, *J* = 6.8 Hz, 2H), 3.81 (d, *J* = 6.4 Hz, 2H), 2.42 (t, *J* = 7.4 Hz, 2H), 2.10 – 2.02 (m, 2H), 1.93 – 1.83 (m, 1H), 0.90 (d, *J* = 6.4 Hz, 6H). ^{13}C NMR (100 MHz, $CDCl_3$): δ 172.74, 153.51, 148.12, 142.90, 134.74, 130.36, 129.49, 129.19, 127.49, 125.78, 125.76, 123.22, 122.26, 119.54, 109.49, 105.95, 70.70, 40.48, 31.46, 27.67, 23.73, 19.11. IR (KBr): 3062, 2960, 2873, 1716, 1595, 1491, 1399, 1273, 1218, 1175, 754, 695, 656 cm^{-1}. MS (EI, m/z): 519 $[M]^+$. HRMS (ESI): Calcd. for $C_{33}H_{34}N_3O_3$ $[M+H]^+$: 520.2595; found: 520.2598.

(11)isobutyl-6-(6-(diphenylamino)-2-oxo-3-phenyl-2,3-dihydro-1*H*-benzo[*d*]imidazol-1-yl) hexanoate (4ada)

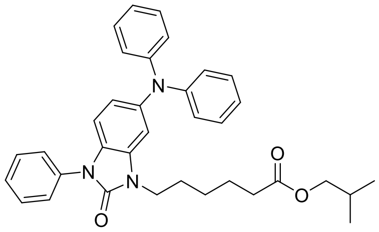

Brownish oil liquid, (85 mg, 62% yield); ^1H NMR (400 MHz, $CDCl_3$): δ 7.57 – 7.49 (m, 4H), 7.40 – 7.35 (m, 1H), 7.23 (d, *J* = 7.6 Hz, 3H), 7.08 (d, *J* = 8.0 Hz, 4H), 6.98 (t, *J* = 7.6 Hz, 3H), 6.85 (d, *J* = 2.0 Hz, 1H), 6.81 (dd, *J* = 8.4, 2.0 Hz, 1H), 3.89 – 3.79 (m, 4H), 2.29 (t, *J* = 7.6 Hz, 2H), 1.95 – 1.85 (m, 1H), 1.79 – 1.69 (m, 2H), 1.67 – 1.61 (m, 2H), 1.48 – 1.32 (m, 2H), 0.91 (d, *J* = 6.8 Hz, 6H). ^{13}C NMR (100 MHz, $CDCl_3$): δ 173.66, 153.57, 148.22, 142.86, 134.89, 130.52, 129.55, 129.27, 127.52, 125.87, 125.84, 123.26, 122.32, 119.48, 109.51, 106.10, 70.54, 41.15, 34.21, 28.14, 27.80, 26.48, 24.75, 19.20. IR (KBr): 3063, 2963, 2874, 1718, 1628, 1597, 1491, 1401, 1275, 1216, 1174, 754, 695, 657 cm^{-1}. MS (EI, m/z): 547 $[M]^+$. HRMS (ESI): Calcd. for $C_{35}H_{38}N_3O_3$ $[M+H]^+$: 548.2908; found: 548.2909.

(12) isobutyl 2-chloro-4-(6-(diphenylamino)-2-oxo-3-phenyl-2,3-dihydro-1*H*-benzo[*d*]imidazol-1-yl)-2-methylbutanoate (4aea)

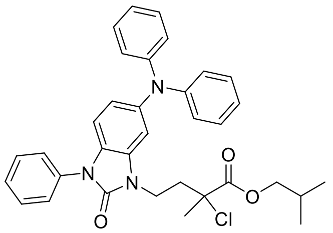

Brownish oil liquid, (80 mg, 56% yield);^{1}H NMR (400 MHz, $CDCl_3$): δ 7.55 – 7.48 (m, 4H), 7.39 – 7.34 (m, 1H), 7.23 (t, *J* = 7.8 Hz, 4H), 7.09 (d, *J* = 7.6 Hz, 4H), 7.00 – 6.95 (m, 3H), 6.92 (d, *J* = 2.0 Hz, 1H), 6.83 (dd, *J* = 8.4, 2.0 Hz, 1H), 4.11 – 3.96 (m, 2H), 3.91 – 3.81 (m, 2H), 2.55 – 2.46 (m, 1H), 2.42 – 2.34 (m, 1H), 1.98 –1.87 (m, 1H), 1.81 (s, 3H), 0.91 (d, *J* = 6.4 Hz, 6H). ^{13}C NMR (100 MHz, $CDCl_3$): δ 170.51, 153.25, 148.13, 143.10, 134.77, 130.02, 129.55, 129.27, 127.56, 125.76, 125.68, 123.46, 122.45, 119.26, 109.53, 105.81, 72.29, 66.86, 39.35, 37.59, 28.09, 27.75, 19.05. IR (KBr): 3063, 2963, 2874, 1718, 1628,1597, 1491, 1401, 1275, 1217, 1174, 754, 695, 619 cm^{-1}. MS (EI, m/z): 567 $[M]^+$. HRMS (ESI): Calcd. for $C_{34}H_{34}ClN_3O_3$ $[M+H]^+$: 568.2361; found: 568.2364.

(13)butyl-5-(6-(diphenylamino)-2-oxo-3-phenyl-2,3-dihydro-1*H*-benzo[*d*]imidazol-1-yl)pentanoate (4aab)

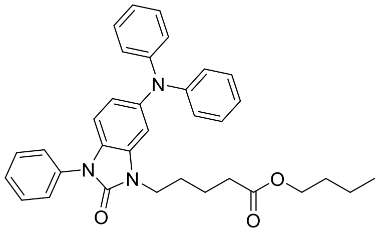

Brownish oil liquid, (96 mg, 72% yield);^{1}H NMR (400 MHz, $CDCl_3$): δ 7.57 –7.48 (m, 4H), 7.37 (t, *J* = 7.2 Hz, 1H), 7.24 (t, *J* = 7.8 Hz, 4H), 7.09 (d, *J* = 8.0 Hz, 4H), 6.98 (t, *J* = 7.6 Hz, 3H), 6.86 (d, *J* = 1.6 Hz, 1H), 6.81 (dd, *J* = 8.4, 2.0 Hz, 1H), 4.04 (t, *J* = 6.6 Hz, 2H), 3.86 (t, *J* = 6.6 Hz, 2H), 2.34 (t, *J* = 7.2 Hz, 2H), 1.79 – 1.66 (m, 4H), 1.62 – 1.55 (m, 2H), 1.40 – 1.31 (m, 2H), 0.92 (t, *J* = 7.4 Hz, 3H). ^{13}C NMR (100 MHz, $CDCl_3$): δ 173.35, 153.59, 148.22, 142.90, 134.88, 130.49, 129.55, 129.2, 127.54, 125.90, 125.86, 123.29, 122.33, 119.52, 109.54, 106.05, 64.36, 40.87, 33.90, 30.76, 27.84, 22.30, 19.23, 13.81. IR (KBr): 3062, 2958, 2932, 2870, 1716, 1626, 1595, 1491, 1399, 1274, 1174, 754, 722, 695 cm^{-1}. MS (EI, m/z): 533 $[M]^+$. HRMS (ESI): Calcd. for $C_{34}H_{36}N_3O_3$ $[M+H]^+$: 534.2751; found: 534.2754.

(46)octyl-5-(6-(diphenylamino)-2-oxo-3-phenyl-2,3-dihydro-1*H*-benzo[*d*]imidazol-1-yl)pentanoate (4aac)

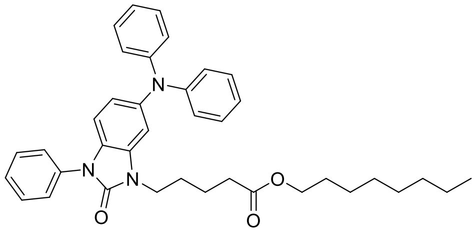

Brownish oil liquid, (105 mg, 71% yield); ^{1}H NMR (400 MHz, $CDCl_3$): δ 7.58 – 7.47 (m, 4H), 7.36 (t, *J* = 7.2 Hz, 1H), 7.23 (t, *J* = 7.8 Hz, 4H), 7.08 (d, *J* = 8.0 Hz, 4H), 7.00 – 6.95 (m, 3H),

6.86 (d, J = 1.2 Hz, 1H), 6.80 (dd, J = 8.4, 2.0 Hz, 1H), 4.03 (t, J = 6.8 Hz, 2H), 3.85 (t, J = 6.8 Hz, 2H), 2.33 (t, J = 7.0 Hz, 2H), 1.81 – 1.73 (m, 2H), 1.72 – 1.64 (m, 2H), 1.61 – 1.55 (m, 2H), 1.26 (s, 10H), 0.87 (t, J = 6.4 Hz, 3H). ^{13}C NMR (100 MHz, $CDCl_3$): δ 173.26, 153.50, 148.14, 142.82, 134.81, 130.41, 129.47, 129.20, 127.46, 125.81, 125.78, 123.21, 122.26, 119.44, 109.47, 105.98, 64.59, 40.79, 33.81, 31.81, 29.23, 29.19, 28.65, 27.76, 25.94, 22.66, 22.21, 14.13. IR (KBr): 3063, 2928, 2856, 1718, 1595, 1491, 1399, 1275, 1173, 753, 695 cm^{-1}. MS (EI, m/z): 589 $[M]^+$. HRMS (ESI): Calcd. for $C_{38}H_{44}N_3O_3$ $[M+H]^+$: 590.3377; found: 590.3375.

(15)neopentyl-5-(6-(diphenylamino)-2-oxo-3-phenyl-2,3-dihydro-1*H*-benzo[*d*]imidazol-1-yl)pentanoate (4aad)

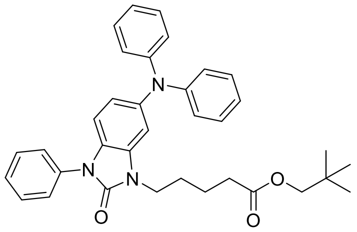

Brownish oil liquid, (98 mg, 72% yield); ^{1}H NMR (400 MHz, $CDCl_3$): δ 7.57 – 7.49 (m, 4H), 7.37 (t, J = 7.0 Hz, 1H), 7.26 – 7.21 (m, 4H), 7.09 (d, J = 8.0 Hz, 4H), 6.98 (t, J = 7.6 Hz, 3H), 6.86 (s, 1H), 6.82 (d, J = 8.4 Hz, 1H), 3.87 (t, J = 6.6 Hz, 2H), 3.76 (s, 2H), 2.38 (t, J = 7.0 Hz, 2H), 1.83 – 1.67 (m, 4H), 0.92 (s, 9H). ^{13}C NMR (100 MHz, $CDCl_3$): δ 173.30, 153.56, 148.19, 142.88, 134.86, 130.46, 129.53, 129.26, 127.51, 125.87, 125.83, 123.32, 122.27, 119.50, 109.52, 106.03, 73.77, 40.83, 33.89, 31.35, 27.87, 26.53, 22.32. IR (KBr): 3062, 2958, 2870, 1717, 1595, 1491, 1400, 1370, 1275, 1173, 754, 696, 658 cm^{-1}. MS (EI, m/z): 547 $[M]^+$. HRMS (ESI): Calcd. for $C_{35}H_{38}N_3O_3$ $[M+H]^+$: 548.2908; found: 548.2911.

(16)benzyl-5-(6-(diphenylamino)-2-oxo-3-phenyl-2,3-dihydro-1*H*-benzo[*d*]imidazol-1-yl)pentanoate (4aae)

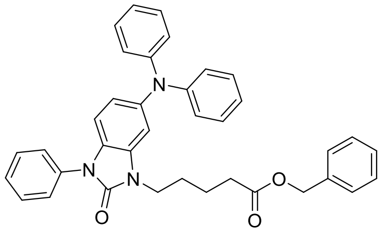

Brownish oil liquid, (88 mg, 62% yield); ^{1}H NMR (400 MHz, $CDCl_3$): δ 7.57 – 7.52 (m, 3H), 7.51 (d, J = 8.4 Hz, 1H), 7.41 – 7.29 (m, 7H), 7.23 (d, J = 7.6 Hz, 3H), 7.09 (d, J = 7.6 Hz, 4H), 6.98 (t, J = 8.0 Hz, 3H), 6.86 (d, J = 2.0 Hz, 1H), 6.82 (dd, J = 8.4, 2.0 Hz, 1H), 5.09 (s, 2H), 3.86 (t, J = 6.6 Hz, 2H), 2.40 (t, J = 7.0 Hz, 2H), 1.82 – 1.74 (m, 2H), 1.74 – 1.67 (m, 2H). ^{13}C NMR (100 MHz, $CDCl_3$): δ 173.08, 148.22, 142.91, 136.08, 134.86, 130.47, 129.57, 129.30, 128.67, 128.31, 128.29, 127.57, 125.88, 123.30, 122.35, 119.54, 109.57, 106.05, 100.09, 66.32, 40.84, 33.85, 27.81, 22.24. IR (KBr): 3034, 2990, 2936, 2828, 1713, 1630, 1595, 1491, 1399, 753, 695 cm^{-1}. MS (EI, m/z): 567 $[M]^+$. HRMS (ESI): Calcd. for $C_{37}H_{34}N_3O_3$ $[M+H]^+$: 568.2595; found: 568.2592.

(17)thiophen-2-ylmethyl-5-(6-(diphenylamino)-2-oxo-3-phenyl-2,3-dihydro-1*H*-benzo[*d*]i

midazol-1-yl)pentanoate (4aaf)

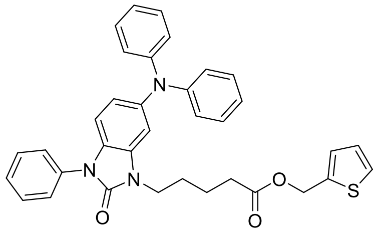

Brownish oil liquid, (102 mg, 71% yield); ^{1}H NMR (400 MHz, $CDCl_3$): δ 7.56 – 7.49 (m, 4H), 7.40 – 7.35 (m, 1H), 7.27 (dd, *J* = 5.2, 0.8 Hz, 1H), 7.25 – 7.21 (m, 4H), 7.09 (d, *J* = 8.0 Hz, 4H), 7.06 (d, *J* = 3.2 Hz, 1H), 7.00 (s, 1H), 6.99 – 6.94 (m, 3H), 6.85 (d, *J* = 2.0 Hz, 1H), 6.82 (dd, *J* = 8.4, 2.0 Hz, 1H), 5.23 (s, 2H), 3.85 (t, *J* = 6.8 Hz, 2H), 2.37 (t, *J* = 7.0 Hz, 2H), 1.79 – 1.73 (m, 2H), 1.73 – 1.64 (m, 2H). ^{13}C NMR (100 MHz, $CDCl_3$): δ 172.86, 153.57, 148.21, 142.89, 138.10, 134.86, 130.46, 129.55, 129.28, 128.17, 127.54, 126.88, 125.88, 125.86, 123.29, 122.34, 119.52, 109.54, 106.05, 60.51, 40.81, 33.74, 27.74, 22.17. IR (KBr): 3063, 2948, 2831, 1713, 1594, 1491, 1370, 754, 696 cm^{-1}. MS (EI, m/z): 573 $[M]^+$. HRMS (ESI): Calcd. for $C_{35}H_{32}N_3O_3S$ $[M+H]^+$: 574.2159; found: 574.2158.

(18)2-methoxyethyl-5-(6-(diphenylamino)-2-oxo-3-phenyl-2,3-dihydro-1*H*-benzo[*d*]imidazol-1-yl)pentanoate (4aag)

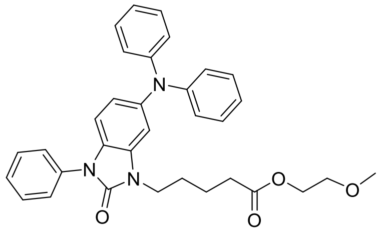

Brownish oil liquid, (84 mg, 63% yield); ^{1}H NMR (400 MHz, $CDCl_3$): δ 7.56 – 7.48 (m, 4H), 7.36 (t, *J* = 7.0 Hz, 1H), 7.23 (t, *J* = 7.8 Hz, 4H), 7.08 (d, *J* = 8.0 Hz, 4H), 6.97 (t, *J* = 7.6 Hz, 3H), 6.85 (d, *J* = 1.6 Hz, 1H), 6.81 (dd, *J* = 8.4, 2.0 Hz, 1H), 4.19 (t, *J* = 4.8 Hz, 2H), 3.85 (t, *J* = 6.8 Hz, 2H), 3.55 (t, J = 4.8 Hz, 2H), 3.35 (s, 3H), 2.38 (t, *J* = 7.2 Hz, 2H), 1.80 – 1.65 (m, 4H). ^{13}C NMR (100 MHz, $CDCl_3$): δ 173.18, 153.54, 148.17, 142.86, 134.82, 130.43, 129.51, 129.24, 127.51, 125.84, 125.83, 123.24, 122.29, 119.49, 109.51, 106.02, 70.50, 63.44, 59.02, 40.83, 33.68, 27.75, 22.18. IR (KBr): 3061, 2944, 1714, 1595, 1491, 1400, 1275, 1174, 754, 696, 658 cm^{-1}. MS (EI, m/z): 535 $[M]^+$. HRMS (ESI): Calcd. for $C_{33}H_{34}N_3O_4$ $[M+H]^+$: 536.2544; found: 536.2550.

(19)5-(diphenylamino)-3-ethyl-1-phenyl-1,3-dihydro-2*H*-benzo[*d*]imidazol-2-one (5af)

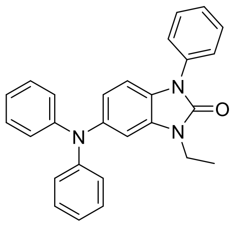

Brownish solid, (78 mg, 78% yield), m.p: 162-163 °C; ^{1}H NMR (400 MHz, $CDCl_3$) δ 7.58 – 7.48 (m, 4H), 7.37 (t, *J* = 7.2 Hz, 1H), 7.20 – 7.26 (m, 4H), 7.08 (d, *J* = 7.6 Hz, 4H), 6.97 (t, *J* = 7.6

Hz, 3H), 6.87 (s, 1H), 6.81 (d, *J* = 8.4 Hz, 1H), 3.90 (q, *J* = 7.2 Hz, 2H), 1.30 (t, *J* = 7.2 Hz, 3H). ^{13}C NMR (100 MHz, $CDCl_3$) δ 153.34, 148.28, 142.88, 134.94, 130.24, 129.57, 129.29, 127.55, 126.01, 125.91, 123.29, 122.32, 119.51, 109.53, 106.03, 36.14, 13.64. IR (KBr): 3058, 3033, 2974, 2929, 2855, 1712, 1592, 1491, 1400, 1276, 1234, 1192, 1082, 1022, 754, 695, 656 cm^{-1}. MS (EI, m/z): 405 $[M]^+$. HRMS (ESI): Calcd. for $C_{27}H_{23}N_3NaO$ $[M+Na]^+$: 428.1733; found: 428.1727.

(20)5-(diphenylamino)-1-phenyl-3-propyl-1,3-dihydro-2*H*-benzo[*d*]imidazol-2-one (3ag)

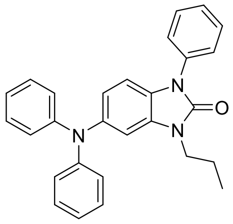

Brownish oil liquid, (90 mg, 86% yield); ^{1}H NMR (400 MHz, $CDCl_3$): δ 7.42 – 7.60 (m, 4H), 7.38 – 7.31 (m, 1H), 7.19 – 7.24 (m, 4H), 7.11 – 7.04 (m, 4H), 7.01 – 6.92 (m, 3H), 6.87 (d, *J* = 2.0 Hz, 1H), 6.80 (dd, *J* = 8.4, 2.0 Hz, 1H), 3.79 (t, *J* = 7.2 Hz, 2H), 1.79 – 1.70 (m, 2H), 0.94 (t, *J* = 7.4 Hz, 3H). ^{13}C NMR (100 MHz, $CDCl_3$): δ 153.59, 148.20, 142.74, 134.91, 130.68, 129.48, 129.22, 127.44, 125.88, 125.81, 123.18, 122.24, 119.46, 109.42, 106.20, 42.86, 21.71, 11.43. IR (KBr): 3030, 2965, 2923, 2875, 1714, 1608, 1593, 1518, 1492, 1400, 1271, 1224, 1075, 753, 695, 658 cm^{-1}. MS (EI, m/z): 419 $[M]^+$. HRMS (ESI): Calcd. for $C_{28}H_{25}N_3NaO$ $[M+Na]^+$: 442.1890; found: 442.1887.

(21)3-butyl-5-(diphenylamino)-1-phenyl-1,3-dihydro-2*H*-benzo[*d*]imidazol-2-one (5ah)

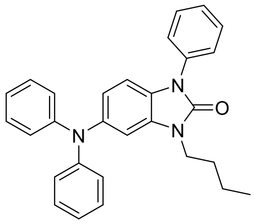

Brownish oil liquid, (80 mg, 74% yield); ^{1}H NMR (400 MHz, $CDCl_3$): δ 7.57 – 7.47 (m, 4H), 7.36 (t, *J* = 7.2 Hz, 1H), 7.23 (t, *J* = 7.4 Hz, 4H), 7.08 (d, *J* = 8.0 Hz, 4H), 6.97 (t, *J* = 7.8 Hz, 3H), 6.86 (s, 1H), 6.80 (d, *J* = 8.8 Hz, 1H), 3.83 (t, *J* = 7.0 Hz, 2H), 1.73 – 1.65 (m, 2H), 1.41 – 1.32 (m, 2H), 0.91 (t, *J* = 7.4 Hz, 3H). ^{13}C NMR (100 MHz, $CDCl_3$): δ 153.61, 148.23, 142.80, 134.94, 130.65, 129.53, 129.25, 127.48, 125.85, 123.25, 122.29, 119.39, 109.45, 106.19, 41.08, 30.50, 20.15, 13.83. IR (KBr): 3060, 3036, 2956, 2930, 2866, 1715, 1593, 1490, 1399, 1373, 1274, 1217, 1181, 1026, 754, 695, 658 cm^{-1}. MS (EI, m/z): 433 $[M]^+$. HRMS (ESI): Calcd. for $C_{29}H_{27}N_3NaO$ $[M+Na]^+$: 456.2046; found: 4562043.

(22)5-(diphenylamino)-3-isobutyl-1-phenyl-1,3-dihydro-2*H*-benzo[*d*]imidazol-2-one (5ai)

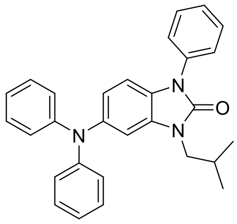

Brownish oil liquid, (67 mg, 62% yield); ^{1}H NMR (400 MHz, $CDCl_3$): δ 7.57 – 7.48 (m, 4H), 7.39 – 7.34 (m, 1H), 7.20 – 7.25 (m, 4H), 7.05 – 7.10 (m, 4H), 7.00 – 6.95 (m, 3H), 6.85 (d, *J* = 2.0 Hz, 1H), 6.80 (dd, *J* = 8.4, 2.0 Hz, 1H), 3.64 (d, *J* = 7.2 Hz, 2H), 2.10 – 2.21 (m, 1H), 0.94 (d, *J* = 6.4 Hz, 6H). ^{13}C NMR (100 MHz, $CDCl_3$): δ 153.92, 148.25, 142.76, 134.99, 131.10, 129.55, 129.27, 127.51, 125.89, 123.24, 122.30, 119.49, 109.44, 106.56, 48.78, 28.09, 20.33. IR (KBr): 3057, 2959, 2924, 2852, 1715, 1631, 1595, 1490, 1398, 1274, 1224, 1188, 1088, 1026, 753, 695, 656 cm^{-1}. MS (EI, m/z): 433 $[M]^+$. HRMS (ESI): Calcd. for $C_{29}H_{27}N_3NaO$ $[M+Na]^+$: 456.2046; found: 456.2045.

(23)5-(diphenylamino)-3-hexyl-1-phenyl-1,3-dihydro-2*H*-benzo[*d*]imidazol-2-one (5aj)

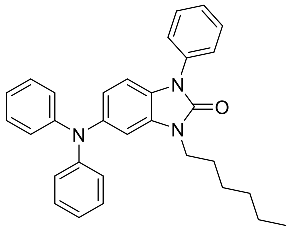

Brownish oil liquid, (86 mg, 75% yield); ^{1}H NMR (400 MHz, $CDCl_3$): δ 7.46 – 7.60 (m, 4H), 7.32 – 7.39 (m, 1H), 7.23 (t, *J* = 7.6 Hz, 4H), 7.08 (d, *J* = 7.6 Hz, 4H), 7.01 – 6.94 (m, 3H), 6.86 (d, *J* = 2.0 Hz, 1H), 6.80 (dd, *J* = 8.4, 2.0 Hz, 1H), 3.82 (t, *J* = 7.2 Hz, 2H), 1.741.66 (m, 2H), 1.36 – 1.24 (m, 6H), 0.85 (t, *J* = 7.0 Hz, 3H). ^{13}C NMR (100 MHz, $CDCl_3$): δ 153.58, 148.24, 142.80, 134.96, 130.67, 129.52, 129.26, 127.47, 125.90, 125.85, 123.25, 122.29, 119.39, 109.45, 106.20, 41.37, 31.51, 28.31, 26.54, 22.55, 14.12. IR (KBr): 3066, 3033, 2953, 2928, 2857, 1715, 1626, 1594, 1490, 1398, 1370, 1274, 1176, 1092, 753, 695, 657 cm^{-1}. MS (EI, m/z): 461 $[M]^+$. HRMS (ESI): Calcd. for $C_{31}H_{31}N_3NaO$ $[M+Na]^+$: 484.2359; found: 484.2363.

(24)5-(diphenylamino)-3-isopropyl-1-phenyl-1,3-dihydro-2*H*-benzo[*d*]imidazol-2-one (5al)

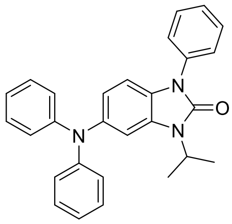

Brownish solid, (37 mg, 35% yield), m.p: 89-90 °C; ^{1}H NMR (400 MHz, $CDCl_3$): δ 7.48 – 7.55 (m, 4H), 7.37 (t, *J* = 6.6 Hz, 1H), 7.23 (d, *J* = 7.6 Hz, 3H), 7.09 (d, *J* = 7.6 Hz, 4H), 6.97 (d, *J* = 7.2 Hz, 4H), 6.79 (d, *J* = 8.0 Hz, 1H), 4.71 – 4.61 (m, 1H), 1.49 (d, *J* = 6.8 Hz, 6H). ^{13}C NMR (100 MHz, $CDCl_3$): δ 153.06, 148.28, 142.54, 134.89, 129.56, 129.43, 129.29, 127.59, 126.11,

123.29, 122.32, 119.12, 109.43, 107.38, 45.42, 20.23. IR (KBr): 3063, 3042, 2968, 2929, 2869, 1712, 1629, 1593, 1491, 1388, 1273, 1236, 1176, 752, 695, 660 cm^{-1}. MS (EI, m/z): 419 $[M]^+$. HRMS (ESI): Calcd. for $C_{28}H_{25}N_3NaO$ $[M+Na]^+$: 442.1890; found: 442.1888.

(25)3-allyl-5-(diphenylamino)-1-phenyl-1,3-dihydro-2*H*-benzo[*d*]imidazol-2-one (5am)

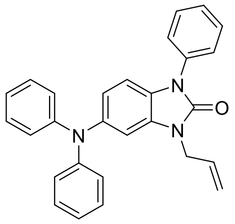

Brownish oil liquid, (65 mg, 62% yield); ^1H NMR (400 MHz, $CDCl_3$): δ 7.61 – 7.47 (m, 4H), 7.35 – 7.41 (m, 1H), 7.19 – 7.27 (m, 4H), 7.02 – 7.11(m, 4H), 7.02 – 6.94 (m, 3H), 6.85 – 6.80 (m, 2H), 5.81 – 5.93(m, 1H), 5.12 – 5.22(m, 2H), 4.52 – 4.41 (m, 2H). ^{13}C NMR (100 MHz, $CDCl_3$): δ 153.42, 148.21, 142.92, 134.93, 131.73, 130.39, 129.60, 129.28, 127.62, 125.92, 123.39, 122.39, 119.46, 118.07, 109.51, 106.61, 43.66. IR (KBr): 3066, 3024, 2959, 2929, 2852, 1716, 1635, 1599, 1489, 1395, 1273, 1220, 1177, 752, 696, 655 cm^{-1}. MS (EI, m/z): 417 $[M]^+$. HRMS (ESI): Calcd. for $C_{28}H_{23}N_3NaO$ $[M+Na]^+$: 440.1733; found: 440.1729.

(26)3-ethyl-5-(phenyl(p-tolyl)amino)-1-(p-tolyl)-1,3-dihydro-2*H*-benzo[*d*]imidazol-2-one (5bf)

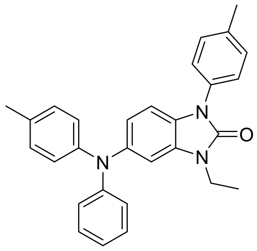

Brownish oil liquid, (89mg, 82% yield); ^1H NMR (400 MHz, $CDCl_3$): δ 7.43 – 7.39 (m, 2H), 7.30 (d, *J* = 8.4 Hz, 2H), 7.23 – 7.18 (m, 2H), 7.07 – 6.99 (m, 6H), 6.95 – 6.91 (m, 2H), 6.85 (d, *J* = 2.0 Hz, 1H), 6.78 (dd, *J* = 8.4, 2.0 Hz, 1H), 3.89 (q, *J* = 7.2 Hz, 2H), 2.40 (s, 3H), 2.31 (s, 3H), 1.30 (t, *J* = 7.2 Hz, 3H). ^{13}C NMR (100 MHz, $CDCl_3$): δ 153.45, 148.60, 145.71, 142.96, 137.46, 132.32, 132.28, 130.17, 130.15, 129.97, 129.18, 126.04, 125.83, 124.07, 122.55, 121.70, 119.14, 109.41, 105.68, 36.10, 21.28, 20.90, 13.68. IR (KBr): 3030, 2968, 2926, 2855, 1714, 1627, 1594, 1508, 1492, 1401, 1294, 1234, 811, 750, 696 cm^{-1}. MS (EI, m/z): 433 $[M]^+$. HRMS (ESI): Calcd. for $C_{29}H_{27}N_3NaO$ $[M+Na]^+$:456.2046; found: 456.2047.

(27)1-(4-(tert-butyl)phenyl)-5-((4-(tert-butyl)phenyl)(phenyl)amino)-3-ethyl-1,3-dihydro-2 *H*-benzo[*d*]imidazol-2-one (5cf)

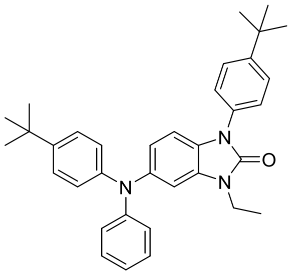

Brownish oil liquid, (65 mg, 50% yield); ^{1}H NMR (400 MHz, $CDCl_3$): δ 7.49 (dd, *J* = 24.0, 8.8 Hz, 4H), 7.25 – 7.18 (m, 4H), 7.06 (d, *J* = 7.8 Hz, 2H), 7.03 (s, 1H), 7.00 (d, *J* = 4.6 Hz, 1H), 6.97 (s, 1H), 6.94 (t, *J* = 7.2 Hz, 1H), 6.88 (d, *J* = 1.8 Hz, 1H), 6.80 (dd, *J* = 8.4, 1.8 Hz, 1H), 3.90 (q, *J* = 7.2 Hz, 2H), 1.36 (s, 9H), 1.31 (s, 9H), 1.31(s, 3H). ^{13}C NMR (100 MHz, $CDCl_3$): δ 153.46, 150.49, 148.51, 145.48, 145.35, 142.81, 132.21, 130.15, 129.17, 126.48, 126.10, 125.40, 123.08, 122.70, 121.77, 119.46, 109.55, 105.97, 36.11, 34.80, 34.36, 31.56, 31.47, 13.66. IR (KBr): 3040, 2959, 2927, 2869, 1714, 1598, 1493, 1402, 1270, 1234, 829, 731, 693 cm^{-1}. MS (EI, m/z): 517 $[M]^+$. HRMS (ESI): Calcd. for $C_{35}H_{39}N_3NaO$ $[M+Na]^+$: 540.2085; found:540.2990.

(28)1-([1,1'-biphenyl]-4-yl)-5-([1,1'-biphenyl]-4-yl(phenyl)amino)-3-ethyl-1,3-dihydro-2*H*-benzo[*d*]imidazol-2-one (5df)

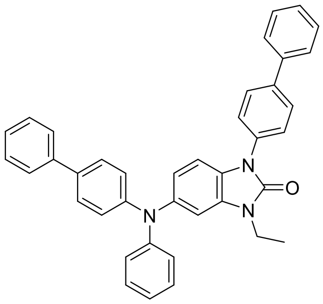

Brownish solid, (75 mg, 54% yield); m.p: 76-77 °C; ^{1}H NMR (400 MHz, $CDCl_3$): δ 7.75 – 7.70 (m, 2H), 7.65 – 7.60 (m, 4H), 7.59 – 7.56 (m, 2H), 7.49 – 7.46 (m, 3H), 7.44 – 7.41 (m, 2H), 7.40 – 7.33 (m, 2H), 7.32 – 7.25 (m, 3H), 7.17 – 7.11 (m, 4H), 7.07 (d, *J* = 8.4 Hz, 1H), 6.99 – 7.04 (m, 1H), 6.92 (d, *J* = 1.6 Hz, 1H), 6.88 (dd, *J* = 8.4, 2.0 Hz, 1H), 3.93 (q, *J* = 7.2 Hz, 2H), 1.33 (t, *J* = 7.2 Hz, 3H). ^{13}C NMR (100 MHz, $CDCl_3$): δ 153.27, 147.98, 147.50, 142.65, 140.63, 140.42, 140.36, 134.74, 133.99, 130.25, 129.29, 128.90, 128.78, 128.20, 127.76, 127.59, 127.19, 126.83, 126.62, 125.98, 123.55, 122.99, 122.55, 119.57, 109.58, 106.07, 36.11, 13.59. IR (KBr): 3060, 3033, 2957, 2928, 2870, 1713, 1590, 1519, 1485, 1398, 1276, 1189, 829, 761, 695 cm^{-1}. MS (EI, m/z): 557 $[M]^+$. HRMS (ESI): Calcd. for $C_{39}H_{31}N_3NaO$ $[M+Na]^+$: 580.2359; found: 580.2354.

(29)1-(4-chlorophenyl)-5-((4-chlorophenyl)(phenyl)amino)-3-ethyl-1,3-dihydro-2*H*-benzo[*d*]imidazol-2-one (5ef)

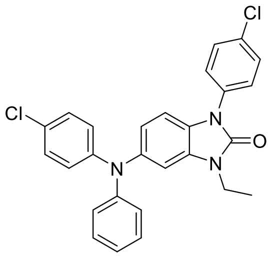

Brownish oil liquid, (65 mg, 55% yield); ^{1}H NMR (400 MHz, $CDCl_3$): δ 7.54 – 7.44 (m, 4H), 7.25 – 7.22 (m, 2H), 7.19 – 7.16 (m, 2H), 7.08 – 7.05 (m, 2H), 7.03 – 6.96 (m, 4H), 6.84 (d, *J* = 2.0 Hz, 1H), 6.80 (dd, *J* = 8.4, 2.0 Hz, 1H), 3.89 (q, *J* = 7.2 Hz, 2H), 1.30 (t, *J* = 7.2 Hz, 3H). ^{13}C NMR (100 MHz, $CDCl_3$): δ 153.10, 147.79, 146.87, 142.73, 133.43, 133.14, 130.34, 129.78, 129.45, 129.32, 127.09, 127.04, 125.77, 124.15, 123.56, 122.92, 119.44, 109.49, 105.99, 36.21, 13.60. IR (KBr): 3060, 2974, 2930, 2873, 1714, 1627, 1590, 1490, 1400, 1308, 1278, 1235, 1192, 1091, 1013, 820, 740, 696 cm^{-1}. MS (EI, m/z): 473 $[M]^+$. HRMS (ESI): Calcd. for $C_{27}H_{21}Cl_2N_3NaO$ $[M+Na]^+$: 496.0954; found: 496.0947.

(30)1-(4-bromophenyl)-5-((4-bromophenyl)(phenyl)amino)-3-ethyl-1,3-dihydro-2*H*-benzo[*d*]imidazol-2-one (5ff)

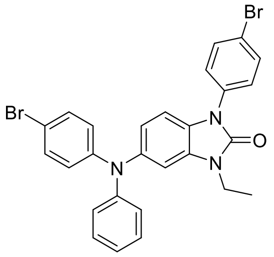

Brownish oil liquid, (105 mg, 75% yield); ^{1}H NMR (400 MHz, $CDCl_3$): δ 7.64 (d, *J* = 7.2 Hz, 2H), 7.44 (d, *J* = 7.6 Hz, 2H), 7.31 (d, *J* = 7.2 Hz, 2H), 7.24 (d, *J* = 7.6 Hz, 2H), 7.07 (d, *J* = 7.6 Hz, 2H), 7.04 – 6.96 (m, 2H), 6.93 (d, *J* = 7.6 Hz, 2H), 6.84 (s, 1H), 6.80 (d, *J* = 8.4 Hz, 1H), 3.89 (q, *J* = 6.8 Hz, 2H), 1.30 (t, *J* = 6.8 Hz, 3H). ^{13}C NMR (100 MHz, $CDCl_3$): δ 153.02, 147.67, 147.37, 142.64, 133.93, 132.76, 132.23, 130.35, 129.48, 127.33, 125.72, 124.36, 123.71, 123.05, 121.05, 119.51, 114.45, 109.52, 106.05, 36.22, 13.62. IR (KBr): 3039, 2959, 2925, 2852, 1708, 1638, 1490, 1403, 1307, 1232, 1072, 810, 748, 700 cm^{-1}. MS (EI, m/z): 561 $[M]^+$. HRMS (ESI): Calcd. for $C_{27}H_{21}Br_2N_3NaO$ $[M+Na]^+$: 583.9944; found:583.9925.

(31)4-(5-((4-cyanophenyl)(phenyl)amino)-3-ethyl-2-oxo-2,3-dihydro-1*H*-benzo[*d*]imidazol-1-yl)benzonitrile (5if)

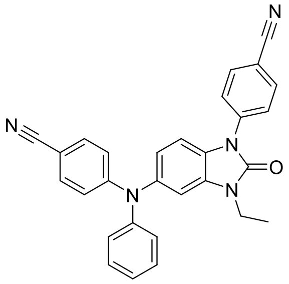

Brownish solid, (35 mg, 31% yield); m.p: 123-124 °C; ^{1}H NMR (400 MHz, $CDCl_3$): δ 7.83 (d, *J*

= 8.6 Hz, 2H), 7.75 (d, *J* = 8.6 Hz, 2H), 7.45 – 7.42 (m, 2H), 7.33 – 7.38 (m, 2H), 7.20 – 7.12 (m, 4H), 6.94 – 6.98 (m, 2H), 6.91 (dd, *J* = 7.2, 1.8 Hz, 2H), 3.93 (q, *J* = 7.2 Hz, 2H), 1.33 (t, *J* = 7.2 Hz, 3H). ^{13}C NMR (100 MHz, $CDCl_3$): δ 152.63, 151.81, 146.00, 141.61, 138.94, 133.59, 133.37, 130.76, 129.97, 125.84, 125.81, 125.67, 125.31, 122.29, 120.62, 119.71, 119.38, 118.30, 110.89, 110.02, 107.16, 102.65, 36.42, 13.52. IR (KBr): 3060, 2962, 2925, 2855, 1714, 1628, 1600, 1492, 1397, 1320, 1235, 1174, 1086, 828, 741, 700 cm^{-1}. MS (EI, m/z): 455 $[M]^+$. HRMS (ESI): Calcd. for $C_{29}H_{21}N_5NaO$ $[M+Na]^+$: 478.1638; found: 478.1636.

(32)5-(phenyl(p-tolyl)amino)-3-propyl-1-(p-tolyl)-1,3-dihydro-2*H*-benzo[*d*]imidazol-2-one (5bg)

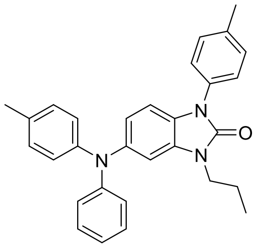

Brownish oil liquid, (80 mg, 72% yield); ^{1}H NMR (400 MHz, $CDCl_3$): δ 7.41 (d, *J* = 8.0 Hz, 2H), 7.29 (d, *J* = 8.4 Hz, 2H), 7.22 – 7.17 (m, 2H), 7.07 – 6.98 (m, 6H), 6.92 (t, *J* = 7.8 Hz, 2H), 6.85 (d, *J* = 1.8 Hz, 1H), 6.77 (dd, *J* = 8.4, 2.0 Hz, 1H), 3.79 (t, *J* = 7.2 Hz, 2H), 2.40 (s, 3H), 2.30 (s, 3H), 1.79 – 1.69 (m, 2H), 0.94 (t, *J* = 7.4 Hz, 3H).^{13}C NMR (100 MHz, $CDCl_3$): δ 153.73, 148.55, 145.66, 142.84, 137.37, 132.27, 132.23, 130.60, 130.11, 129.93, 129.14, 125.95, 125.76, 123.98, 122.44, 121.63, 119.15, 109.33, 105.88, 42.85, 21.75, 21.25, 20.87, 11.46. IR (KBr): 3057, 3034, 2965, 2925, 2874, 1714, 1593, 1512, 1490, 1399, 1314, 1223, 1187, 1019, 811, 746, 696 cm^{-1}. MS (EI, m/z): 447 $[M]^+$. HRMS (ESI): Calcd. for $C_{30}H_{29}N_3NaO$ $[M+Na]^+$: 470.2203; found: 470.2205.

(33)1-(4-ethylphenyl)-5-((4-ethylphenyl)(phenyl)amino)-3-propyl-1,3-dihydro-2*H*-benzo[*d*]imidazol-2-one (5jg)

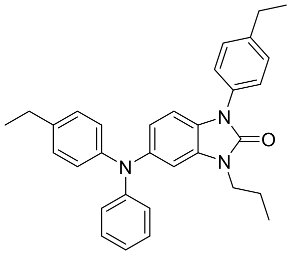

Brownish oil liquid, (97 mg, 82% yield); ^{1}H NMR (400 MHz, $CDCl_3$): δ 7.44 (d, *J* = 8.0 Hz, 2H), 7.32 (d, *J* = 8.4 Hz, 2H), 7.22 – 7.17 (m, 2H), 7.08 – 7.00 (m, 6H), 6.96 – 6.90 (m, 2H), 6.86 (d, *J* = 2.0 Hz, 1H), 6.78 (dd, *J* = 8.4, 2.0 Hz, 1H), 3.79 (t, *J* = 7.2 Hz, 2H), 2.69 (q, *J* = 7.6 Hz, 2H), 2.60 (q, *J* = 7.6 Hz, 2H), 1.79 – 1.69 (m, 2H), 1.27 (t, *J* = 7.8 Hz, 3H), 1.24 – 1.20 (m, 3H), 0.94 (t, *J* = 7.4 Hz, 3H). ^{13}C NMR (100 MHz, $CDCl_3$): δ 153.74, 148.53, 145.80, 143.62, 142.82, 138.58, 132.42, 130.58, 129.12, 128.92, 128.65, 125.95, 125.78, 123.84, 122.48, 121.64, 119.23, 109.37, 105.96, 42.84, 28.64, 28.26 , 21.74 , 15.65, 15.58, 11.44. IR (KBr): 3032, 2962, 2929, 2868, 1716, 1598, 1494, 1398, 1271, 1226, 1182, 829，697，648 cm^{-1}. MS (EI, m/z): 475 $[M]^+$. HRMS (ESI): Calcd. for $C_{32}H_{33}N_3NaO$ $[M+Na]^+$: 498.2516; found:498.2520.

(34)ethyl4-(5-((4-(ethoxycarbonyl)phenyl)(phenyl)amino)-2-oxo-3-propyl-2,3-dihydro-1*H*-benzo[*d*]imidazol-1-yl)benzoate (5kg)

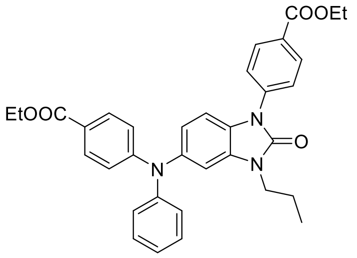

Brownish oil liquid, (77 mg, 55% yield); ^{1}H NMR (400 MHz, $CDCl_3$): δ 8.21 (d, *J* = 8.6 Hz, 2H), 7.87 (d, *J* = 8.8 Hz, 2H), 7.68 (d, *J* = 8.6 Hz, 2H), 7.31 (t, *J* = 7.8 Hz, 2H), 7.18 – 7.08 (m, 4H), 6.98 (d, *J* = 8.8 Hz, 2H), 6.90 – 6.85 (m, 2H), 4.41 (q, *J* = 7.0 Hz, 2H), 4.34 (q, *J* = 7.2 Hz, 2H), 3.82 (t, *J* = 7.2 Hz, 2H), 1.81 – 1.71 (m, 2H), 1.42 (t, *J* = 7.2 Hz, 3H), 1.37 (t, *J* = 7.2 Hz, 3H), 0.97 (t, *J* = 7.4 Hz, 3H).^{13}C NMR (100 MHz, $CDCl_3$): δ 166.42, 165.86, 153.18, 152.12, 146.81, 141.93, 138.89, 130.98, 130.91, 129.62, 129.17, 125.91, 125.24, 124.98, 124.30, 122.45, 120.31, 119.49, 109.83, 106.98, 61.24, 60.60, 43.01, 21.66, 14.48, 14.41, 11.42. IR (KBr): 3001, 2936, 2879, 1725, 1588, 1511, 1462, 1421, 1265, 1223, 1141, 1084, 1029, 854, 807, 748 cm^{-1}. MS (EI, m/z): 563 $[M]^+$. HRMS (ESI): Calcd. for $C_{34}H_{33}N_3NaO_5$ $[M+Na]^+$: 586.2312; found: 586.2313.

(35)5-(phenyl(4-(trifluoromethyl)phenyl)amino)-3-propyl-1-(4-(trifluoromethyl)phenyl)-1,3-dihydro-2*H*-benzo[*d*]imidazol-2-one (5lg)

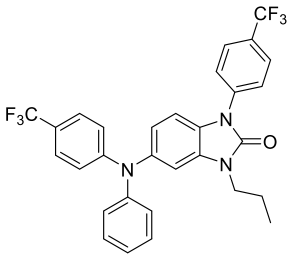

Brownish solid, (58 mg, 42% yield), m.p: 119-126 °C; ^{1}H NMR (400 MHz, $CDCl_3$): δ 7.76 (dd, *J* = 24.6, 8.6 Hz, 4H), 7.42 (d, *J* = 8.6 Hz, 2H), 7.32 (t, *J* = 7.8 Hz, 2H), 7.16 – 7.04 (m, 6H), 6.92 – 6.85 (m, 2H), 3.83 (t, *J* = 7.2 Hz, 2H), 1.82 – 1.72 (m, 2H), 0.97 (t, *J* = 7.4 Hz, 3H). ^{13}C NMR (100 MHz, $CDCl_3$): δ 153.29, 151.18, 146.98, 142.20, 138.15, 131.10, 129.82, 129.75, 129.58, 129.25, 126.81 (q, *J* = 3.7 Hz), 126.49, 126.44 (q, *J* = 3.7 Hz), 126.13, 125.97, 125.84, 125.65, 125.61, 125.32, 125.04, 124.28, 123.27, 122.87, 122.62, 122.54, 121.05, 120.34, 120.30, 119.99, 109.79, 107.02, 43.13, 21.74, 11.48. IR (KBr): 3064, 2973, 2940, 2879, 1714, 1588, 1553, 1490, 1396, 1321, 1223, 1161, 1110, 827, 742, 695 cm^{-1}. MS (EI, m/z): 555 $[M]^+$. HRMS (ESI): Calcd. for $C_{30}H_{23}F_2N_3NaO$ $[M+Na]^+$: 578.1638; found:578.1633.

(36)6-(di-o-tolylamino)-1-ethyl-4-methyl-3-(o-tolyl)-1,3-dihydro-2*H*-benzo[*d*]imidazol-2-one (5mf)

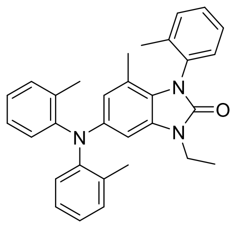

Brownish oil liquid, (71 mg, 62% yield); ^{1}H NMR (400 MHz, $CDCl_3$): δ 7.34 – 7.30 (m, 3H), 7.29 – 7.26 (m, 1H), 7.19 – 7.16 (m, 2H), 7.14 – 7.09 (m, 2H), 7.04 (td, *J* = 7.2, 1.4 Hz, 2H), 6.95 (dd, *J* = 7.8, 1.0 Hz, 2H), 6.31 (d, *J* = 2.0 Hz, 1H), 6.18 (d, *J* = 1.2 Hz, 1H), 3.86 – 3.78 (m, 2H), 2.16 (s, 3H), 2.01 (s, 6H), 1.60 (s, 3H), 1.23 (t, *J* = 7.2 Hz, 3H). ^{13}C NMR (100 MHz, $CDCl_3$): δ 153.78, 146.89, 143.92, 137.76, 135.44, 134.23, 131.66, 130.70, 130.26, 129.88, 129.07, 126.94, 126.85, 126.64, 124.28, 122.30, 120.45, 117.71, 99.54, 35.87, 19.03, 17.82, 17.14, 13.55. IR (KBr): 3060, 3021, 2968, 2926, 2855, 1712, 1620, 1601, 1489, 1402, 1378, 1265, 1234, 1116, 1059, 750, 720, 653, 625 cm^{-1}. MS (EI, m/z): 461 $[M]^+$. HRMS (ESI): Calcd. for $C_{31}H_{31}N_3NaO$ $[M+Na]^+$: 484.2359; found: 484.22359.

(37)3-ethyl-1-(4-methoxy-2-methylphenyl)-5-((4-methoxy-2-methylphenyl)(phenyl)amino)-1,3-dihydro-2*H*-benzo[*d*]imidazol-2-one (5nf)

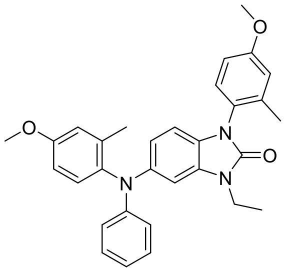

Brownish solid, (74 mg, 60% yield); m.p: 77-78 °C; ^{1}H NMR (400 MHz, $CDCl_3$): δ 7.15 – 7.22(m, 3H), 7.08 (d, *J* = 8.8 Hz, 1H), 6.88 (d, *J* = 7.6 Hz, 3H), 6.86 – 6.78 (m, 4H), 6.76 (d, *J* = 8.8 Hz, 1H), 6.66 (d, *J* = 8.4 Hz, 1H), 6.51 (d, *J* = 8.4 Hz, 1H), 3.89 (q, *J* = 7.2 Hz, 2H), 3.83 (s, 3H), 3.81 (s, 3H), 2.15 (s, 3H), 2.08 (s, 3H), 1.30 (t, *J* = 7.0 Hz, 3H). ^{13}C NMR (100 MHz, $CDCl_3$): δ 159.84, 157.72, 153.63, 148.58, 142.56, 138.70, 138.41, 138.00, 130.68, 129.95, 129.72, 129.11, 126.10, 126.07, 120.17, 119.53, 116.75, 116.66, 116.58, 112.85, 112.47, 109.00, 103.24, 55.59, 55.50, 36.08, 18.95, 18.30, 13.78. IR (KBr): 3060, 2956, 2926, 2852, 1710, 1625, 1600, 1492, 1403, 1300, 1232, 1193, 1160, 1113, 1044, 804, 749, 695 cm^{-1}. MS (EI, m/z): 493 $[M]^+$. HRMS (ESI): Calcd. for $C_{31}H_{32}N_3NO_3$ $[M+H]^+$: 494.2438; found: 494.2432.

(38)1-(3,4-dimethylphenyl)-5-((3,4-dimethylphenyl)(phenyl)amino)-3-propyl-1,3-dihydro-2*H*-benzo[*d*]imidazol-2-one (5og)

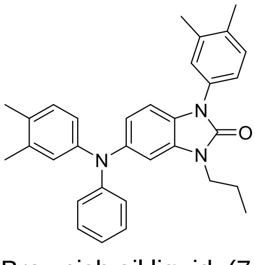

Brownish oil liquid, (74 mg, 62% yield); ^{1}H NMR (400 MHz, $CDCl_3$): δ 7.31 (s, 1H), 7.24 (s, 2H),

7.22 – 7.17 (m, 2H), 7.05 – 6.98 (m, 3H), 6.91 (t, *J* = 7.6 Hz, 3H), 6.87 – 6.81 (m, 2H), 6.76 (dd, *J* = 8.4, 2.0 Hz, 1H), 3.79 (t, *J* = 7.2 Hz, 2H), 2.30 (d, *J* = 2.8 Hz, 6H), 2.21 (s, 3H), 2.17 (s, 3H), 1.79 – 1.70 (m, 2H), 0.95 (t, *J* = 7.4 Hz, 3H). ^{13}C NMR (100 MHz, $CDCl_3$): δ 153.80, 148.66, 145.93, 142.82, 137.95, 137.53, 136.13, 132.47, 131.04, 130.54, 130.42, 129.09, 127.09, 126.04, 125.35, 123.31, 122.40, 121.62, 121.47, 119.13, 109.34, 105.85, 42.84, 21.76, 19.95, 19.54, 19.17, 11.45. IR (KBr): 3026, 2963, 2926, 2868, 1715, 1599, 1495, 1398, 1304, 1272, 809, 741, 702 cm^{-1}. MS (EI, m/z): 475 $[M]^+$. HRMS (ESI): Calcd. for $C_{32}H_{33}N_3NaO$ $[M+Na]^+$: 498.2516; found:498.2517.

(39)3-ethyl-1-isopropyl-5-(isopropyl(phenyl)amino)-1,3-dihydro-2*H*-benzo[*d*]imidazol-2-one (5pf)

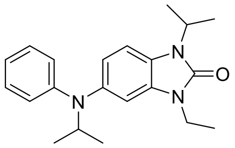

Brownish oil liquid, (71 mg, 84% yield); ^{1}H NMR (400 MHz, $CDCl_3$): δ 7.20 – 7.11 (m, 3H), 6.79 (dd, *J* = 8.2, 1.8 Hz, 1H), 6.76 – 6.69 (m, 2H), 6.64 (d, *J* = 8.2 Hz, 2H), 4.77 (hept, *J* = 7.0 Hz, 1H), 4.35 (hept, *J* = 6.6 Hz, 1H), 3.89 (q, *J* = 7.2 Hz, 2H), 1.57 (d, *J* = 7.0 Hz, 6H), 1.30 (t, *J* = 7.2 Hz, 3H), 1.19 (d, *J* = 6.6 Hz, 6H). ^{13}C NMR (100 MHz, $CDCl_3$): δ 153.62, 149.23, 136.70, 130.12, 129.02, 126.33, 122.91, 117.33, 115.20, 109.72, 109.43, 47.85, 45.02, 35.84, 21.08, 20.46, 13.65. IR (KBr): 3059, 2975, 2875, 1705, 1595, 1494, 1401, 1385, 1359, 1305, 749 cm^{-1}. MS (EI, m/z): 337 $[M]^+$. HRMS (ESI): Calcd. for $C_{21}H_{28}N_3O$ $[M+H]^+$: 338.2227; found:338.2232.

Supplemental Reference.

Yang, T. F., Chiu, K. Y., Cheng, H. C., Lee, Y. W., Kuo, M. Y. and Su, Y. O. (2012). Studies on the structure of N-phenyl-substituted hexaaza[1_6]paracyclophane: synthesis, electrochemical properties, and theoretical calculation. J. Org. Chem. *77*, 8627-8633.

Shi, R., Lu, L., Zhang, H., Chen, B., Sha, Y., Liu, C., and Lei, A. (2013). Palladium/copper catalyzed oxidative C–H alkenylation/N-dealkylative carbonylation of tertiary Anilines. Angew. Chem. Int. Ed. *52*, 10582 –10585.
